# Measures of attributes of locomotor capacity in older people: a systematic literature review following the COSMIN methodology

**DOI:** 10.1093/ageing/afad139

**Published:** 2023-10-30

**Authors:** Germain Honvo, Shaun Sabico, Nicola Veronese, Olivier Bruyère, René Rizzoli, Jotheeswaran Amuthavalli Thiyagarajan, Christopher Mikton, Theresa Diaz, Cyrus Cooper, Jean-Yves Reginster

**Affiliations:** World Health Organization (WHO) Collaborating Center for Epidemiology of Musculoskeletal Health and Ageing, University of Liège, Liège, Belgium; Division of Public Health, Epidemiology and Health Economics, University of Liège, Liège, Belgium; Chair for Biomarkers of Chronic Diseases, Biochemistry Department, College of Science, King Saud University, Riyadh, Kingdom of Saudi Arabia; Chair for Biomarkers of Chronic Diseases, Biochemistry Department, College of Science, King Saud University, Riyadh, Kingdom of Saudi Arabia; Geriatric Unit, Department of Internal Medicine and Geriatrics, University of Palermo, Palermo, Italy; World Health Organization (WHO) Collaborating Center for Epidemiology of Musculoskeletal Health and Ageing, University of Liège, Liège, Belgium; Division of Public Health, Epidemiology and Health Economics, University of Liège, Liège, Belgium; World Health Organization (WHO) Collaborating Center for Epidemiology of Musculoskeletal Health and Ageing, University of Liège, Liège, Belgium; Division of Bone Diseases, Geneva University Hospitals and Faculty of Medicine, Geneva, Switzerland; Ageing and Health Unit, Department of Maternal, Newborn, Child, Adolescent Health and Ageing, World Health Organization (WHO), Geneva, Switzerland; Demographic Change and Healthy Aging Unit, Social Determinants of Health, World Health Organization, Geneva, Switzerland; Epidemiology, Monitoring and Evaluation Unit, Maternal, Newborn, Child, Adolescent Health and Ageing, World Health Organization, Geneva, Switzerland; World Health Organization (WHO) Collaborating Center for Epidemiology of Musculoskeletal Health and Ageing, University of Liège, Liège, Belgium; MRC Lifecourse Epidemiology Unit, University of Southampton, Southampton General Hospital, Southampton, UK; World Health Organization (WHO) Collaborating Center for Epidemiology of Musculoskeletal Health and Ageing, University of Liège, Liège, Belgium; Division of Public Health, Epidemiology and Health Economics, University of Liège, Liège, Belgium; Chair for Biomarkers of Chronic Diseases, Biochemistry Department, College of Science, King Saud University, Riyadh, Kingdom of Saudi Arabia

**Keywords:** locomotor capacity, balance, endurance, muscle strength, muscle power, muscle function, joint function, screening or assessment tools, measurement properties, older people, systematic review

## Abstract

**Background:**

Locomotor capacity (LC) is an important domain of intrinsic capacity and key determinant of functional ability and well-being in older age. The United Nations Decade of Healthy Ageing (2021–2030) calls for strengthening data and research on healthy ageing, including the measurement of older persons' LC. To advance the measurement and monitoring of LC, there is pressing need to identify valid and reliable measures.

**Objective:**

To identify all the available tools that were validated for measurement of LC or of its specific attributes in older people and to assess the methodological quality of the studies and measurement properties of the tools.

**Design:**

Systematic review.

**Setting:**

Anywhere (Community-dwelling; long-term care facility; etc.)

**Subjects:**

Older people.

**Methods:**

We used highly sensitive search strategies to search the following databases: Medline, Embase, Scopus, CINAHL and PsycINFO. The study was conducted following the COnsensus-based Standards for the selection of health Measurement Instruments (COSMIN) methodology for systematic review of outcome measurement instruments.

**Results:**

A total of 125 studies were included, which assessed tools for balance (n = 84), muscle power (n = 12), muscle strength (n = 32, including four studies about tools for balance and muscle power) and endurance (n = 1). No studies on tools for muscle function, joint function, or locomotor capacity overall, were retrieved. We identified 69 clinician-report or objective assessment tools for balance, 30 for muscle strength, 12 for muscle power and 1 endurance assessment tool. The GRADE assessment of quality of evidence showed that only a few tools have high quality evidence for both sufficient validity and reliability: The Balance Evaluation Systems Test (BESTest), the Mini-Balance Evaluation Systems Test (Mini-BESTest), the Berg Balance Scale (BBS) and the Timed Up and Go (TUG) test.

**Conclusions:**

A few tools with high quality evidence for sufficient validity and reliability are currently available for balance assessment in older people that may be recommended for use in clinical and research settings. Further validation studies are required for muscle strength, muscle power and endurance assessment tools.

## Key Points

- We identified 69 tools for balance, 30 for muscle strength, 12 for muscle power and 1 endurance assessment tool.
- Only a few tools with high quality evidence for sufficient validity and reliability are available to assess balance.
- Further validation studies are required for muscle strength, muscle power and endurance assessment tools.
- Several issues to be addressed by the WHO Locomotor Capacity Working Group were identified.

## Introduction

Healthy ageing is defined by the World Health Organization (WHO) as ‘the process of developing and maintaining the functional ability that enables wellbeing in older age’ [1, 2]. Functional ability comprises the health-related attributes that enable people to be and to do what they have reason to value. It is made up of the intrinsic capacity of the individual, relevant environmental characteristics and the interactions between the individual and these characteristics. Intrinsic capacity is the composite of all the physical and mental capacities of an individual, including visual and hearing capacities, cognitive and psychological capacities, vitality and locomotor capacity [3].

Locomotor capacity is an important domain of intrinsic capacity and key determinant for functional ability and wellbeing in older age. The WHO expert working group on locomotor capacity, consisting of a fifty clinicians and scientists in fields of musculoskeletal health and ageing, from all regions of the world, proposed a working definition of locomotor capacity as ‘a state (static or dynamic over time) of the musculoskeletal system that encompasses endurance, balance, muscle strength, muscle function, muscle power and a joint function of the body’ [4]. As a next step, this systematic review was conducted to identify valid, reliable and responsive measures of locomotor capacity and of its attributes.

The United Nations Decade of Healthy Ageing (2021–2030), endorsed by the World Health Assembly and the United Nations (UN) General Assembly, recognises the importance of strengthening data for measurement, monitoring and evaluation, as a facilitator of progress assessment against goals in the prioritised four action areas [5]. These key action areas include: (i) changing how we think, feel and act towards age and ageing (i.e. combatting ageism); (ii) ensuring that communities foster the abilities of older people (i.e. developing age-friendly environments); (iii) delivering person-centred integrated care and primary health services that are responsive to older people and (iv) providing access to long-term care for older people who need it [6]. A systematic assessment of best available measures of locomotor capacity is therefore essential to develop recommendations for use in population surveys and routine health information, as well as for individual assessments of patients by clinicians.

Evidence suggests that systematic literature reviews can help identify the available outcome measures in specific fields, thus providing a comprehensive overview of their measurement properties as well as supporting evidence-based recommendations for use in research and clinical practice [7]. A recent systematic review was published that has identified commonly used tests of balance and strength and evaluated their measurement properties in young seniors (aged 60–70 years) [8]. However, to the best of our knowledge, no systematic review has comprehensively assessed measurement tools for all attributes of locomotor capacity considering all stages of older age.

### Research question, objectives and purpose

The research question for this systematic literature review is: What are the available and validated tools to measure the specific attributes of locomotor capacity, or locomotor capacity overall, in older people?

The objectives of the study were to comprehensively review the available outcome measurement instruments that were validated for specific attributes of locomotor capacity or for locomotor capacity overall in older people and to assess the methodological quality of the studies and measurement properties of the tools. The findings of this systematic review will support WHO in developing evidence-based recommendations for use of these tools in population surveys and data collection in health care facilities.

## Methods

### Guidelines and protocol registration

This systematic review was conducted following the COnsensus-based Standards for the selection of health Measurement Instruments (COSMIN) methodology [7]. Recommendations in the Cochrane handbook for systematic literature reviews were also followed for screening and selection of studies [9]. The current report follows the Preferred Reporting Items for Systematic Reviews and Meta-Analyses (PRISMA) guidelines [10].

The protocol of this study was registered in the International Prospective Register of Systematic Reviews (PROSPERO: Registration number, CRD42022318959).

The Covidence online software (https://www.covidence.org/) was used to manage the entire study selection process, from title/abstract screening to full-text selection.

### Information sources and search strategies

To conduct this systematic literature review, several bibliographic databases were comprehensively searched (from inception to April 18, 2022) using detailed and highly sensitive search strategies tailored to the syntax of each database. These databases include: Medline (via Ovid), Embase, Scopus, Cumulative Index to Nursing and Allied Health Literature (CINAHL) and PsycINFO (via Ovid). The search for individual studies in the databases was subsequently supplemented by manual search of Google and of references of relevant systematic reviews that were identified, along with references of included studies.

To guide the identification of adequate keywords to build the search strategies, the research question was framed into the ‘Participants, Intervention, Comparison, Outcome’ (PICO) format, following the framework proposed in the WHO handbook for guideline development, section on systematic review question formulation [11]. The PICO format question is as follows: What are the available and validated screening or assessment tools (I) for measuring endurance, balance, muscle strength, muscle function, muscle power and joint function or locomotor capacity overall (O) in older people (aged 60 years and older) (P)?

The terms of this PICO format question (i.e. P, I and O) were then adequately combined (with Boolean operators) to build the search strategies, using free vocabulary words and controlled terms tailored to databases. To search for records relating to screening or assessment tools (I), we used the Ovid search filter for patient-reported outcome measurement (PROM) that was developed by the Oxford ‘PROM Group Construct & Instrument Type Filters’ [12], which we adapted to fit best with our review question and to limit background noise while being sensitive. We also used additional search strings for ‘measurement tool’ (I) developed by our review team. In the end, the PubMed exclusion filter developed by Terwee et al. [13] was adapted for Ovid and used to remove irrelevant records, such as case reports and animal studies, from the search results. The exclusion filter was used exactly as indicated by Terwee et al. [13]. The search strategies developed for all databases are provided as Supplementary material to this paper (Appendix 1).

### Eligibility criteria

#### Inclusion criteria

Individual studies on screening or assessment tools for both objective or self-reported assessment of specific attributes of locomotor capacity (i.e. endurance, balance, muscle strength, muscle function, muscle power, joint function), or of locomotor capacity overall, in older people (aged 60 years and older), were included in this systematic literature review. The specific selection criteria regarding the study population in articles were as follows: a) studies include older people aged 60 years of age or older or b) studies with a mean age of sample above 65 years or c) studies with at least 50% of the sample (defined as majority [14]) with older people aged 60 years or older or d) studies separately report results on participants aged 60 years or older. Original studies on development and validation of tools, aiming at evaluating one or more measurements properties, as well as studies reporting their translation, cross-cultural adaptation and validation in other languages or settings, or in older people were included. Finally, studies examining the measurement properties of more than one measurement instrument for the same attribute or for several distinct attributes of locomotor capacity were also included.

#### Exclusion criteria

Validation studies in populations with specific medical conditions (e.g. Parkinson’s disease, stroke, etc.), even if in older people, were excluded from this systematic literature review. Likewise, studies that did not report data on measurement properties of tools were excluded, as were studies in which a measurement tool was used in a validation study of another instrument (i.e. the instrument to be considered is the one that is being validated). Articles in which a measurement tool is used only for outcome measurement in an experimental study were also considered ineligible for inclusion in this review, as were review papers (systematic or not) and editorials. Finally, abstracts reporting studies on measurement tools without full-text reports, and articles in languages other than English were excluded.

### Study selection

We followed recommendations in the Cochrane handbook for Systematic reviews to select studies based on title/abstract first, then on full manuscripts [9]. The title/abstract selection was independently done by three review authors (GH, SS, NV), and the full text selection by two members of the review team (GH, SS), with consensus meetings to discuss any disagreements. A third member of the review team (NV) was involved for final decision on full text selection, when necessary.

### Data collection and data items

All the data were extracted by one reviewer (GH), then the extractions were independently checked by a second review author (SS) for identification and correction of inaccuracies.

Items collected from the retrieved full-text articles were information for identification of the manuscript, data on the characteristics of the study population, as well as data on characteristics of the tools and on their measurement properties (e.g. reliability, criterion validity, etc.). These data were collected using standard data extraction forms, adapted from templates provided in the *COSMIN methodology user manual* [15].

Data on measurement properties were extracted according to the COSMIN taxonomy and terminology of measurement properties for outcome measures [16], as recommended by the COSMIN guideline for systematic reviews [15]. For example, a result of a validation study was extracted as ‘concurrent validity’ (i.e. criterion validity) only if the tool was validated against a renowned gold standard, as per COSMIN definitions [16]. Validation against any other tool (that is not recognised as gold standard) was therefore considered ‘convergent validity’, even if the authors reported such property as being ‘concurrent validity’. Also, when measurement properties were assessed but not named (e.g. validation against a non-gold-standard tool measuring the same construct, not formally named ‘convergent validity’), data were extracted assuming the type of measurement property according to the COSMIN terminology.

### Assessment of risk of bias in the included studies

The methodological quality of each included studies was evaluated using the COSMIN Risk of Bias checklist for systematic reviews of Patient-Reported Outcome Measures [17], by completing the adequate boxes of the checklist. The risk of bias assessment was performed by the lead author (GH) for all the included studies and double-checked by the same author several weeks later. Then, a second review author (NV) checked again these assessments.

As this systematic review included only clinician-reported outcome measures (ClinROMs, i.e. ratings based on clinician’s observations) and performance-based outcome measurement instruments (PerFOMs, i.e. objective assessments), we replaced the boxes on reliability and measurement error of the original COSMIN Risk of Bias checklist by the COSMIN Risk of Bias tool to assess the quality of studies on reliability and measurement error, as per COSMIN recommendations [18].

### Assessment of measurement properties of tools

The measurement properties of the included tools were assessed by applying the updated COSMIN criteria for good measurement properties [7]. For each included studies and tools, each measurement property was rated as either sufficient (+), insufficient (−), or indeterminate (?). Measurement properties for all the included studies were assessed by the lead author (GH) and cross-checked by another member of the review team (SS).

Regarding hypotheses testing for construct validity and responsiveness, we pre-formulated hypotheses to evaluate the results of the included studies, so that all results are compared against the same set of hypotheses, as recommended by the COSMIN methodology [7]. For convergent validity, the following hypotheses were formulated: 1) Correlations (Pearson’s, Spearman’s correlations, or Intra-class correlation coefficients [ICC]) or Kappa coefficient for concordance with instruments measuring similar constructs should be >0.50; 2) Correlations with instruments measuring related but not similar constructs (e.g. a balance assessment tool validated against a gait speed test) should be between 0.30 and 0.50; 3) Correlations with instruments measuring dissimilar constructs should be <0.30. For discriminative (know-group) validity, scores of instruments should be significantly different between relevant subgroups (e.g. patients with history of falls versus patients without history of falls, for balance assessment tools), whatever the statistical method used for comparison. In the end, for responsiveness, area under the curve (AUC) with an external measure of change used as the gold standard should be ≥0.70, as per COSMIN methodology [7].

For ICC or other correlation values, when range of values (e.g. ICC = 0.52–0.89) or multiple values for the same measurement property (e.g. ICC = 0.50 for inter-rater, and 0.88 for test–retest reliability) were available from a single study and tool, the best value was considered for measurement property rating.

### Data synthesis and GRADE assessment of findings

Data extracted from the retrieved articles were summarised in tables presenting the main characteristics of the included studies and tools, as well as information on the measurement properties of the tools. Qualitative summaries of results of measurement properties were presented, based on data from all the included studies on each specific tool, according to the COSMIN guideline [7, 15]. Overall measurement property ratings were performed for each tool, considering the summary results, as recommended by the COSMIN guideline [7, 15].

For tools with at least two validation studies included, we assessed the quality of evidence on measurement properties using the modified GRADE (Grading of Recommendations, Assessment, Development and Evaluations) approach, as described by the COSMIN guideline [7, 15].

## Results

### Literature search result

**Figure 1:**
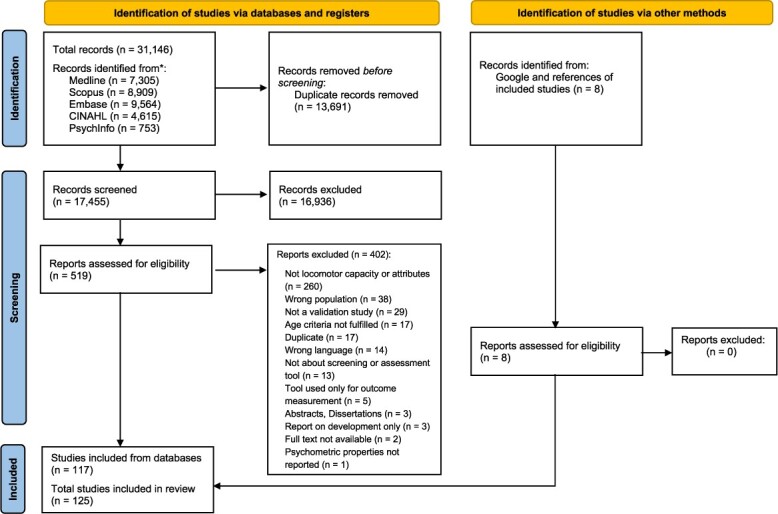
PRISMA flow-chart of the systematic review.

From 31,146 records retrieved from databases search, 117 individual studies were included, after exclusions. Eight (8) additional studies were found from manual search of Google and of references of studies included from databases search, bearing the total number of included studies to 125. An overview of the flow of studies selection with reasons for full texts exclusions is presented in Figure 1.

Most of the included studies (n = 84) were on balance assessment tools [19–102], and several studies evaluated multiple balance tools (two to four tools). Twelve (12) studies assessed muscle power tools [103–114]. Muscle strength assessment tools were evaluated in 32 studies [73, 108, 112, 113, 115–142], among which three studies [135, 138, 141] evaluated multiple tools for muscle strength. Two (2) of the studies on muscle strength also assessed another instrument for muscle power [108, 113], while one study assessed an instrument for both muscle strength and power [112], and one other study evaluated the measurement properties of a same tool to assess both muscle strength and balance [73]. Ultimately, only one study was retrieved as a validation study of a tool to assess endurance [143] in older people. The literature search returned no studies validating tools to assess muscle function, joint function or locomotor capacity overall, in older people.

### Characteristics of included studies

The main characteristics of the included studies and populations are summarised, and separately reported for balance, muscle strength, muscle power and endurance assessment tools in Appendix 2.

**Figure 2:**
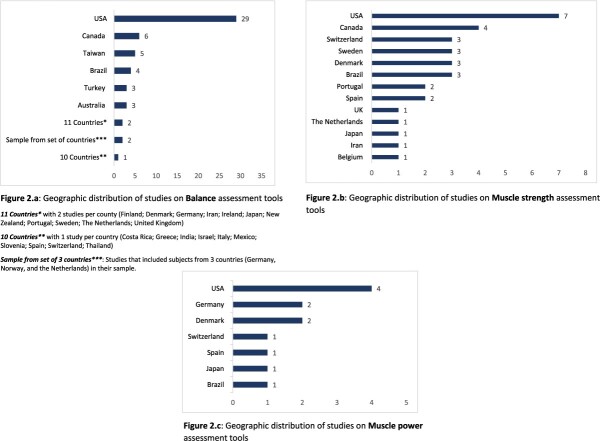
Geographic distribution of studies.

Appendix 2a describes the characteristics of studies on **balance** tools. The mean age of participants in most of these studies was ≥70 years. Only about a quarter of studies (n = 22) had a sample size of 100 or more. Most studies included more women (> 50% of the sample) than men, while five studies included women only [26, 36, 55, 57, 95] and 11 did not report the percentage of female included. In 80 out of the 84 studies on balance assessment tools, participants were recruited in the community and/or long-term care facilities (i.e. nursing homes, residential care facilities; homes for the elderly; etc.), with the dominant setting being the community. Three studies recruited patients from other settings such as rehabilitation centers and day unit for elderly [38, 47, 53], and the setting was not reported in one study [43]. Figure 2.a shows the geographical distribution of studies, with number of studies by countries: Of the 84 studies on balance assessment tools, 35 studies were conducted in North America (USA and Canada).

The characteristics of the 32 studies on **muscle strength** assessment tools are presented in Appendix 2b. In 75% of these studies, the mean age of patients was >70 years. As for balance tools studies, women were more represented in the studies on muscle strength tools than men: The percentage of female was >50% in 21 studies, while five studies included 100% women [108, 131, 133, 136, 138] and one study included men only [113]; three studies included ≤50% of women in their sample, and this information was not reported in two studies. Participants were mainly recruited from the community, but also from long-term care facilities. Most of the studies originated from USA (seven studies) and Canada (four studies) (Figure 2.b).

With regard to studies on **muscle power** assessment tools (Appendix 2c), four included female only [106, 108–110], and one male only [113]; in almost all of the other studies, the percentage of female was >50%. The mean age was >70 years in nearly all the studies on muscle power assessment tools, which included patient recruited mainly from the community (9 of 12 studies). The three other studies included patients from long-term care facilities (two studies) and a geriatric clinic. More than 80% of these studies were conducted in USA and European countries (Figure 2.c).

The only one study retrieved on **endurance** assessment tools (Appendix 2d) was conducted in the USA and included 77 participants (73.1 ± 7.2 years) recruited from the community, who were mainly female (62.3%).

In summary, in terms of geographic distribution of validation studies, considering all attributes of locomotor capacity, it is worth noting that none of these studies were conducted, neither in African countries, nor in countries such as China, France, Russia and most studies come from the USA.

### Included tools and measurement properties

The characteristics of included tools, including details about attributes measured, mode of administration, number of items (where applicable) and scoring, are described in Appendix 3. The identified tools are PerFOMs (Objective assessment) or ClinROMs (Clinician report); no patient-reported outcomes measures (PROMs) were included. Here, we summarise the measurement properties of these instruments by attributes of locomotor capacity. Tables 1–4, reports summary results of measurement properties for all included tools, with overall quality ratings against the COSMIN criteria for good measurement properties [7]. The detailed data on measurement properties for all included tools (from individual studies), with quality ratings (by study and overall) are shown in Appendix 4(a-d).

**Table 1:** Measurement properties of balance assessment tools: Summary results with overall quality ratings: Summary results of measurement properties of tools with quality ratings

**Instrument**	**Reference**	**Reliability**			**Validity**	
		***Reliability***	***Measurement error***	***Internal consistency***	***Criterion validity***	***Hypothesis testing for construct validity***
The Balance Evaluation Systems Test (BESTest)	Anson, 2019 [19] Marques, 2016 [20] O’Hoski, 2015 [21] Viveiro, 2019 [22] Wang-Hsu, 2018 [23] Yingyongyudha, 2016 [24]	ICC = 0.77–0.99 (+)	MIC not defined (?)	N/R	r ≥ 0.70 (+)	Hypotheses confirmed (+)
The ***Spanish version*** of the BESTest (Spanish BESTest)	Dominguez-Olivan, 2020 [25]	ICC = 0.97 (+)	MIC not defined (?)	Criteria not met (?)	r < 0.70 (−)	r > 0.50 (+)
The Mini-Balance Evaluation Systems Test (Mini-BESTest)	Anson, 2019 [19] Marques, 2016 [20] O’Hoski, 2015 [21] Viveiro, 2019 [22] Yingyongyudha, 2016 [24]	ICC = 0.71–0.99 (+)	MIC not defined (?)	N/R	r ≥ 0.83 (+)	(±), for discriminative validity (+), for convergent validity
The ***Spanish version*** of the Mini-BESTest (Spanish Mini-BESTest)	Dominguez-Olivan, 2020 [25]	ICC = 0.79 (+)	MIC not defined (?)	Criteria not met (?)	r = 0.18 (−)	Hypothesis confirmed (+)
The modified Clinical test of Sensory Interaction in Balance (mCTSIB) of the Balance Platform Biodex Balance System (BBS)	Antoniadou, 2020 [26]	ICC = 0.628 (−)	N/R	N/R	N/R	r > 0.50 (+)
The Berg Balance Scale (BBS)	Berg, 1992a [27] Berg, 1992b [28] Bogle Thorbahn, 1996 [29] Harada, 1995 [30] Holbein-Jenny, 2005 [31] Marques, 2016 [20] Muir, 2008 [32] Pelicioni, 2022 [33] Viveiro, 2019 [22] Wang, 2006 [34] Yingyongyudha, 2016 [24]	ICC = 0.77–0.99 (+)	MIC not defined (?)	Criteria not met (?)	Indeterminate (?)	Hypotheses confirmed (+)
The ***Brazilian version*** of the Berg balance scale (Brazilian BBS)	Miyamoto, 2004 [35]	ICC ≥ 0.99 (+)	N/R	N/R	N/R	N/R
The Lateral Reach (LR) Test	Brauer, 1999 [36]	ICC = 0.999 (+)	N/R	N/R	r < 0.70 (−)	N/R
The Six-Spot Step Test	Brincks, 2021 [37]	ICC = 0.94–0.96 (+)	MIC not defined (?)	N/R	N/R	r > 0.50 (+)
The Functional reach (FR) test	Brooks, 2006 [38] Galhardas, 2020 [39] Giorgetti, 1998 [40] Lin, 2004 [41]	ICC ≥ 0.73 (+)	MIC not defined (?)	N/R	Indeterminate (?)	(+), for discriminative validity (−), for convergent validity
Gait Initiation Assessment	Chang, 1999 [42]	N/R	N/R	N/R	N/R	Hypothesis confirmed (+)
The modified Wii Fit balance board	Chang, 2013 [43]	ICC ≥ 0.93 (+)	N/R	N/R	N/R	N/R
The Stepping Threshold Test (STT)	Adams, 2021 [44]	N/R	N/R	N/R	N/R	Hypotheses not confirmed (−)
The Unstable board (DYJOC BOARD, SAKAI Medical Co., Ltd.)	Akizuki, 2018 [45]	N/R	N/R	N/R	N/R	Hypothesis not confirmed (−)
The limits of stability (LOS) test	Clark, 1997 [46]	ICC not reported (?)	MIC not defined (?)	N/R	N/R	N/R
The Four Square Step Test (FSST)	Cleary, 2017 [48] Işik, 2015 [49] Dite, 2002 [47]	ICC ≥ 0.98 (+)	N/R	N/R	Inconsistent (±)	(±), for convergent validity (+), for discriminative validity
The mediolateral balance assessment (MELBA) tool	Cofré Lizama, 2015 [50]	N/R	N/R	N/R	r < 0.70 (−)	N/R
The Spring Scale Test (SST)	DePasquale, 2009 [51]	ICC = 0.94 (+)	MIC not defined (?)	N/R	N/R	Hypotheses confirmed (+)
The Microsoft Xbox One Kinect (Kinect v2)	Eltoukhy, 2018 [52]	ICC > 0.75 (+)	MIC not defined (?)	N/R	r not reported (?)	N/R
The TURN 180 test	Fitzpatrick, 2005 [53] Ranji, 2020 [54]	ICC = 0.828 (+)	MIC not defined (?)	N/R	r not reported (?)	Hypothesis confirmed (+)
The Lower Quarter Y-Balance Test (LQ-YBT)	Freund, 2019 [55]	ICC ≥ 0.98 (+)	MIC not defined (?)	N/R	N/R	Hypothesis not confirmed (−)
The Narrow Path Walking Test (NPWT)	Gimmon, 2013 [56]	ICC = 0.77–0.92 (+)	MIC not defined (?)	N/R	N/R	Inconsistent (±)
One leg standing (OLS)	Giorgetti, 1998 [40] Lin, 2004 [41]	ICC ≥ 0.75 (+)	N/R	N/R	Indeterminate (?)	(+), for discriminative validity (−), for convergent validity
Tandem Gait (TG)	Giorgetti, 1998 [40]	ICC = 0.31 (−)	N/R	N/R	N/R	N/R
The five-times-sit-to-stand test (FTSST)	Goldberg, 2012 [57]	ICC = 0.95 (+)	MIC not defined (?)	N/R	N/R	Inconsistent (±)
The Maximum Step Length (MSL) test	Goldberg, 2010 [58]	ICC = 0.90–0.96 (+)	MIC not defined (?)	N/R	N/R	Inconsistent (±)
The Thirty Rapid-Step test (30-RST)	Goldberg, 2015 [59]	ICC = 0.85 (+)	MIC not defined (?)	N/R	N/R	Inconsistent (±)
The Community Balance and Mobility Scale (CBM)	Weber, 2018 [60]	ICC ≥ 0.97 (+)	N/R	Criteria not met (?)	N/R	Inconsistent (±)
The ***German***-Community Balance and Mobility Scale (German CBM)	Gordt, 2019 [61]	ICC ≥ 0.99 (+)	N/R	Criteria not met (?)	r > 0.70 (+)	Hypothesis confirmed (+)
The Shortened version of the Community Balance and Mobility Scale (s-CBM)	Gordt, 2020 [62]	N/R	N/R	Criteria met (+)	N/R	Inconsistent (±)
The ‘Step-Ex’ (New Development Technologies [NDT], Stockholm, Sweden)	Halvarsson, 2012 [63]	ICC = 0.71–0.87 (+)	MIC not defined (?)	N/R	N/R	N/R
Tinetti’s POMA balance subscale	Harada, 1995 [30] Lin, 2004 [41]	ICC ≥ 0.93 (+)	N/R	N/R	Indeterminate (?)	(+), for discriminative validity (−), for convergent validity
The Short Berg Balance Scale (BBS-9)	Hohtari-Kivimaki, 2012 [64]	N/R	N/R	Criteria met (+)	r < 0.70 (−)	N/R
The Multi-Directional Reach Test (MDRT)	Holbein-Jenny, 2005 [31] Newton, 2001 [65]	ICC = 0.83–0.98 (+)	N/R	Criteria not met (?)	Inconsistent (±)	(±), for convergent validity (−), for discriminative validity
The Kinect system (Kinect for Xbox 360™, Microsoft Corp, Seattle, WA, USA)	Hsiao, 2018 [66]	ICC ≥ 0.775 (+)	N/R	N/R	r < 0.70 (−)	r_S_ > 0.50 (+)
The ***Turkish version*** of Fullerton Advanced Balance (FAB-T) scale	Iyigun, 2018 [67]	ICC = 0.96 (+)	N/R	N/R	r_S_ = 0.70 (+)	N/R
The Fullerton Advanced Balance (FAB) Scale	Klein, 2011 [68] Rose, 2006 [69]	ICC not reported (?)	N/R	N/R	r ≥ 0.70 (+)	N/R
The parallel walk test	Lark, 2009 [70]	N/R	N/R	N/R	N/R	Hypotheses not confirmed (−)
The Timed Up and Go (TUG) test	Galhardas, 2020 [39] Lin, 2004 [41] Nightingale, 2019 [71] Pelicioni, 2022 [33] Yingyongyudha, 2016 [24]	ICC ≥ 0.83 (+)	MIC not defined (?)	N/R	r not reported (?)	Hypotheses confirmed (+)
The Balance Computerised Adaptive Testing (Balance CAT)	Lu, 2015 [72]	N/R	MIC not defined (?)	N/R	r = 0.90 (+)	Hypothesis confirmed (+)
The MyBalance test	Mansson, 2021 [73]	N/R	N/R	N/R	N/R	r not reported (?)
The Brief-Balance Evaluation Systems Test (Brief-BESTest)	Marques, 2016 [20] O’Hoski, 2015 [21] Viveiro, 2019 [22]	ICC = 0.82–0.99 (+)	MIC not defined (?)	N/R	r_S_ ≥ 0.83 (+)	(±), for discriminative validity (+), for convergent validity
The Functional Gait Assessment-***Brazil*** (FGA- Brazil)	Marques, 2021 [74] Kirkwood, 2021 [75]	ICC > 0.90 (+)	MIC not defined (?)	Criteria not met (?)	r_S_ = 0.80 (+)	Hypothesis confirmed (+)
The ‘Get-up and Go’ Test	Mathias, 1986 [76]	ICC not reported (?)	N/R	N/R	r < 0.70 (−)	N/R
The apparatus for assessment of postural responses	Matjacic, 2010 [77]	N/R	N/R	N/R	r < 0.70 (−)	Hypothesis confirmed (+)
A comprehensive set of inertial sensor measures of postural sway (The Balance Score (BS) & The Weighted Balance Score (WBS))	Mcmanus, 2022 [78]	ICC ≥ 0.75 (+)	N/R	N/R	N/R	Inconsistent (±)
The Modified Version of the Community Balance and Mobility Scale (CBMS-Home)	Ng, 2021 [79]	ICC = 0.95 (+)	MIC not defined (?)	Criteria not met (?)	N/R	Inconsistent (±)
The Pavia Instrumented Tinetti Test (PITT)	Panella, 2008 [80]	N/R	N/R	Criteria not met (?)	r not reported (?)	Hypotheses confirmed (+)
The Dynamic Gait Index (DGI)	Pelicioni, 2022 [33]	ICC ≥ 0.85 (+)	N/R	N/R	N/R	r > 0.50 (+)
The ***Danish Version*** of the Dynamic Gait Index (Danish DGI)	Jønsson, 2011 [98]	ICC = 0.82–0.89 (+)	MIC not defined (?)	N/R	N/R	N/R
The Functional Gait Assessment (FGA)	Pelicioni, 2022 [33] Wrisley, 2010 [99] Beninato, 2016 [100] $	ICC ≥ 0.80 (+)	N/R	N/R	r = 0.84 (+)	Hypotheses confirmed (+)
The NIH Toolbox® Standing Balance Test	Peller, 2022 [81]	ICC = 0.84 (+)	MIC not defined (?)	N/R	r < 0.70 (−)	N/R
The Biodex SD (Biodex Medical Systems, Shirley NY)	Riemann, 2017 [82]	ICC = 0.74–0.86 (+)	MIC not defined (?)	N/R	N/R	N/R
The Balance Scale (by Roberts)	Roberts, 1987 [83]	N/R	N/R	Cronbach’s α not reported (?)	N/R	N/R
The ***Turkish Version*** of the Berg Balance Scale (BBS)	Sahin, 2008 [84]	ICC = 0.97–0.98 (+)	N/R	Criteria not met (?)	N/R	r > 0.50 (+)
The ***Persian version*** of the Berg Balance Scale (BBS)	Salavati, 2012 [85]	ICC = 0.93–0.95 (+)	N/R	Criteria not met (?)	N/R	r > 0.50 (+)
The Nintendo Wii Fit *exergame*	Sato, 2021 [86]	N/R	N/R	N/R	N/R	r > 0.50 (+)
The Wii Stillness (WST) Test	Simms, 2020 [88]	N/R	N/R	N/R	r < 0.70 (−)	N/R
The short form of the Fullerton Advanced Balance (SF-FAB) scale	Sinaei, 2021 [89]	ICC = 0.92–0.99 (+)	MIC not defined (?)	Criteria not met (?)	r not reported (?)	Hypothesis confirmed (+)
The ‘balance meter’	Stokes, 1998 [90]	ICC not reported (?)	MIC not defined (?)	N/R	N/R	Inconsistent (±)
The AMTI Accusway system for balance and postural sway measurement (Advanced Mechanical Technology, Inc., Watertown, Massachusetts)	Swanenburg, 2008 [91]	ICC = 0.52–0.89 (+)	MIC not defined (?)	N/R	N/R	N/R
A dual-task computer game-based platform (TGP)	Szturm, 2015 [92]	ICC = 0.55–0.7 (+)	MIC not defined (?)	N/R	N/R	N/R
The Modified Bathroom Scale	Vermeulen, 2012 [93]	N/R	N/R	N/R	N/R	Inconsistent (±)
The instrumented modified Clinical Test of Sensory Interaction on Balance (i-mCTSIB) utilising the Neurocom Very Simple Rehab (VSR) Sport force plate (Natus Medical Incorporated, Pleasanton, California).	Watson, 2021 [94]	ICC = 0.898 (+)	MIC not defined (?)	N/R	N/R	N/R
Models for estimating decline in balance using accelerometry-based gait features	Simila, 2017 [95]	N/R	N/R	N/R	r not reported (?)	r not reported (?)
The FICSIT Balance Scales (FICSIT-3 and FICSIT-4)	Rossiter-Fornoff, 1995 [96]	ICC not reported (?)	N/R	N/R	N/R	Hypothesis confirmed (+)
The Wii Balance Board™ (WBB)	Olvera-Chavez, 2013 [97] Scaglioni-Solano, 2014 [87]	ICC = 0.64–0.85 (+)	MIC not defined (?)	N/R	r not reported (?)	Hypothesis confirmed (+)
The Balance Tracking System (BTrackS)	Levy, 2018 [101]	ICC = 0.83 (+)	MIC not defined (?)	Criteria not met (?)	r ≥ 0.82 (+)	N/R
The NeuroCom Smart Equitest Research System (Natus Medical Inc, Pleasanton, California)	Harro, 2019 [102]	ICC ≥ 0.71 (+)	MIC not defined (?)	N/R	N/R	Hypotheses not confirmed (−)

$Only structural validity assessed.

Measurement property rating: sufficient (+), insufficient (−), inconsistent (±), indeterminate (?)

ABC, The Activities-specific Balance Confidence Scale; DSE, direction-sensitive evaluation (new strategy proposed by the authors in this study); 8LBS, The 8-level balance scale; APSI, anteroposterior stability index; MLSI, mediolateral stability index; PABAK, prevalence-adjusted bias-adjusted kappa; PASE, Physical Activity Scale for the Elderly; FAB, The Fullerton Advanced Balance scale; 3MTW, The three meter tandem walk; SRD, Smallest Real Difference; Falls Efficacy Scale-International (FES-I); SEM, Standard Error of Measurement; MDC, minimum detectable change; SD, standard deviation; CTSIB, Clinical Test of Sensory Interaction on Balance; FRT, The Functional Reach Test; SLS, single leg stance; N/R, not reported

#### Balance

A total of 69 tools were identified from the 84 included papers validating balance assessment instruments. Table 1 presents the summary results of measurement properties by specific tools, with overall ratings of measurement properties (for detailed data, see Appendix 4.a). Reliability, measurement error, criterion validity and convergent and discriminative validity (construct validity) were the most frequently reported measurement properties. A very few studies evaluated content validity and structural validity, but as these measurement properties were found to be marginally reported in the included studies, they were not assessed in this systematic review. No studies reported cross-cultural validity. Fifteen (15) of the identified balance tools were validated in at least two studies, including eight tools which were assessed in at least three studies. These eight tools, ranked by numbers of validation studies are:

- *The Berg Balance Scale (BBS)*, with evidence for sufficient reliability (ICC ≥ 0.77) and construct validity (convergent and discriminative validity), using summary results from 11 studies.
- *The Balance Evaluation Systems Test (BESTest)*, with evidence for sufficient reliability (ICC ≥ 0.77), criterion validity (r ≥ 0.70) and construct validity (convergent and discriminative validity), from six studies.
- *The Mini-Balance Evaluation Systems Test (Mini-BESTest)*, with evidence for sufficient reliability (ICC ≥ 0.71), criterion validity (r_S_ ≥ 0.83) and convergent validity, based on results of five studies. Inconsistent results were reported for discriminative validity.
- *The Timed Up and Go (TUG) test*, validated as a balance assessment tool in five studies, with summary results showing sufficient measurement properties, for reliability (ICC ≥ 0.83) and convergent and discriminative validity.
- *The Functional reach (FR) test*, validated by four studies and showing evidence for sufficient reliability (ICC ≥ 0.73) and discriminative validity, but not for convergent validity.
- *The Four Square Step Test (FSST)*, assessed in three studies, with sufficient reliability (ICC ≥ 0.98) and discriminative validity, but not for convergent validity.
- *The Brief-Balance Evaluation Systems Test (Brief-BESTest)*, with sufficient reliability (ICC ≥ 0.82), criterion validity (r_S_ ≥ 0.83) and convergent validity, based on summary data from three studies.
- *The Functional Gait Assessment (FGA)*, with three studies, including one study that assessed only structural validity. The two other studies provided, together, evidence for sufficient reliability (ICC ≥ 0.80), criterion validity (r = 0.84) and construct validity.

The seven other tools with two validation studies are the following: The Functional Gait Assessment-Brazil (FGA- Brazil), The Fullerton Advanced Balance (FAB) Scale, The TURN 180 test, The One leg standing (OLS) test, The Tinetti’s Performance-Oriented Mobility Assessment (POMA) balance subscale, The Multi-Directional Reach Test (MDRT) and The Wii Balance Board™ (WBB), with various measurement properties and ratings (Table 1 and Appendix 4.a).

**Table 2:** Measurement properties of muscle strength assessment tools: Summary results with overall quality ratings

**Instrument**	**Reference**	**Reliability**			**Validity**	
		***Reliability***	***Measurement error***	***Internal consistency***	***Criterion validity***	***Hypothesis testing for construct validity***
The JAMAR hand-held hydraulic dynamometer	Abizanda, 2012 [115] Silva, 2019 [116]	ICC = 0.90–0.99 (+)	MIC not defined (?)	N/R	N/R	r not reported (?)
A uni-axial load cell device	Alqahtani, 2019 [118]	ICC = 0.90–0.99 (+)	SDC > MIC (MCID) (−)	N/R	N/R	Inconsistent (±)
The calf-raise senior (CRS) test	Andre, 2016 [119]	ICC = 0.79–0.93 (+)	MIC not defined (?)	N/R	r ≥ 0.70 (+)	Hypothesis confirmed (+)
The Handheld Dynamometry (HHD): The Lafayette Manual Muscle Tester, Model # 01163, (Lafayette Instrument Inc., Lafayette, Indiana)	Arnold, 2010 [120] Bohannon, 2005 [121] Bohannon, 1997 [122] Martin, 2006 [123]	ICC = 0.76–0.98 (+)	MIC not defined (?)	Criteria not met (?)	r ≥ 0.70 (+)	N/R
The Nintendo Wii Balance Board (WBB)	Blomkvist, 2016 [124] Jorgensen, 2015 [125]	ICC = 0.96–0.97 (+)	MIC not defined (?)	N/R	N/R	ICC > 0.50 (+)
The Modified Sphygmomanometer Test (MST)	Brito, 2022 [126]	ICC = 0.80–0.99 (+)	MIC not defined (?)	N/R	N/R	r > 0.50 (+)
MicroFET2 hand-held dynamometer (Hoggan Indiustries, Inc., West Jordan, UT, USA)	Buckinx, 2017 [117]	ICC = 0.62 – 0.87 (+)	MIC not defined (?)	N/R	N/R	N/R
The isometric knee extension (IKE) test (IKE test + strain gauge)	Buendía-Romero, 2021 [127]	ICC = 0.96–0.99 (+)	MIC not defined (?)	N/R	N/R	N/R
The Q Force	Douma, 2016 [128]	ICC = 0.80–0.96 (+)	MIC not defined (?)	N/R	N/R	N/R
An analog dynamometer (SENSIX®, Poitiers, France) coupled with the DELSYS System (Trigno sensor, DELSYS, INC Boston; MA)	Gafner, 2017 [129]	ICC = 0.90–0.94 (+)	MIC not defined (?)	N/R	N/R	N/R
The Biodex System 3 isokinetic dynamometer (Biodex Medical Systems, Shirley, NY)	Hartmann, 2009 [130] Symons, 2004 [131]	ICC ≥ 70 (+)	MIC not defined (?)	N/R	N/R	N/R
The Isokinetic dynamometer (KinCom 500H, Chattecx Corp., Hixson, TN, USA)	Holsgaard Larsen, 2007 [108]	ICC not reported (?)	N/R	N/R	N/R	N/R
The Leg Press Sled (LPS)	Hutchison, 2006 [132]	ICC ≥ 0.70 (+)	MIC not defined (?)	N/R	r ≥ 0.70 (+)	N/R
The Microfet 2000 strain gauge portable dynamometer (PD)	Karner, 1998 [133]	ICC ≥ 0.70 (+)	N/R	N/R	N/R	N/R
A load cell setup	Keshavarzi, 2022 [134]	ICC = 0.99 (+)	MIC not defined (?)	N/R	N/R	N/R
The push-off test (POT)	Legg, 2020 [135]	ICC = 0.92 (+)	MIC not defined (?)	N/R	N/R	r > 0.50 (+)
The functional multi-joint isokinetic dynamometer	Legg, 2020 [135]	ICC = 0.98 (+)	MIC not defined (?)	N/R	N/R	r > 0.50 (+)
The MyBalance test	Mansson, 2021 [73]	N/R	N/R	N/R	N/R	r not reported (?)
The maximal isometric strength test of the trunk (measured by a precalibrated digital loading cell connected to the MuscleLab software)	Mesquita, 2019 [136]	ICC ≥ 0.70 (+)	MIC not defined (?)	N/R	N/R	N/R
The one-repetition maximum (1 RM) using elastic resistance bands test	Nyberg, 2014 [137]	N/R	N/R	N/R	Correlation >0.70 (+)	N/R
The lateral step (LS) test	Porto, 2020 [138]	ICC = 0.95 (+)	N/R	N/R	r < 0.70 (−)	Hypothesis confirmed (+)
Tandem Gait (TG)	Porto, 2020 [138]	ICC ≥ 0.70 (+)	N/R	N/R	r < 0.70 (−)	Hypothesis confirmed (+)
Single-leg stance (SS) test	Porto, 2020 [138]	N/R	N/R	N/R	r < 0.70 (−)	Hypothesis not confirmed (−)
The one repetition maximum (1 RM) using a muscle strength training device for the arm/shoulder (Pull Down, Norway)	Rydwik, 2007 [139]	ICC not reported (?)	MIC not defined (?)	N/R	N/R	Hypothesis not confirmed (−)
The five-repetition sit-to-stand (STS) test	Schaubert, 2005 [140]	ICC = 0.82 (+)	MIC not defined (?)	N/R	N/R	N/R
A standardised heel-rise test (Using trunk accelerometry)	Schmid, 2011 [112]	ICC = 0.31 and 0.79 (+)	MIC not defined (?)	N/R	N/R	r > 0.50 (+)
The one-repetition maximum (1 RM) performed on the Keiser A-300 pneumatic equipment (Keiser Corp., Fresno, CA) or on selectorised weight-stack resistance exercise machines (Cybex VR2; Cybex International Inc., Medway, MA)	Schroeder, 2007 [113]	ICC not reported (?)	N/R	N/R	N/R	N/R
Grip strength, measured using a Smedley-type dynamometer (T.K.K.5401, TAKEI Scientifc Instruments Co., Ltd., Niigata, Japan)	Suzuki, 2019 [141]	ICC = 0.96 (+)	MIC not defined (?)	N/R	N/R	N/R
Knee extension strength, measured using a handheld dynamometer (μ-Tas F-1; Anima Inc., Tokyo, Japan)	Suzuki, 2019 [141]	ICC = 0.90 (+)	MIC not defined (?)	N/R	N/R	N/R
The 30-s Chair-Stand Test	Jones, 1999 [142]	ICC ≥ 0.70 (+)	N/R	N/R	N/R	Hypotheses confirmed (+)

Measurement property rating: sufficient (+), insufficient (−), inconsistent (±), indeterminate (?)

MDD, #minimum detectable difference; RLOA, ratio of limits of agreement; SDD, smallest detectable difference; SPPB, Short Physical Performance Battery balance; GES, Gait Efficacy Scale; F8WT, Figure of 8 Walk Test; 6MWT, Six-Minute Walk Test; MCID, minimal clinically important difference.

#### Muscle strength

Our literature search identified 30 different tools for muscle strength assessment in older people. Reliability, measurement error and hypothesis testing for construct validity (i.e. convergent and discriminative validity) were the most reported measurement properties for these tools. None of the studies on tools for muscle strength reported data on responsiveness.

Only four of the tools for muscle strength assessment (Table 2 and Appendix 4.b) were validated by at least two studies, with evidence for sufficient criterion validity available for only one tool: The Handheld Dynamometry (HHD), Lafayette Manual Muscle Tester, Model #01163 [120, 123]. All the four tools showed sufficient test–retest or inter-rater reliability, while one of them (The Nintendo Wii Balance Board) [124, 125] also showed sufficient convergent validity. Among the other tools validated by only one study, evidence for sufficient criterion validity was reported for two tools: The calf-raise senior (CRS) test [119] and The Leg Press Sled (LPS) [132], which also showed sufficient reliability. A few other tools had sufficient convergent or discriminative validity, with sufficient reliability. Ultimately, the following tools showed insufficient criterion validity for muscle strength assessment in older people: The lateral step (LS) test [138], The Tandem Gait (TG) test [138] and the Single-leg stance (SS) test [138].

#### Muscle power

The 12 included studies on muscle power assessment tools evaluated 12 distinct tools (1 tool per study) (Table 3 and Appendix 4.c). Evidence for sufficient criterion validity was reported for only one tool, the 30-s sit-to-stand (STS) muscle power test [103], while another tool, the sit-to-stand (STS) performance power using a linear encoder [110] showed insufficient criterion validity. The following tools showed sufficient convergent or discriminant validity, along with sufficient reliability: The sit-to-stand power test (STSp) using a portable linear transducer [105], the chair stand mean power (CSMP) test using the Fitro Dyne device [109] and a standardised heel-rise test (using trunk accelerometry) [112]. Responsiveness was not reported by studies on muscle power assessment tools.

**Table 3:** Measurement properties of muscle power assessment tools: Summary results with overall quality ratings

**Instrument**	**Reference**	**Reliability**			**Validity**	
		***Reliability***	***Measurement error***	***Internal consistency***	***Criterion validity***	***Hypothesis testing for construct validity***
The 30-s sit-to-stand (STS) muscle power test	Alcazar, 2020 [103]	N/R	N/R	N/R	r ≥ 0.70 (+)	N/R
The sit-to-stand (STS) muscle power test	Alcazar, 2018 [104]	N/R	N/R	N/R	N/R	r > 0.50 (+)
The sit-to-stand power test (STSp), using a portable linear transducer	Balachandran, 2021 [105]	ICC = 0.96 (+)	MIC not defined (?)	N/R	N/R	Hypotheses confirmed (+)
The Vertical jump (VJ) measured by a contact mat	Farias, 2013 [106]	ICC = 0.91–0.96 (+)	MIC not defined (?)	N/R	N/R	N/R
The Tendo Weightlifting Analyser (Trencin, Slovak Republic)	Grey, 2014 [107]	ICC not reported (?)	N/R	N/R	N/R	r > 0.50 (+)
Counter-movement jump (CMJ) test performed on a force platform (Kistler Instruments 9,281 B, Winterthur, Switzerland, 40 x 60 cm)	Holsgaard Larsen, 2007 [108]	ICC not reported (?)	N/R	N/R	N/R	N/R
The chair stand mean power (CSMP) test, using the Fitro Dyne device (Fitronic S. R. O. Co, Slovakia).	Kato, 2015 [109]	ICC = 0.88–0.92 (+)	N/R	N/R	N/R	Hypothesis confirmed (+)
The sit-to-stand (STS) performance power using a linear encoder (MuscleLab Power model MLPRO, Ergotest Technology, Langesund, Norway)	Lindemann, 2015 [110]	N/R	N/R	N/R	r < 0.70 (−)	N/R
The Jumping Mechanography	Rittweger, 2004 [111]	ICC not reported (?)	MIC not defined (?)	N/R	N/R	r > 0.50 (+)
A standardised heel-rise test (Using trunk accelerometry)	Schmid, 2011 [112]	ICC = 0.78–0.80 (+)	MIC not defined (?)	N/R	N/R	r > 0.50 (+)
Unilateral leg extension power (W) using the Bassey Power Rig (University of Nottingham, Nottingham, U.K.)	Schroeder, 2007 [113]	ICC not reported (?)	N/R	N/R	N/R	N/R
The Ramp Power Test	Signorile, 2007 [114]	ICC = 0.921 (+)	MIC not defined (?)	N/R	N/R	r not reported (?)

N/R = Not reported

Measurement property rating: sufficient (+), insufficient (−), inconsistent (±), indeterminate (?)

SDC, smallest detectable change; LoA, limits of agreement

#### Endurance

The 6-Minute Walk Test is the single tool for endurance assessment, evaluated by the only one study [143]. It has sufficient test–retest reliability and sufficient convergent and discriminative validity. Neither criterion validity, nor responsiveness were reported for this tool (Table 4 and Appendix 4.d).

**Table 4:** Measurement properties of endurance assessment tools: Summary results with overall quality ratings

**Instrument**	**Reference**	**Reliability**			**Validity**	
		***Reliability***	***Measurement error***	***Internal consistency***	***Criterion validity***	***Hypothesis testing for construct validity***
The 6-Minute Walk Test	Rikli, 1998 [143]	ICC ≥ 0.70 (+)	MIC not defined (?)	N/R	N/R	Hypotheses confirmed (+)

ANOVA, analysis of variance.

Measurement property rating: sufficient (+), insufficient (−), inconsistent (±), indeterminate (?)

### Methodological quality of studies (RoB) and quality of evidence (GRADE)

Appendix 5 reports the outcomes of the risk of bias assessment for each measurement property assessed in the included studies (Appendices 5a−d). For most of the tools and studies, criterion validity, convergent validity and discriminative validity were found to have ‘very good’ methodological quality. In contrary, for reliability and measurement error, the far dominant ratings were ‘adequate’ and ‘doubtful’.

Using the outcomes of the risk of bias assessment along with other criteria (inconsistency, imprecision and indirectness) of the modified GRADE approach for grading the quality of evidence for outcome measurement instruments [15], we assessed the certainty of evidence for tools with at least two validation studies. This assessment included 15 balance tools and 4 muscle strength tools (Appendix 6). Our analyses showed high quality evidence for both sufficient validity (at minimum convergent validity) and reliability for the following tools:

- *The Balance Evaluation Systems Test (BESTest)*: Criterion validity, convergent and discriminative validity and reliability.
- *The Mini-Balance Evaluation Systems Test (Mini-BESTest)*: Criterion validity, convergent validity and reliability.
- *The Berg Balance Scale (BBS)*: Convergent and discriminative validity and reliability.
- *The Timed Up and Go (TUG) test*: Convergent and discriminative validity and reliability.

*The Brief-Balance Evaluation Systems Test (Brief-BESTest)* had high quality evidence for sufficient criterion validity and convergent validity, but the quality of evidence was downgraded to moderate for reliability, due to small sample size (<100 subjects). None of the muscle strength assessment tools had high quality evidence for both validity and reliability.

As patients were recruited from various settings, we sought to stratify the analyses by settings (e.g. community vs long-term care facility) for tools with high quality evidence for sufficient validity and reliability to check whether the findings reported above equally apply to either setting. However, there were not sufficient data to allow such analyses for all the tools. In fact, of the six studies on the BESTest, five included patients from the community. Likewise, for the Mini-BESTest and the TUG which were each assessed in five studies, four of the studies on each of these tools included subjects from the community. Analysis by setting was therefore possible only for the BBS, which was assessed in five studies that recruited patients from the community and in six studies that included patients living in long-term care facilities. This analysis showed that the results for each setting were similar to those reported when all the included studies for the BBS were considered together (data not shown).

## Discussion

This systematic review aimed to identify all the studies validating the available measurement instruments for locomotor capacity or specific attributes of locomotor capacity, as defined by the WHO Locomotor Capacity Working Group [4], and to assess the methodological quality of the studies and the measurement properties of the tools. From the 125 studies retrieved by our comprehensive literature search, we identified 69 balance assessment tools, 30 tools for muscle strength, 12 tools for muscle power and 1 endurance assessment tool, with varying numbers of validation studies for each tool. Balance assessment tools had the highest numbers of validation studies per tool, although the overwhelming majority of existing tools were validated by only one study (only 15 tools had at least two validation studies). For muscle power and endurance, only one validation study was retrieved for each tool. Of important note, our literature search did not retrieve any assessment tool or validation studies for muscle function or joint function. Although no tools were identified for assessment of locomotor capacity overall, this finding was not so surprising or disappointing, as locomotor capacity is a relatively new concept with process for consensus on conceptual and operational definitions started only recently [4].

The GRADE assessment of confidence in evidence on measurement properties for balance tools (considering tools having at least two validation studies) revealed that only very few of these tools have high quality evidence for both sufficient validity and reliability. In fact, high-quality evidence for both sufficient criterion and construct validity and for reliability was found for only two tools: The Balance Evaluation Systems Test (BESTest) and the Mini-Balance Evaluation Systems Test (Mini-BESTest) [20]. However, this evidence applies more to community-dwelling patients, as only one study on the BESTest (on six studies included) and one on the Mini-BESTest (on five studies included) recruited patients from long-term care facilities. Therefore, whether this evidence may apply as well to patients living in long-term care facilities remains to be confirmed. The Berg Balance Scale (BBS) [27] showed high-quality evidence for sufficient construct validity (convergent and discriminative validity), and for reliability, as had the TUG test [41]. However, contrary to the TUG for which this evidence applies more to community-dwelling patients (only one study of five on TUG recruited patients from a nursing home), the evidence on the measurement properties of the BBS applies equally to both community-dwelling patients and long-term care facility residents. Consistent with our findings on balance assessment tools, an expert panel acknowledged the excessive number of standing balance assessment tools and reached consensus on two balance measures, recommending that at a minimum, either the BBS or the Mini-BESTest be used for measuring standing balance in adult populations [144]. Our analyses showed that none of the tools for muscle strength assessment has high quality evidence for both sufficient validity and reliability.

Regarding endurance, the only tool identified by our systematic review is the 6-Minute Walk Test [143]. Although it has good convergent validity, discriminative validity and reliability (when applying the criteria for good measurement properties), further validation studies are needed to strengthen the evidence on the usefulness of this tool in older people. Many other tools already exist for walking endurance assessment, which have been validated or used in other age groups and populations. These include the endurance shuttle walk test [145], which was validated for the assessment of endurance capacity in patients with chronic obstructive pulmonary disease (COPD), the long distance corridor walk [146] and the 400-m Walk Test [147]. These tools may also be validated for use in healthy older people in community and long-term care facilities.

This systematic review did not identify any tool formally validated as a measure of joint function in older people. However, the goniometer, which seems to have been used in clinical practice as ‘a proxy’ for joint function assessment may be a useful tool in assessing locomotor capacity. In fact, in clinical research, this tool has rather been used to assess range of motion [148], even if there seems to be a confusion between range of motion and joint function in some publications [149, 150].

In order to come to clear conceptual and operational definitions of locomotor capacity in older people, there are some burning issues that the WHO Locomotor Capacity Working Group [4] may need to further discuss, including the usefulness of considering muscle function as an attribute of locomotor capacity, along with muscle strength and power.

### Issues to be addressed by the WHO locomotor capacity working group

The findings of this systematic review reveal that the WHO Locomotor Capacity Working Group still have to clarify several aspects related to the current attributes of locomotor capacity. First, regarding balance, it is important to clarify whether static or dynamic balance are to be assessed, or both, even if most of the main tools included in this systematic review assess both aspects of balance [20]. It may also be important to clarify whether only standing balance is to be assessed in the context of locomotor capacity, or whether sitting balance [151] is also essential. Regarding this particular aspect, we assumed in this systematic review that standing balance was the type of balance to be considered in the context of assessment of locomotor capacity; therefore, studies assessing tools for sitting balance were excluded as ‘not locomotor capacity or attributes’. Second, regarding muscle strength, it may be important to clarify which specific muscle groups are to be primarily assessed, as various identified tools target various muscle groups [120, 126, 132, 134]. For example, whether handgrip strength measures should be considered in the context of locomotor capacity assessment is to be clarified, even if grip strength has been found to reflect general muscle strength [152, 153]. In fact, handgrip strength has also been identified as a measure of vitality, one of the six key domains of the WHO intrinsic capacity concept [154]; in addition, the fact that grip strength can represent global muscle strength should not eliminate the need to assess specific muscle groups, when indicated [155]. Third, when referring to endurance, it may be useful to precise that we are talking about ‘walking endurance’, and not about ‘muscle endurance’ [156]. In this systematic review, we assumed that only ‘walking endurance’ had to be considered and therefore, we did not include studies assessing the measurement properties of tools for ‘muscle endurance’. Furthermore, it is worth noting that ‘muscle endurance’ has yet been considered as one of the measures of vitality capacity in the WHO working definition of this other key domain of intrinsic capacity [157]. Forth, regarding joint function, which seems to have been assessed in practice through range of motion, one might wonder why range of motion itself would not be directly listed as an attribute of locomotor capacity, instead of joint function. Fifth, beyond the fact that our systematic literature search identified no measurement tools for muscle function, it may be important to further discuss the usefulness of considering muscle function as an attribute of locomotor capacity, knowing that muscle function has been defined as including measures of strength and power [158]. In the end (sixth), another important issue that the WHO Locomotor Capacity Working Group will need to address is to provide consensus definitions of terms used to define locomotor capacity (i.e. the attributes). These definitions may be provided in a consensus paper summarising terms commonly used to define intrinsic capacity (taking the form of a glossary), including terms used to define the other domains of intrinsic capacity.

### Limitations of the study

We acknowledge some limitations of this systematic review. First, we limited our literature search to articles published in English, which may have excluded some validation studies published in other languages. However, research has reported that excluding non-English language publications from evidence-syntheses did not jeopardise the conclusions of systematic reviews [159]. Besides the issue of language restriction, our search strategies may have not captured a few validation studies from the databases searched, as in any systematic review, mainly for the attributes for which MeSH or Emtree terms are not yet available (i.e. muscle function, joint function and muscle power). However, the search strategies were detailed enough, and our literature search covered the most important and relevant databases (including Scopus that doesn’t use thesaurus terms), so that we can be quite confident that we didn’t miss any significant evidence that would alter the conclusions of this research.

### Implications for future research

One important question raised by the findings of this systematic review is: Why has all this research been conducted on so many tools if, at the end, the studies bring limited evidence on the usefulness of these tools for the intended purpose? Considering this, several strong recommendations are to be formulated:

First, future validation studies should adhere to the COSMIN terminology of measurement properties [16] and to the COSMIN reporting guideline for primary studies on measurement tools [160]. Second, researchers should avoid fragmented research questions (i.e. validation studies addressing only single specific aspects of measurement properties) and consider instead thoroughly assessing all the relevant measurement properties and aspects for each single tool, with adequate sample size. Third, the findings of this systematic review underling that future research agenda should focus on development and validation of tools to measure other attributes of locomotor capacity, for which high quality evidence for validity, reliability and responsiveness is lacking in older people. These include endurance, for which tools already exist with evidence for validity and reliability in other populations or age groups [147]. Regarding balance and specifically standing balance, we think there is no need to invest in the development of new assessment tools, given the excessive number of existing tools. Instead, researchers should focus on setting up well designed studies to provide high quality evidence on the measurement properties (i.e. complete evidence with regard to validity, reliability and responsiveness) of some of the most promising existing tools, with a particular attention to feasibility aspects (e.g. completion time, ease of administration, required equipment, etc.). Fourth, future research should also consider validation of these tools in low- and middle-income countries, particularly in African countries, and in other high-income countries where these tools are not yet validated. Fifth, research should be initiated to provide Minimal Important Change (MIC) values for available tools in older people, as missing MIC values hampered the rating of measurement error in almost all the included studies. Sixth, as a final but not least strong recommendation for researchers, future validation studies of tools for locomotor capacity should include in a single study two subsets of sample, one including patients recruited from the community and another one formed with patients recruited from long-term care facilities. By so doing, each single study will provide at the same time, evidence on the appropriateness of the tools for patients residing in both settings.

In support to all these recommendations, we would like to remind to all researchers this important message from Doug Altman (of revered memory) in his Editorial titled ‘The scandal of poor medical research’: ‘*We need less research, better research, and research done for the right reasons*’ [161]. We hope that lessons learned from this systematic review and outlined here will serve future researchers in designing, conducting and reporting their research on validation of tools to assess locomotor capacity. As research needs in this setting are urgent, beyond hopes, we strongly call researchers for high quality research to provide WHO, countries and clinicians with effective tools to measure locomotor capacity, by fully complying with the COSMIN terminologies and recommendations [16, 160] and by following good research practice principles [162]. In fact, ultimately, this will contribute to the wellbeing of our older people, by helping meet the United Nations decade of healthy ageing goals [5].

## Conclusion

Without strong evidence supporting the validity and reliability of measurement instruments, the choice of adequate tools to screen and monitor health status of older people may be a hazardous travel. To the best of our knowledge, this systematic review is the first that assessed the measurement properties of tools to measure all the attributes of locomotor capacity. The outcomes of this study will first support the WHO Locomotor Capacity Working Group in the process of developing both conceptual and operational definitions of locomotor capacity [4]. Ultimately, these findings will help WHO in providing evidence-based recommendations for adequate tools to be used in clinical and population settings to assess locomotor capacity, and thereby, will contribute to adequate monitoring of healthy ageing and actions taken by WHO and the United Nations in the context of the 2021–2030 Decade of Healthy Ageing initiative [5, 6]. In the absence of strong evidence for validity and reliability of tools for most of the attributes of locomotor capacity in older people, WHO may provide interim recommendations for specific tools, following paradigms for appropriately formulated discordant recommendations [163].

## Supplementary Material

aa-23-0360-File002_afad139

aa-23-0360-File003_afad139

aa-23-0360-File004_afad139

aa-23-0360-File005_afad139

aa-23-0360-File006_afad139

aa-23-0360-File007_afad139

## Data Availability

All the data that support the findings and conclusions of this study are available as Appendices to this manuscript.
