# Supplementary material for: Measures of attributes of locomotor capacity in older people: a systematic literature review following the COSMIN methodology
Source: Age Ageing. 2023 Oct 30;52(Suppl 4):iv44–66. doi: 10.1093/ageing/afad139 (PMC10615073; doi:10.1093/ageing/afad139)
Supplement: aa-23-0360-File002_afad139 [file aa-23-0360-file002_afad139.doc]

World Health Organisation: *Measurements of Healthy Ageing*

**Measures of attributes of locomotor capacity in older people: A systematic literature review following the COSMIN methodology**

**SUPPLEMENTARY DATA**

**Appendix 1:** Search strategies

Database: **Ovid MEDLINE(R) ALL**

Search Strategy:

--------------------------------------------------------------------------------

1 exp Aged/

2 (elderly or centenarian* or nonagenarian* or octogenarian* or "oldest old" or "older patient*" or "older people" or "older person*" or "older adult*" or "older men" or "older women" or "older resident*" or "older individual*" or "older healthy adult*" or "older active adult*" or "older healthy individual*" or "older active individual*" or "older active men" or "older active women" or "older healthy men" or "older healthy women").ti,ab,kw.

3 ((old or aged or "super-aged") adj (patient* or people or group* or person* or resident* or adult* or individual* or men or women)).ti,ab,kw.

4 ((ageing or aging) adj (patient* or people or group* or person* or resident* or adult* or individual* or men or women)).ti,ab,kw.

5 Middle Aged/

6 "Middle Aged".ti,ab,kw.

7 (senior* or elder*1).ti,ab,kw.

8 1 or 2 or 3 or 4 or 5 or 6 or 7

9 (Geriatric Assessment/ or Self-Assessment/ or Patient Outcome Assessment/) and (tool* or questionnaire* or test* or scale* or instrument* or index or indice*).ti,ab,kw.

10 ((screening* or measurement* or assessment* or estimation* or evaluation* or clinical or appraisal* or rating*) adj2 (tool* or questionnaire* or test* or scale* or instrument* or index or indice*)).ti,ab,kw.

11 (("HR-PRO" or HRPRO or HRQL or HRQoL or QL or QoL).ti,ab. or "quality of life".mp. or ("health index*" or "health indices" or "health profile*").ti,ab. or "health status".mp. or ((patient or self or child or parent or carer or proxy) adj (appraisal* or appraised or report or reported or reporting or rated or rating* or based or assessed or assessment*)).ti,ab.) adj2 (tool* or questionnaire* or test* or scale* or instrument* or index or indice*).ti,ab,kw.

12 9 or 10 or 11

13 Physical Endurance/

14 Endurance.ti,ab,kw.

15 Postural Balance/

16 (balance or "posture equilibrium*" or "postural equilibrium" or "musculoskeletal equilibrium" or "postural control*" or "posture control*" or "postural stability").ti,ab,kw.

17 Muscle Strength/

18 ("arthrogenic muscle inhibition" or "muscle strength").ti,ab,kw.

19 ("muscle function" or "muscle power" or "joint function").ti,ab,kw.

20 Locomotion/

21 ("locomotor capacity" or "locomotor activit*" or locomotion).ti,ab,kw.

22 13 or 14 or 15 or 16 or 17 or 18 or 19 or 20 or 21

23 8 and 12 and 22

24 23 not ("addresses" or "biography" or "case reports" or "comment" or "directory" or "editorial" or "festschrift" or "interview" or "lectures" or "legal cases" or "legislation" or "letter" or "news" or "newspaper article" or "patient education handout" or "popular works" or "congresses" or "consensus development conference" or "consensus development conference, nih" or "practice guideline").pt. not (animals/ not humans/)

***************************

Database: **Scopus**

Search Strategy:

--------------------------------------------------------------------------------

1 TITLE-ABS-KEY ( elderly OR centenarian* OR nonagenarian* OR octogenarian* OR "oldest old" OR "older patient*" OR "older people" OR "older person*" OR "older adult*" OR "older men" OR "older women" OR "older resident*" OR "older individual*" OR "older healthy adult*" OR "older active adult*" OR "older healthy individual*" OR "older active individual*" OR "older active men" OR "older active women" OR "older healthy men" OR "older healthy women" )

2 TITLE-ABS-KEY((old OR aged OR "super-aged") W/0 (patient* OR people OR group* OR person* OR resident* OR adult* OR individual* OR men OR women))

3 TITLE-ABS-KEY((ageing OR aging) W/0 (patient* OR people OR group* OR person* OR resident* OR adult* OR individual* OR men OR women))

4 TITLE-ABS-KEY ( "Middle Aged" OR senior* OR elder)

**5 #1 OR #2 OR #3 OR #4**

6 TITLE-ABS-KEY (( "Geriatric Assessment" OR "Self-Assessment" OR "Patient Outcome Assessment" ) W/2 ( tool* OR questionnaire* OR test* OR scale* OR instrument* OR index OR indice* ))

7 TITLE-ABS-KEY ( ( screening* OR measurement* OR assessment* OR estimation* OR evaluation* OR clinical OR appraisal* OR rating* ) W/2 ( tool* OR questionnaire* OR test* OR scale* OR instrument* OR index OR indice* ) )

8 (TITLE-ABS ( "HR-PRO" OR hrpro OR hrql OR hrqol OR ql OR qol ) OR ALL ( "quality of life" ) OR TITLE-ABS ( "health index*" OR "health indices" OR "health profile*" ) OR ALL ( "health status" ) OR TITLE-ABS ( ( patient OR self OR child OR parent OR carer OR proxy ) W/0 ( appraisal* OR appraised OR report OR reported OR reporting OR rated OR rating* OR based OR assessed OR assessment* ) ) ) W/2 TITLE-ABS-KEY ( tool* OR questionnaire* OR test* OR scale* OR instrument* OR index OR indice* )

**9 #6 OR #7 OR #8**

10 TITLE-ABS-KEY ( endurance OR balance OR "posture equilibrium*" OR "postural equilibrium" OR "musculoskeletal equilibrium" OR "postural control*" OR "posture control*" OR "postural stability" )

11 TITLE-ABS-KEY ( "arthrogenic muscle inhibition" OR "muscle strength" OR "muscle function" OR "muscle power" OR "joint function" OR "locomotor capacity" OR "locomotor activit*" OR locomotion )

**12 #10 OR #11**

**13 #5 AND #9 AND #12**

14 AND ( LIMIT-TO ( DOCTYPE , "ar" ) ) AND ( LIMIT-TO ( EXACTKEYWORD , "Human" ) OR LIMIT-TO ( EXACTKEYWORD , "Humans" ) ) AND ( LIMIT-TO ( LANGUAGE , "English" ))

**Scopus (Combined version)**

( ( TITLE-ABS-KEY ( elderly OR centenarian* OR nonagenarian* OR octogenarian* OR "oldest old" OR "older patient*" OR "older people" OR "older person*" OR "older adult*" OR "older men" OR "older women" OR "older resident*" OR "older individual*" OR "older healthy adult*" OR "older active adult*" OR "older healthy individual*" OR "older active individual*" OR "older active men" OR "older active women" OR "older healthy men" OR "older healthy women" ) ) OR ( TITLE-ABS-KEY ( ( old OR aged OR "super-aged" ) W/0 ( patient* OR people OR group* OR person* OR resident* OR adult* OR individual* OR men OR women ) ) ) OR ( TITLE-ABS-KEY ( ( ageing OR aging ) W/0 ( patient* OR people OR group* OR person* OR resident* OR adult* OR individual* OR men OR women ) ) ) OR ( TITLE-ABS-KEY ( "Middle Aged" OR senior* OR elder ) ) ) AND ( ( TITLE-ABS-KEY ( ( "Geriatric Assessment" OR "Self-Assessment" OR "Patient Outcome Assessment" ) W/2 ( tool* OR questionnaire* OR test* OR scale* OR instrument* OR index OR indice* ) ) ) OR ( TITLE-ABS-KEY ( ( screening* OR measurement* OR assessment* OR estimation* OR evaluation* OR clinical OR appraisal* OR rating* ) W/2 ( tool* OR questionnaire* OR test* OR scale* OR instrument* OR index OR indice* ) ) ) OR ( ( TITLE-ABS ( "HR-PRO" OR hrpro OR hrql OR hrqol OR ql OR qol ) OR ALL ( "quality of life" ) OR TITLE-ABS ( "health index*" OR "health indices" OR "health profile*" ) OR ALL ( "health status" ) OR TITLE-ABS ( ( patient OR self OR child OR parent OR carer OR proxy ) W/0 ( appraisal* OR appraised OR report OR reported OR reporting OR rated OR rating* OR based OR assessed OR assessment* ) ) ) W/2 TITLE-ABS-KEY ( tool* OR questionnaire* OR test* OR scale* OR instrument* OR index OR indice* ) ) ) AND ( ( TITLE-ABS-KEY ( endurance OR balance OR "posture equilibrium*" OR "postural equilibrium" OR "musculoskeletal equilibrium" OR "postural control*" OR "posture control*" OR "postural stability" ) ) OR ( TITLE-ABS-KEY ( "arthrogenic muscle inhibition" OR "muscle strength" OR "muscle function" OR "muscle power" OR "joint function" OR "locomotor capacity" OR "locomotor activit*" OR locomotion ) ) ) AND ( LIMIT-TO ( DOCTYPE , "ar" ) ) AND ( LIMIT-TO ( EXACTKEYWORD , "Human" ) OR LIMIT-TO ( EXACTKEYWORD , "Humans" ) ) AND ( LIMIT-TO ( LANGUAGE , "English" ) )

Database: **Embase**

Search Strategy:

--------------------------------------------------------------------------------

1 'Aged'/exp

2 (elderly OR centenarian* OR nonagenarian* OR octogenarian* OR "oldest old" OR "older patient*" OR "older people" OR "older person*" OR "older adult*" OR "older men" OR "older women" OR "older resident*" OR "older individual*" OR "older healthy adult*" OR "older active adult*" OR "older healthy individual*" OR "older active individual*" OR "older active men" OR "older active women" OR "older healthy men" OR "older healthy women"):ti,ab,kw

3 ((old OR aged OR "super-aged") NEAR/1 (patient* OR people OR group* OR person* OR resident* OR adult* OR individual* OR men OR women)):ti,ab,kw

4 ((ageing OR aging) NEAR/1 (patient* OR people OR group* OR person* OR resident* OR adult* OR individual* OR men OR women)):ti,ab,kw

5 'middle aged'/de

6 "middle aged":ti,ab,kw

7 (senior* OR elder$):ti,ab,kw

**8 #1 OR #2 OR #3 OR #4 OR #5 OR #6 OR #7**

9 ('geriatric assessment' OR 'self evaluation' OR 'outcome assessment')/de AND (tool* OR questionnaire* OR test* OR scale* OR instrument* OR index OR indice*):ti,ab,kw

10 ((screening* OR measurement* OR assessment* OR estimation* OR evaluation* OR clinical OR appraisal* OR rating*) NEAR/3 (tool* OR questionnaire* OR test* OR scale* OR instrument* OR index OR indice*)):ti,ab,kw

11 (("HR-PRO" or HRPRO or HRQL or HRQoL or QL or QoL or "quality of life" or "health index*" or "health indices" or "health profile*" or "health status") NEAR/3 (tool* or questionnaire* or test* or scale* or instrument* or index or indice*)) OR ((patient OR self OR child OR parent OR carer OR proxy) NEAR/1 (appraisal* OR appraised OR report OR reported OR reporting OR rated OR rating* OR based OR assessed OR assessment*) NEAR/3 (tool* OR questionnaire* OR test* OR scale* OR instrument* OR index OR indice*))

**12 #9 or #10 or #11**

13 'endurance'/de

14 "endurance":ti,ab,kw

15 'body equilibrium'/de

16 (balance OR "posture equilibrium*" OR "postural equilibrium" OR "musculoskeletal equilibrium" OR "postural control*" OR "posture control*" OR "postural stability"):ti,ab,kw

17 'muscle strength'/de

18 ("arthrogenic muscle inhibition" OR "muscle strength"):ti,ab,kw

19 ('muscle function' OR 'joint function')/de OR ("muscle function" or "muscle power" or "joint function"):ti,ab,kw

20 'locomotion'/de

21 ("locomotor capacity" OR "locomotor activit*" OR locomotion):ti,ab,kw

**22 #13 OR #14 OR #15 OR #16 OR #17 OR #18 OR #19 OR #20 OR #21**

**23 #8 AND #12 AND #22**

24 #23 AND ('article'/it OR 'article in press'/it)

Database: **Cumulative Index to Nursing and Allied Health Literature : CINAHL**

Search Strategy:

--------------------------------------------------------------------------------

1 (MH "Aged+")

2 AB (elderly OR centenarian* OR nonagenarian* OR octogenarian* OR "oldest old" OR "older patient*" OR "older people" OR "older person*" OR "older adult*" OR "older men" OR "older women" OR "older resident*" OR "older individual*" OR "older healthy adult*" OR "older active adult*" OR "older healthy individual*" OR "older active individual*" OR "older active men" OR "older active women" OR "older healthy men" OR "older healthy women")

3  AB ((old OR aged OR "super-aged") N0 (patient* OR people OR group* OR person* OR resident* OR adult* OR individual* OR men OR women))

4  AB ((ageing OR aging) N0 (patient* OR people OR group* OR person* OR resident* OR adult* OR individual* OR men OR women))

5  (MH "Middle Age")

6  AB ("Middle Aged")

7  AB (senior* OR elder#)

8 S1 OR S2 OR S3 OR S4 OR S5 OR S6 OR S7

9  ((MH "Geriatric Assessment") OR (MH "Self Assessment") OR (MH "Outcome Assessment")) AND AB (tool* OR questionnaire* OR test* OR scale* OR instrument* OR index OR indice*)

10  AB ( ( screening* OR measurement* OR assessment* OR estimation* OR evaluation* OR clinical OR appraisal* OR rating* ) N2 ( tool* OR questionnaire* OR test* OR scale* OR instrument* OR index OR indice* ) )

11 ( ( AB ( "HR-PRO" OR hrpro OR hrql OR hrqol OR ql OR qol ) OR TX ( "quality of life" ) OR AB ( "health index*" OR "health indices" OR "health profile*" ) OR TX ( "health status" ) OR AB ( ( patient OR self OR child OR parent OR carer OR proxy ) N0 ( appraisal* OR appraised OR report OR reported OR reporting OR rated OR rating* OR based OR assessed OR assessment* ) ) ) N2 (AB ( tool* OR questionnaire* OR test* OR scale* OR instrument* OR index OR indice* )))

12  S9 OR S10 OR S11

13  (MH "Physical Endurance")

14  AB (Endurance)

15  (MH "Balance, Postural")

16 AB ( endurance OR balance OR "posture equilibrium*" OR "postural equilibrium" OR "musculoskeletal equilibrium" OR "postural control*" OR "posture control*" OR "postural stability" )

17 (MH "Muscle Strength")

18 AB ("arthrogenic muscle inhibition" OR "muscle strength")

19  AB ("muscle function" OR "muscle power" OR "joint function")

20 (MH "Locomotion+")

21 AB ("locomotor capacity" OR "locomotor activit*" OR locomotion)

22  S13 OR S14 OR S15 OR S16 OR S17 OR S18 OR S19 OR S20 OR S21

23  S8 AND S12 AND S22

24 Narrow by Language: - english; Narrow by Source Types : - Academic Journals

Database: **APA PsycInfo**

Search Strategy:

--------------------------------------------------------------------------------

1 Older Adulthood/

2 (elderly or centenarian* or nonagenarian* or octogenarian* or "oldest old" or "older patient*" or "older people" or "older person*" or "older adult*" or "older men" or "older women" or "older resident*" or "older individual*" or "older healthy adult*" or "older active adult*" or "older healthy individual*" or "older active individual*" or "older active men" or "older active women" or "older healthy men" or "older healthy women").ti,ab,id.

3 ((old or aged or "super-aged") adj1 (patient* or people or group* or person* or resident* or adult* or individual* or men or women)).ti,ab,id.

4 ((ageing or aging) adj1 (patient* or people or group* or person* or resident* or adult* or individual* or men or women)).ti,ab,id.

5 Middle Adulthood/

6 "Middle Aged".ti,ab,id.

7 (senior* or elder*1).ti,ab,id.

8 1 or 2 or 3 or 4 or 5 or 6 or 7

9 (geriatric assessment/ or self-evaluation/ or evaluation/ or measurement/) and (tool* or questionnaire* or test* or scale* or instrument* or index or indice*).ti,ab,id.

10 ((screening* or measurement* or assessment* or estimation* or evaluation* or clinical or appraisal* or rating*) adj3 (tool* or questionnaire* or test* or scale* or instrument* or index or indice*)).ti,ab,id.

11 (("HR-PRO" or HRPRO or HRQL or HRQoL or QL or QoL).ti,ab. or "quality of life".af. or ("health index*" or "health indices" or "health profile*").ti,ab. or "health status".af. or ((patient or self or child or parent or carer or proxy) adj1 (appraisal* or appraised or report or reported or reporting or rated or rating* or based or assessed or assessment*)).ti,ab.) adj3 (tool* or questionnaire* or test* or scale* or instrument* or index or indice*).ti,ab,id.

12 9 or 10 or 11

13 exp endurance/

14 endurance.ti,ab,id.

15 equilibrium/

16 (balance or "posture equilibrium*" or "postural equilibrium" or "musculoskeletal equilibrium" or "postural control*" or "posture control*" or "postural stability").ti,ab,id.

17 physical strength/

18 ("arthrogenic muscle inhibition" or "muscle strength").ti,ab,id.

19 ("muscle function" or "muscle power" or "joint function").ti,ab,id.

20 locomotion/

21 ("locomotor capacity" or "locomotor activit*" or locomotion).ti,ab,id.

22 13 or 14 or 15 or 16 or 17 or 18 or 19 or 20 or 21

23 8 and 12 and 22

***************************
