# Supplementary material for: Measures of attributes of locomotor capacity in older people: a systematic literature review following the COSMIN methodology
Source: Age Ageing. 2023 Oct 30;52(Suppl 4):iv44–66. doi: 10.1093/ageing/afad139 (PMC10615073; doi:10.1093/ageing/afad139)
Supplement: aa-23-0360-File003_afad139 [file aa-23-0360-file003_afad139.docx]

World Health Organisation: *Measurements of Healthy Ageing*

**Measures of attributes of locomotor capacity in older people: A systematic literature review following the COSMIN methodology**

**SUPPLEMENTARY DATA**

# Appendix 2: Characteristics of the included studies

- Appendix 2.a: Characteristics of the included studies on balance assessment tools
- Appendix 2.b: Characteristics of the included studies on muscle strength assessment tools
- Appendix 2.c: Characteristics of the included studies on muscle power assessment tools
- Appendix 2.d: Characteristics of the included studies on endurance assessment tools

**Appendix 2.a**: Characteristics of the included studies on balance assessment tools

| **Instrument** | **Study Reference** | **Type of study** (As stated in the objectives) | **Study design** | **N** | **Age, years**  Mean ± SD | **% Female** | **Inclusion criteria** | **Setting** | **Country** |
| --- | --- | --- | --- | --- | --- | --- | --- | --- | --- |
| The Balance Evaluation Systems Test  (BESTest) | Anson, 2019  ***Note***: 1 other tool assessed, Mini-Best | Validation  (test stability over time, test-retest reliability, MDC, and ability to identify past fallers) | A pilot feasibility study | 58 | 78.1 ± 7.01 | 72.00 | Adults older than 65 years with self-reported balance problems without neurological or musculoskeletal diseases who scored at least 24 on the Mini-Mental State Examination and were able to walk continuously on a treadmill for 2 minutes. | Community | USA |
|  | Marques, 2016  ***Note***: 3 other tools assessed, Mini-Best, Brief-Best, and BBS | Validation (reliability, validity, and ability to identify fall status) | Cross-sectional | 122 | 75.9 ± 8.9 | 70.50 | (1) aged ≥60 years; (2) living independently in the community; and (3) able to ambulate with or without a walking aid (but without the assistance of another person). | Community | Portugal |
|  | O'Hoski, 2015  ***Note***: 2 other tools assessed, mini-BESTest and Brief-BESTest) | Validation (convergent  validity; discriminative ability) | Cross-sectional study (Secondary analysis) | 79 | 68.7 ± 10.6 | Not reported | (1) Age between 50 and 89 years, (2) residing independently in the community, (3) able to understand and follow three-step instructions, (4) able to walk 6 m independently (without a gait aid). | Community | Authors’ county: Canada |
|  | Viveiro, 2019  ***Note***: 3 other tools assessed: BBS; Mini-BESTest; Brief-BESTest. | Validation (reliability, validity, and ability to identify  fall status) | Cross-sectional study | 49 | 77.8 ± 7.2 | 61.20 | (1) 60 years or older; (2) both sexes; (3) ability to stand with or without the aid of a support device; (4) residence in the nursing home; and (5) provision of written informed consent. | Nursing home | Brazil |
|  | Wang-Hsu, 2018 | Validation (inter-rater and test-retest reliability; MDC) | A prospective cohort methodological design | 70 | 85.0 ± 5.5 | 61.40 | (1) apparently healthy, (2) 65 years or older, (3) community-dwelling, and (4) able to walk with or without assistive device independently for 100 ft. | Community-dwelling (senior independent living community) | USA |
|  | Yingyongyudha, 2016  ***Note***: 3 other tools assessed: BBS; TUG; Mini-BESTest | Validation (identifying  older adult with history of falls, based on balance scores) | Not reported | 200 | Without  History of Falls: 70.2 ± 6.8  With  History of Falls: 70.3 ± 7.3 | Total: 63.00 | (1) 60 years of age or older; (2) ability to walk without using walking aids; (3) independence in basic daily activities; (4) no history of neurological diseases such as Parkinson's disease or stroke; and (5) no severe knee pains that affected walking on the day of the assessment. | Community | Thailand |
| The ***Spanish version*** of the BESTest | Dominguez-Olivan, 2020  ***Note***: 1 other tool assessed: Spanish Mini-BESTest | Translation, Adaptation, and Validation (Internal consistency reliability; Inter-rater reliability; Validity) | A cross-sectional transcultural adaptation and validation  study | 30 | 73.3 ± 6.2 | 53.33 | (1) No present health problems relating to balance disturbances; (2) no history of falling in the last three months; (3) the ability to walk 6 m without orthopaedic devices or the assistance from another person; (4) cognitively able to receive three verbal instructions; (5) capable to perform tests without excessive fatigue. | Community | Spain |
| The Mini-Balance Evaluation Systems Test  (Mini-BESTest) | Anson, 2019  ***Note***: 1 other tool assessed, the BESTest | Validation  (test stability over time, test-retest reliability, MDC, and ability to identify past fallers) | A pilot feasibility study | 58 | 78.1 ± 7.0 | 72.00 | Adults older than 65 years with self-reported balance problems without neurological or musculoskeletal diseases who scored at least 24 on the Mini-Mental State Examination and were able to walk continuously on a treadmill for 2 minutes. | Community | USA |
|  | Marques, 2016  ***Note***: 3 other tools assessed, Best, Brief-Best, and BBS | Validation (reliability, validity, and ability to identify fall status) | Cross-sectional. | 122 | 75.9 ± 8.9 | 70.5 | (1) Aged ≥60 years; (2) living independently in the community; and (3) able to ambulate with or without a walking aid (but without the assistance of another person). | Community | Portugal |
|  | O'Hoski, 2015  ***Note***: 2 other tools assessed, BESTest and Brief-BESTest) | Validation (convergent  Validity; discriminative ability) | Cross-sectional study (Secondary analysis). | 79 | 68.7 ± 10.6 | Not reported | (1) Age between 50 and 89 years, (2) residing independently in the community, (3) able to understand and follow three-step instructions, (4) able to walk 6 m independently (without a gait aid). | Community | Authors’ county: Canada |
|  | Viveiro, 2019  ***Note***: 3 other tools assessed, BBS; BESTest; Brief-BESTest. | Validation (reliability, validity, and ability to identify  fall status) | Cross-sectional study | 49 | 77.8 ± 7.2 | 61.20 | (1) 60 years or older; (2) both sexes; (3) ability to stand with or without the aid of a support device; (4) residence in the nursing home; and (5) provision of written informed consent. | Nursing home | Brazil |
|  | Yingyongyudha, 2016  ***Note***: 3 other tools assessed: BBS; TUG; BESTest | Validation (identifying  older adults with history of falls, based on balance scores) | Not reported | 200 | Without  History of Falls: 70.2 ± 6.8  With  History of Falls: 70.3 ± 7.3 | Total: 63.00 | (1) 60 years of age or older; (2) ability to walk without using walking aids; (3) independence in basic daily activities; (4) no history of neurological diseases such as Parkinson's disease or stroke; and (5) no severe knee pains that affected walking on the day of the assessment. | Community | Thailand |
| The ***Spanish version*** of the Mini-BESTest | Dominguez-Olivan, 2020  ***Note***: 1 other tool assessed: Spanish BESTest | Translation, Adaptation, and Validation (Internal consistency reliability; Inter-rater reliability; Validity) | A cross-sectional transcultural adaptation and validation  study | 30 | 73.3 ± 6.2 | 53.33 | (1) No present health problems relating to balance disturbances; (2) no history of falling in the last three months; (3) the ability to walk 6 m without orthopaedic devices or the assistance from another person; (4) cognitively able to receive three verbal instructions; (5) capable to perform tests without excessive fatigue. | Community | Spain |
| The modified Clinical test of Sensory Interaction in Balance (mCTSIB) of the Balance Platform Biodex Balance System (BBS) | Antoniadou, 2020 | Validation (reliability and validity) | Not reported | 100 | 71.8 ± 6.0 | 100.00 | Being female older than 65 years of age, and the status of community dwelling. | Community | Greece |
| The Berg Balance Scale (BBS) | Berg, 1992a  (*Title*: Clinical and laboratory measures of postural balance in an elderly population) | Validation  (Correlations among the different measures of balance; relation of  measures of balance to the subjects’ uses of aides when walking) | Not reported | 31 | 83.0 ± 6.9 | 71.00 | Subjects recruited from four residential care facilities and from acute and extended care wards of the Sunnybrook Health Science Centre in Toronto.  (No specific inclusion criteria reported) | Residential care facilities | Canada |
|  | Berg, 1992b  (*Title*: Measuring balance in the elderly: validation of an instrument) | Validation (concurrent criterion validity; predictive criterion validity; construct validity) | Not reported | 113 | 83.5 ± 5.3 | 82.30 | Individuals from the Griffith-McConnell-Residence, a home for the elderly in Montréal.  (No specific inclusion criteria reported) | Home for the elderly | Canada |
|  | Bogle Thorbahn, 1996 | Validation  (Predictive ability, sensitivity, and specificity) | Not reported | 66 | 79.2 ± 6.2 | 75.76 | Any independent-living resident in two life-care communities who volunteered was tested. | Life-care communities | Author’s county: USA |
|  | Harada, 1995  ***Note***: 1 other tool assessed: The POMA balance | Validation (Sensitivity, Specificity) | Not reported | 53 | 83.3 ± 7.7 | 87.00 | A convenience sample of elderly subjects was obtained from two licensed residential care facilities.  (No specific inclusion criteria reported) | Nursing homes | USA |
|  | Holbein-Jenny, 2005  ***Note***: 1 other tool assessed: the MDRT | Validation (test-retest reliability; inter-rater reliability; construct validity) | Not reported | 26 | 85.3 ± 4.9 | 80.77 | (1) An age of at least 65 years, (2) capability of flexing and holding the shoulder at 90° of flexion or abduction for the MDRT, and (3) ability to stand for each component of the test without the use of an assistive device. | Personal Care Home | USA |
|  | Marques, 2016  ***Note***: 3 other tools assessed, Mini-Best, Brief-Best, and Best | Validation (reliability, validity, and ability to identify fall status) | Cross-sectional study. | 122 | 75.9 ± 8.9 | 70.50 | (1) Aged ≥ 60 years; (2) living independently in the community; and (3) able to ambulate with or without a walking aid (but without the assistance of another person). | Community | Portugal |
|  | Muir, 2008 | Validation (predictive validity) | Prospective cohort study | 187 | 79.5 ± 5.8 | 35.00 | Community-dwelling older people  (No specific inclusion criteria reported) | Community-dwelling | Authors’ country: Canada |
|  | Pelicioni, 2022  ***Note***: 3 other tools assessed: FGA; DGI; and TUG. | Validation (reliability; validity)  *Remark*: Assessments conducted remotely via telehealth (ZOOM^®^) | A pilot study | 15 | Mean (range): 71.7 (64–78) | 53.33 | Having access to a mobile device (e.g., smartphone, laptop, tablet) with high-speed internet; being familiar with, or having support person familiar with, mobile technology; being able to walk 30 m with or without a walking aid and stand without support for 1 min. | Community-dwelling | New Zealand |
|  | Viveiro, 2019  ***Note***: 3 other tools assessed: BESTest; Mini-BESTest; Brief-BESTest. | Validation (reliability, validity, and ability to identify  fall status) | Cross-sectional study | 49 | 77.8 ± 7.2 | 61.20 | (1) 60 years or older; (2) both sexes; (3) ability to stand with or without the aid of a support device; (4) residence in the nursing home; and (5) provision of written informed consent. | Nursing home | Brazil |
|  | Wang, 2006 | Validation (acceptability, internal consistency reliability, interrater reliability, construct validity) | Not reported | 268 | 73.8 ± 5.2 | 44.40 | (1) Age ≥65 years; (2) living independently in the community; (3) independent in self-care activities (eating, dressing, bathing, getting in and out of bed, using the toilet); and (4) able to follow instructions in order to perform the tests required in this study. | Community-dwelling | Taiwan |
|  | Yingyongyudha, 2016  ***Note***: 3 other tools assessed: TUG; BESTest; Mini-BESTest | Validation (identifying  older adult with history of falls, based on balance scores) | Not reported | 200 | Without  History of Falls: 70.2 ± 6.8  With  History of Falls: 70.3 ± 7.3 | Total : 63.00 | (1) 60 years of age or older; (2) ability to walk without using walking aids; (3) independence in basic daily activities; (4) no history of neurological diseases such as Parkinson's disease or stroke; and (5) no severe knee pains that affected walking on the day of the assessment. | Community | Thailand |
| The ***Brazilian version*** of the Berg Balance Scale (BBS) | Miyamoto, 2004 | Translation and validation (reliability) | Not reported | 36 | mean (range): 72 (65 − 83) | 75.00 | Patients older than 65 years, selected consecutively from the Rheumatology Outpatient Clinic, Universidade Federal de São Paulo, Escola Paulista de Medicina (UNIFESP-EPM). | Community | Brasil |
| The Lateral Reach Test | Brauer, 1999 | Validation (construct validity; test–retest repeatability) | Not reported | 60 | 72.5 ± 5.4 | 100.00 | Elderly female volunteers aged over 65 years, able to stand unsupported for two minutes. | Community | Authors’ country: Australia |
| The Six-Spot Step Test | Brincks, 2021 | Validation (consistency within days and between days; construct validity on  the same day) | Cross-sectional study | 66 | 75.5 ± 6.6 | 75.80 | People aged 65 years or older with self-reported balance problems, or with a history of falls while standing or walking. | Community | Authors’ country: Denmark |
| The Functional reach (FR) test | Brooks, 2006 | Validation (Construct validity; responsiveness) | Pre-post design with measures at admission and  discharge. | 52 | 79.9 ± 7.7 | 67.00 | Ability to ambulate a few meters without physical assistance and to perform at least 1 of the physical performance measures, ability to speak and understand English, and ability to cognitively understand and follow instructions and provide informed written consent. | Inpatient geriatric rehabilitation centers | Authors’ country: Canada |
|  | Galhardas, 2020  ***Note***: 1 other tool assessed: the TUG | Validation (test-retest reliability) | A test-retest reliability study | 53 | 85.9 ± 3.9 | 77.36 | Being aged 75 years or older; living in a nursing home; and having a normal cognitive status according to the Portuguese version of the Mini-Mental State Examination. | Nursing home | Portugal |
|  | Giorgetti, 1998  ***Note***: 2 other tools assessed: TG & OLS | Validation (inter-rater reliability) | Not reported | Sample 1: 21 (Subjects without disability) | Mean (range): 73.1 (69–85) | 66.67 | Non-disabled community-dwelling older subjects. | Community | Authors’s country: USA |
|  | Lin, 2004  ***Note***: 3 other tools assessed: TUG, TB & OLS | Validation (practicality, reliability, validity, and responsiveness) | A prospective study | 1200 | Mean: 73.4 | 41.00 | People aged 65 and older. | Community-dwelling | Taiwan |
| Gait Initiation Assessment | Chang, 1999 | Validation (specificity and sensitivity) | Cross-sectional, intact-groups research design | 59 | Healthy: 73.7 ± 1.3  Disabled: 78.1 ± 1.7  With vestibular hypofunction: 74.1 ± 1.4 | Not reported | Criteria for healthy elders: age, ≥ 65 yrs, cognitively intact, no functional  limitation, no assistive device during functional activities, negative neurologic and orthopedic examination, and no medication that could influence balance. | Community-dwelling  (They were volunteers in a research project and  participated in various tests of balance) | Authors’ country: USA |
| The modified Wii Fit Balance Board  (Wii Fit® platform,  Nintendo, Japan) | Chang, 2013 | Validation (reliability and validity) | Pretest-posttest design | 20 | 67.3 ± 3.4 | Not reported | Elderly people were recruited. Participants were excluded if they had history of neurological diseases (eg, stroke, Parkinson’s disease, or cerebellar atrophy), were unable to stand, or had poor physical fitness that would have affected their abilities in the tests. | Not reported | Authors’ country: Taiwan |
| The Stepping Threshold Test (STT) | Adams, 2021 | Validation (convergent validity, discriminative validity, feasibility and interpretability) | Not reported | 70 | 74.8 ± 6.0 | 64.30 | Adults aged 65 and older. Subjects had to be able to walk for at least 20 min without a walking aid and had to be fall-prone. The latter could be met in two ways. It was identified either the subject has experienced a fall in the last 12 months or a subjective feeling of a decrease in balance ability in the past year and a deficit in balance ability, defined as a loss of balance ability on the 8LBS to level 4 (tandem standing with eyes closed). | Community-dwelling | Author’s county: Germany |
| Unstable board  (DYJOC BOARD, SAKAI Medical Co., Ltd.) | Akizuki, 2018 | Validation (relationship with existing dynamic  balance indices) | Not reported | 59 | 70.5 ± 3.5 | 35.60 | Age ≥ 65 years; independent walking without the need of any supporting equipment; and able to come to the study venue independently. | Community-dwelling | Japan |
| The limits of stability (LOS) test | Clark, 1997 | Validation (reliability) | Not reported | 38 | 67.5 ± 8.4 | 55.26 | Community-dwelling healthy older adults (with no recent history of falls) | Community | Author’s country: USA |
| The Four Square Step Test (FSST) | Cleary, 2017 | Validation (properties of the FSST in older adults) | Not reported | 45 | 84.2 ± 6.3 | 69.00 | Be 65 years or older, able to provide informed consent, and able to walk inside their homes with or without an assistive device, but without help from another person. | Community | USA |
|  | Işik, 2015 | Validation (Test-Retest Reliability; Simultaneous Validity) | Cross-sectional descriptive study | 80 | 72.7 ± 5.1 | 45.00 | Healthy individuals aged 65–85; Absence of a neurological or orthopaedic problem that may prevent the tests; Absence of a vision or hearing impairment that may prevent the tests; No history of surgery of a lower extremity or the lumbar spinal region; Able to independently perform activities of daily living. | Nursing home | Authors’ country: Turkey |
|  | Dite, 2002 | Validation (reliability and validity; sensitivity, specificity, and predictive value) | Not reported | 81 | Multiple fallers 74.0 ± 5.7)  Non-multiple fallers :  73.8±6.1)  Comparison group: 74.1 ± 6.1 | Not reported | Comparison subjects had to be active and have no known neurologic or orthopedic condition affecting their balance or mobility. All subjects were (1) able to follow simple instructions and had a functional command of English, (2) able to give informed consent, (3) able to ambulate inside under close supervision, with or without a cane, for a distance of 6m, (4) not visually impaired, and (5) living in the community (not in supported accommodation). | A rehabilitation center and university medical school | Australia |
| The mediolateral balance assessment (MELBA) tool | Cofré Lizama, 2015 | Validation (predictive ability; ecological validity) | Not reported | 19 | 72.0 ± 5.0 | 36.84 | Healthy older adults with no history of falls over the previous 12 months. | Community | Netherlands |
| The Spring Scale Test (SST) | DePasquale, 2009 | Validation (test-retest reliability; convergent and known groups validity) | Descriptive study | 58 | 83.6 ± 5.6 | 67.24 | Living independently in the community without assistance; aged 65 years and older; capable of independent unrestricted community ambulation with or without a cane one or more blocks; medically stable, not requiring frequent medication adjustments or medical intervention; etc. | Community | Authors’ country: USA |
| The Microsoft Xbox One Kinect (Kinect v2) | Eltoukhy, 2018 | Validation (validity and reliability) | A cross-sectional study | 10 | 70.6 ± 9.5 | 50.00 | Recreationally active subjects with no history of lower extremity injury.  Elderly subjects (60–85 y) were recruited from the local community. Subjects had to be free from any neurological impairment that would affect balance and severe musculoskeletal impairment. | Community | Authors’ country: USA |
| The TURN 180 test | Fitzpatrick, 2005 | Validation (within one session, within-observer, retest reliability; repeatability factor and MDC) | Not reported | 66 | 82.5 ± 6.4 | 65.20 | Being 65 years of age or over, being mentally and physically able to perform TURN 180. | A day unit for elderly people accepting both inpatients and outpatients. | UK |
|  | Ranji, 2020 | Validation (Correlation with the BBS) | A cross-sectional study | 30 | Range: 60.0 to 80.0 | Not reported | Elderly population between 60 to 80 years of age, who have comorbidities, previous history of falls. | Community | Authors’ country: India |
| The Lower Quarter Y-Balance Test (LQ-YBT) | Freund, 2019 | Validation (reliability, normative values, and relationships to other balance measures) | Not reported | 60 | *50–59 yr*: 55.7 ± 2.8  *60–69 yr*: 63.6 ± 2.8  70–79 yr: 73.6 ± 3.0 | 100.00 | Able to walk independently with no assistive device, and no more than one fall in the past year. | Community | Authors’ country: USA |
| The Narrow Path Walking Test (NPWT) | Gimmon, 2013 | Validation (relative and absolute test–retest reliability; concurrent validity) | Baseline assessment in a prospective study | 30 | 81.8 ± 6.1 | 66.67 | Independent healthy older adults; age ≥ 65 years; being able to walk independently; having no serious visual impairments. | Protected retirement home for older adults | Israel |
| The One leg standing (OLS) | Giorgetti, 1998  ***Note***: 2 other tools assessed: TG & FR | Validation (inter-rater reliability) | Not reported | Sample 1: 21 (Subjects without disability) | Mean (range): 73.1 (69–85) | 66.67 | Non-disabled community-dwelling older subjects. | Community | Authors’s country: USA |
|  | Lin, 2004  ***Note***: 3 other tools assessed: TUG, TB & FR | Validation (practicality, reliability, validity, and responsiveness) | A prospective study | 1200 | Mean: 73.4 | 41.00 | People aged 65 and older. | Community-dwelling | Taiwan |
| Tandem Gait (TG) | Giorgetti, 1998  ***Note***: 2 other tools assessed: FR & OLS | Validation (inter-rater reliability) | Not reported | Sample 1: 21 (Subjects without disability) | Mean (range): 73.1 (69–85) | 66.67 | Non-disabled community-dwelling older subjects. | Community | Authors’s country: USA |
| The five-times-sit-to-stand test (FTSST) | Goldberg, 2012 | Validation (validity; relative and absolute reliability; minimum detectable change) | Not reported | 29 | 73.6 ± 7.4 | 100.00 | Female community-dwelling adults aged 60 years and older, able to walk at least 10 meters and stand at least 10 minutes without an assistive device, and alert and oriented. | Community-dwelling (Participants recruited at an urban senior center) | Authors’s country:  USA |
| The Maximum Step Length (MSL) test | Goldberg, 2010 | Validation (concurrent validity; intra- and interrater reliability and standard error of measurement) | Not reported | 35 | 72.8 ± 1.0 | 80.00 | Community-dwelling adults aged 60 years or older. | Community-dwelling | Authors’s country:  USA |
| The Thirty Rapid-Step test (30-RST) | Goldberg, 2015 | Validation (concurrent validity; Relative reliability; Absolute reliability, and MDC) | Cross-sectional observational study | 37 | 67.7 ± 5.3 | 75.70 | Community-dwellers aged 60 years or more, who were able to ambulate independently with or without an assistive device. | Community-dwelling | Authors’s country:  USA |
| The Community Balance and  Mobility Scale (CBM) | Weber, 2018 | Validation (concurrent validity,  inter- and intrarater reliability, internal consistency, and ceiling effects) | Cross-sectional study | 51 | 66.4 ± 2.7 | 74.50 | Community-dwelling older adults aged between 60 and 70 years, able to walk independently, and no cognitive impairment (Montreal Cognitive Assessment ≥ 26 points). | Community-dwelling | Germany, Norway, the Netherlands |
| The ***German***-Community Balance and Mobility Scale (German CBM) | Gordt, 2019 | Translation and validation (Construct validity; Reliability; Floor and ceiling effects; internal  consistency reliability) | Not reported | 51 | 69.9 ± 7.1 | 76.00 | Healthy community-dwelling older adults aged 55 years or older and able to walk independently for a minimum of 7 m. | Community-dwelling | Germany |
| The Shortened version of the Community Balance and Mobility Scale (s-CBM) | Gordt, 2020 | Development and validation  (Structural validity; Internal consistency; Construct validity; Discriminant validity) | Cross-sectional study | Sample 1: n = 189 (development);  Sample 2: n = 61 (validation) | Sample 1: 66.3 ± 2.5  Sample 2: 66.5 ± 2.6 | Sample 1: 52.40  Sample 2: 68.9 | Being retired, being able to walk 500 m without walking aid, and no cognitive impairment (Montreal Cognitive Assessment at least 24 points). | Community-dwelling | Germany; the  Netherlands; Norway |
| The “Step-Ex”  (New Development Technologies [NDT], Stockholm, Sweden) | Halvarsson, 2012 | Validation (Relative and absolute reliability) | Test–retest design | Total = 34  Healthy: 18  With history of falls: 16 | Healthy: 73.0  With history of falls: 77.0 | Healthy: 50.00  With history of falls: 62.50 | Group 1: Elderly people who regarded themselves as healthy and without a history of falls, fear of falling or balance problems (Healthy).  Group 2: People with a history of falls during the previous year and balance deficits (With history of falls). | Community-dwelling | Sweden |
| Tinetti's POMA balance subscale | Harada, 1995  ***Note***: 1 other tool assessed: BBS | Validation (Sensitivity, Specificity) | Not reported | 53 | 83.3 ± 7.7 | 87.00 | A convenience sample of elderly subjects.  (No specific inclusion criteria reported.) | Nursing homes | USA |
|  | Lin, 2004  ***Note***: 3 other tools assessed: TUG, OLS & FR | Validation (practicality, reliability, validity, and responsiveness) | A prospective study | 1200 | Mean: 73.4 | 41.00 | People aged 65 and older. | Community-dwelling | Taiwan |
| The Short Berg Balance Scale  (BBS-9) | Hohtari-Kivimaki, 2012 | Development and validation (Factor analysis; Internal consistency; Correlations with other balance measures) | Not reported | 519 | Mean (range): 72.4 (65 − 91) | 84.00 | Subjects were 65 years of age or over with good or moderate cognitive abilities (Mini Mental State Examination ≥17), at least one fall during the previous 12 months, and the ability to walk 10 meters independently, with or without walking aids. | Community-dwelling and nursing home (home or sheltered housing) | Finland |
| The Multi-Directional Reach Test (MDRT) | Holbein-Jenny, 2005  ***Note***: 1 other tool assessed: the BBS | Validation (test-retest reliability; inter-rater reliability; construct validity) | Not reported | 26 | 85.3 ± 4.9 | 80.77 | (1) An age of at least 65 years, (2) capability of flexing and holding the shoulder at 90° of flexion or abduction for the MDRT, and (3) ability to stand for each component of the test without the use of an assistive device. | Personal Care Home | USA |
|  | Newton, 2001 | Validation (reliability and validity) | Not reported | 254 | 74.1 ± 7.9 | 78.50 | Ccommunity-dwelling older adults from senior centers or residential housing centers located in North Philadelphia, a federally designated medically underserved area. Older adults were excluded if they were wheelchair bound or could not lift both outstretched arms to 908 in the forward direction. | Community-dwelling older adults from senior centers or residential housing centers. | USA |
| The Kinect system (Kinect for Xbox 360™, Microsoft Corp, Seattle, WA, USA) | Hsiao, 2018 | Validation (reliability and correlations with the traditional measures) | Observational study | 442 | 73.3 ± 5.2 | 52.26 | Individuals older than 65 years of age who underwent a geriatric health examination between January and December 2013 at the National Taiwan University Hospital Bei-Hu Branch were invited to participate in the study. | Community-dwelling | Taiwan |
| The ***Turkish version*** of Fullerton Advanced Balance (FAB-T) scale | Iyigun, 2018 | Translation and validation (reliability and validity) | Not reported | 200 (validation study) | 70.4 ± 5.1 | 51.50 | Older adults aged between 65 to 85 years. Individuals who were able to walk independently with or without assistive device and who scored above 21 on the Standardized Mini Mental Test (SMMT). | Community-dwelling | Turkey |
| The Fullerton Advanced  Balance (FAB) Scale | Klein, 2011 | A study “to examine selected measurement properties of the FAB Scale by applying Rasch analysis” | Cross-sectional study | 480 | 76.4 ± 7.1 | 71.00 | Community-dwelling adults aged 60 years and older who were able to ambulate independently without an ambulatory aid during test administration. | Community-dwelling | USA |
|  | Rose, 2006 | Development and validation (reliability and content and convergent validity) | Design: Psychometric evaluation | 46 | 75.0 ± 6.2 | Not reported | Community-residing older adults  with and without identified balance problems (Functionally Independent Older Adults). | Urban community | Authors’ county: USA |
| The parallel walk test | Lark, 2009 | Validation (validity) | Control study | Fallers: 27  Non-fallers:34 | Fallers : 82.0 ± 6.0  Non-fallers : 76.0 ± 7.0 | Not reported | A score of greater than or equal to 23 on the Mini Mental State Examination for mental cognitive ability and a score greater than 10 for the Barthel Index, which assesses the performance of activities of daily living. | Community-dwelling | Authors’ county: New Zealand |
| The timed up and go (TUG) test | Galhardas, 2020  ***Note***: 1 other tool assessed: the FR | Validation (Test-retest reliability) | A test-retest reliability study | 53 | 85.9 ± 3.9 | 77.36 | Being aged 75 years or older; living in a nursing home; and having a normal cognitive status according to the Portuguese version of the Mini-Mental State Examination | Nursing home | Portugal |
|  | Lin, 2004  ***Note***: 3 other tools assessed: OLS, TB & FR | Validation (practicality, reliability, validity, and responsiveness) | A prospective study | 1200 | Mean: 73.4 | 41.00 | People aged 65 and older. | Community-dwelling | Taiwan |
|  | Nightingale, 2019 | Validation (correlations between the TUG Test and various balance markers utilizing the OptoGait system) | Not reported | 51 | Age ≥ 65.0 | Not reported | Participants recruited via flyers placed at local community centers, health care provider offices, and senior centers.  (No specific inclusion criteria reported) | Community | Authors’s country: USA |
|  | Pelicioni, 2022  ***Note***: 3 other tools assessed: BBS; DGI; and FGA. | Validation (reliability; validity)  ***Remark***: Assessments conducted remotely via telehealth (ZOOM^®^). | A pilot study | 15 | Mean (range): 71.7 (64.0–78.0) | 53.33 | Having access to a mobile device (e.g., smartphone, laptop, tablet) with high-speed internet; being familiar with, or having support person familiar with, mobile technology; being able to walk 30 m with or without a walking aid and stand without support for 1 min. | Community-dwelling | New Zealand |
|  | Yingyongyudha, 2016  ***Note***: 3 other tools assessed: BBS; BESTest; Mini-BESTest | Validation (identifying  older adult with history of falls, based on balance scores) | Not reported | 200 | Without  History of Falls: 70.2 ± 6.8  With  History of Falls: 70.3 ± 7.3 | Total: 63.00 | (1) 60 years of age or older; (2) ability to walk without using walking aids; (3) independence in basic daily activities; (4) no history of neurological diseases such as Parkinson's disease or stroke; and (5) no severe knee pains that affected walking on the day of the assessment. | Community | Thailand |
| The Balance Computerized Adaptive Testing (Balance CAT) | Lu, 2015 | Validation (concurrent validity, discriminative validity, reliability, and efficiency) | A cohort study | 120 | 72.0 ± 12.7 | 47.50 | (1) Stable physical and psychological condition confirmed by the senior nurse in the LTC facility, and (2) ability to follow simple instructions to complete the interview and performance testing. | Long-term care (LTC) facilities | Taiwan |
| The MyBalance test | Mansson, 2021  ***Remark***: A tool used for 2 different tests, Balance and muscle strength | Validation of a prototype  (Concurrent validity) | An observational explorative study | 31 | 78.7 ± 4.7 | 77.42 | ≥70 years old, able to rise from a chair independently, community-dwelling, and able to understand and read Swedish. | Community (outpatient physiotherapy setting) | Sweden |
| The Brief‐Balance Evaluation Systems Test (Brief-BESTest) | Marques, 2016  ***Note***: 3 other tools assessed: Mini-Best, Best, and BBS | Validation (reliability, validity, and ability to identify fall status) | Cross-sectional. | 122 | 75.9 ± 8.9 | 70.50 | (1) Aged ≥ 60 years; (2) living independently in the community; and (3) able to ambulate with or without a walking aid (but without the assistance of another person). | Community | Portugal |
|  | O'Hoski, 2015  ***Note***: 2 other tools assessed: mini-BESTest and BESTest | Validation (convergent  Validity; discriminative ability) | Cross-sectional study (Secondary analysis) | 79 | 68.7 ± 10.6 | Not reported | (1) Age between 50 and 89 years, (2) residing independently in the community, (3) able to understand and follow three-step instructions, (4) able to walk 6 m independently (without a gait aid). | Community | Authors’ county: Canada |
|  | Viveiro, 2019  ***Note***: 3 other tools assessed: BBS; Mini-BESTest; BESTest. | Validation (reliability, validity, and ability to identify  fall status) | Cross-sectional study | 49 | 77.8 ± 7.2 | 61.20 | (1) 60 years or older; (2) both sexes; (3) ability to stand with or without the aid of a support device; (4) residence in the nursing home; and (5) provision of written informed consent. | Nursing home | Brazil |
| The Functional Gait Assessment-***Brazil***  (FGA- Brazil) | Marques, 2021 | Validation (construct and criterion validity; and ceiling and floor effects.) | Not reported | 121 | 69.3 ± 7.4 | 69.40 | Men and women aged 60 years or older, living in the community, able to ambulate with or without assistive devices, able to stand up independently, and able to understand verbal commands. | Community-dwelling  (Recruited at an ambulatory care center) | Brazil |
|  | Kirkwood, 2021 | Translation, cross-culturally adaptation, and validation (inter- and intra-rater reliability, internal consistency) | Not reported | 70 | 70.0 ± 7.8 | 72.90 | 60 years or older, living independently in the community, able to stand up for at least one minute, and a minimum score of 17 on the Mini-Mental State Examination | Community-dwelling | Brazil |
| The ‘‘Get-up and Go’’ Test | Mathias, 1986 | Validation (Observer variation; Test score vs sway.) | Not reported | 40 | Mean (range): 73.8 (52.0 − 94.0) | 50.00 | Inpatients, outpatients, or day patients of the medical and geriatric departments of Selly Oak Hospital, Birmingham, United Kingdom.  (No specific inclusion criteria reported) | Community (inpatients, outpatients, or day patients) | United Kingdom. |
| The apparatus for assessment of postural responses | Matjacic, 2010 | Validation (Comparison between fallers and non-fallers; Correlation with BBS scores) | Not reported | 20 | Non-fallers: 75.6 ± 10.2  Fallers: 84.6 ± 4.2 | Non-fallers:  80.00  Fallers: 100.00 | Able to maintain quiet stance for at least one minute, no diagnosis of neurological diseases and successfully pass the Mini-Mental State Examination, a short test of cognitive abilities. | Elderly home | Authors’ country: Slovenia |
| A comprehensive set of inertial sensor measures of postural sway  (The Balance Score (BS) & The Weighted Balance Score (WBS)) | Mcmanus, 2022 | Validation (reliability; clinical validity) | Not reported | 248 | 74.9 ± 6.5 | 63.31 | Subjects aged 60 years and older, who had no history of stroke and were able to walk unassisted. | Community dwelling | Ireland |
| The Modified Version of the Community Balance and Mobility Scale (CBMS-Home) | Ng, 2021 | Validation (Principal  components analysis, internal consistency, test-retest and inter-method reliability, agreements within and between methods,  and criterion validity) | A validation study | 55 | 77.2 ± 6.0 | 74.50 | Community-dwelling people 65 years old or older, ability to understand spoken and written English, and ability to walk independently with or without a walking aid. Older adults who had chronic neurological problems, such as stroke and Parkinson disease, who  met the inclusion criteria were eligible to participate (4 people). | Community-dwelling | Authors’s country:  Australia |
| The Pavia  Instrumented Tinetti Test (PITT) | Panella, 2008 | Validation (internal consistency reliability; validity) | Not reported | Control health: 163  Patients: 111 | Control healthy: Age range, 19.0 – 85.0  Patients: 78.6 ± 9.0 | Controls: 56.44  Patients: 70.27 | Controls: Subjects that did not complain of any pathology of the lower limbs and/or central or peripheral balance control system and had scored 28/28 in the Tinetti test.  Patients: Scored less than 14/14 in the subset of Tinetti test manoeuvres considered by the PITT, but was able to understand the relevant instructions and to carry out the PITT autonomously. | Control: Not reported  Patients: Old people’s homes | Italy |
| The Dynamic Gait Index  (DGI) | Pelicioni, 2022  ***Note***: 3 other tools assessed: BBS; FGA; and TUG. | Validation (reliability; validity)  ***Remark***: Assessments conducted remotely via telehealth (ZOOM^®^). | A pilot study | 15 | Mean (range): 71.7 (64.0 –78.0) | 53.33 | Having access to a mobile device (e.g., smartphone, laptop, tablet) with high-speed internet; being familiar with, or having support person familiar with, mobile technology; being able to walk 30 m with or without a walking aid and stand without support for 1 min. | Community-dwelling | New Zealand |
| The ***Danish Version*** of the Dynamic Gait Index (DGI) | Jønsson, 2011 | Validation  (Intra-rater and inter-rater reliability;  and agreement) | Reliability study | Rehabilitation Center (n=24) | 76.8 ± 6.4 | 58.00 | (1) age at least 65 years, (2) ability to walk a minimum of 6m with or without a walking aid, and (3) 1 or more falls in the last year or (4) observed balance impairment evaluated subjectively by a physical therapist during the initial training session. | Community-dwelling | Denmark |
| The Functional Gait  Assessment (FGA) | Pelicioni, 2022  ***Note***: 3 other tools assessed: BBS; DGI; and TUG. | Validation (reliability; validity)  ***Remark***: Assessments conducted remotely via telehealth (ZOOM^®^). | A pilot study | 15 | Mean (range): 71.7 (64.0 –78.0) | 53.33 | Having access to a mobile device (e.g., smartphone, laptop, tablet) with high-speed internet; being familiar with, or having support person familiar with, mobile technology; being able to walk 30 m with or without a walking aid and stand without support for 1 min. | Community-dwelling | New Zealand |
|  | Wrisley, 2010 | Validation  (Concurrent, discriminative, and predictive validity) | A prospective cohort study | 35 | 72.9 ± 7.8 | 51.43 | Aged between 60 and 90 years; lived independently in the community; were able to stand independently longer than 1 minute; and had a Mini-Mental State Examination score of greater than 24. | Community | USA |
|  | Beninato, 2016 | Validation Through  Rasch Modeling | A retrospective chart review | 179 | 79.0 ±7.0 | 63.70 | A minimum of 60 years of age, able to walk independently with or without an assistive device, and referral for physical therapy for balance retraining. | Community dwelling | Authors’ country: USA |
| The NIH Toolbox^®^ Standing Balance Test | Peller, 2022 | Validation (validity; reliability) | An observational cross-sectional design. | 93 | 73.0 ± 6.1 | 59.14 | Above the age of 60 and able to walk 20 feet without an assistive device. | Community-dwelling | USA |
| The Biodex SD (Biodex Medical Systems, Shirley NY) | Riemann, 2017 | Validation (test–retest reliability of  self-selected and narrow stance balance testing) | Not reported | 30 | Men: 71.2 ± 9.2  Women: 72.4 ± 6.7 | 50.00 | All participants were void of uncorrected visual impairments,  neurological disorders (stroke, Parkinson’s disease, and multiple sclerosis), lower limb surgery, or cognitive impairments based on their completion of a comprehensive medical questionnaire. | Community-dwelling & Community senior centers | Authors’ county: USA |
| The Balance Scale (by Roberts) | Roberts, 1987 | Validation (construct  validity and reliability) | Not reported | 61 | 71.9 ± 5.7 | 85.30 | A sample of 61 community living elders.  (No specific inclusion criteria reported) | Community | Authors’ country: USA |
| The ***Turkish Version*** of the Berg Balance Scale (BBS) | Sahin, 2008 | Translation and Validation (Reliability and  Validity) | Not reported | 60 | 77.0 ± 5.7 | 75.00 | At least 65 years of age, and to speak the Turkish language. | Nursing Home and community | Turkey |
| The ***Persian version*** of the Berg Balance Scale (BBS) | Salavati, 2012 | Translation and validation (inter and intra-rater reliability and construct  validity) | Not reported | 106 | 65.2 ± 5.6 | 75.00 | Ability to stand and walk independently without use of walking aids and ability to speak the Persian language. | Community | Iran |
| The Nintendo Wii Fit *exergame* | Sato, 2021 | Validation (concurrent validity) | A cross-sectional study | 20 | 73.6 ± 5.6 | 75.00 | Aged between 18 and 90 years old; community dwelling; independent in daily activities; no previous experience with Wii Fit; and body weight less than 150 kg. | Community dwelling | Japan |
| The Wii Stillness (WST) Test | Simms, 2020 | Validation (Concurrent validity) | A cross-sectional study | 26 | 81.3 ± 7.8 | 61.00 | (a) Being at least 60-years-old; (b) reporting no lower body musculoskeletal injuries within the past 6 months; (c) able to stand without an assistive device; and (d) cognitive abilities to understand the  proposed tasks. | Senior housing facility and Community | USA |
| The short form of the Fullerton Advanced Balance (SF-FAB) scale | Sinaei, 2021 | Validation (inter- and intrarater reliability; fall-risk predictability) | A cross-sectional study | 85 | 70.75 ± 4.97 | 78.80 | The eligibility criteria included the following: 65 years or older, no  cognitive impairment based on a Mini-Mental Scale Examination score of 24 or more, the ability to walk independently for 20 m,  and the ability to communicate verbally. | community-dwelling | Shiraz (Iran) |
| The 'balance meter' | Stokes, 1998 | Validation (reliability; comparison fallers versus non-fallers; comparison with a functional text) | Not reported | Non-fallers: 50  Fallers: 25 | Non-fallers Mean, 70.8  Fallers: Mean, 77.6 | Not reported | Non-fallers: Aged 60 years or over, being functionally independent, and being mentally capable of complying with the test procedure.  Fallers: Aged 60 years and over, and a reported history of two or more falls in the year prior to testing. | Community | Authors’ country: Ireland |
| The AMTI Accusway system for balance and postural sway  measurement (Advanced Mechanical Technology, Inc.,  Watertown, Massachusetts) | Swanenburg, 2008 | Validation (reliability) | Not reported | 37 | 73.0 ± 6.0 | 78.37 | Fallers and non-fallers older than 60 years of age of both genders. | Community-dwelling | Switzerland |
| A dual-task computer game-based platform (TGP) | Szturm, 2015 | Validation (Test-retest reliability) | Not reported | 30 | Median (range): 64.0 (60.0 – 67.0) | 80.00 | Living independently in the community, able to walk outside without any walking aids, and no self-reported history of falling. | Community-dwelling | Canada |
| The Modified Bathroom Scale | Vermeulen, 2012 | Validation (construct validity) | Cross-sectional study | Total: 101  Nursing Home: n = 47  Community: n = 54 | Nursing Home: 80.6 ± 6.4  Community: 75.7 ± 5.1 | Nursing Home: 66.00  Community: 90.70 | Aged 65 years or older and able to step onto a bathroom scale independently. | Nursing home and community-dwelling. | The Netherlands |
| The instrumented modified Clinical Test of Sensory Interaction on Balance (i-mCTSIB) utilizing the  Neurocom Very Simple Rehab (VSR) Sport force plate (Natus Medical  Incorporated, Pleasanton, California). | Watson, 2021 | Validation (test-retest reliability, standard error of measurement,  and minimal detectable change) | A methodological study | 20 | 74.3 ±4.1 | 75.00 | Participants qualified if between the ages of 65 and 85 years and able to stand for 5 minutes or more. | Community | USA |
| Models for estimating decline in balance using accelerometry-based gait features | Simila, 2017 | Models development and validation (against BBS, TUG and 4 m walk tests; prediction of decline in balance) | Not reported | 35 | 73.9 ± 5.4 | 100.00 | The subject should be living independently, have no cognitive incapability and is able to independently perform simple physical tasks. | Senior  house and community | Finland |
| The FICSIT Balance Scales (FICSIT-3 and FICSIT-4) | Rossiter-Fornoff, 1995 | Construction of a composite balance measure and validation (test-retest reliability; construct validity) | Data from a set of randomized clinical  trials and a non-randomized feasibility study. | All sites, N total = 2.559 | Range of mean age: 72.9 to 80.6 | Not reported | The FICSIT sites had different entrance and exclusion criteria. Consequently, some research subjects (from Farmington, Atlanta, and Seattle) were fairly healthy individuals, while others were very frail nursing home residents (San Antonio and Boston). | Nursing home and Community | USA |
| The Wii Balance Board™ (WBB) | Olvera-Chavez, 2013 | Validation (effectiveness of the WBB in balance assessment) | A cross-sectional study | 20 | Median (range): 67.5 (60.0 – 98.0) | 75.00 | Elderly 60-year or older community dwelling subjects who accepted to participate and signed informed consent declarations. | Community dwelling | Mexico |
|  | Scaglioni-Solano, 2014 | Validation (validity and  Reliability) | Not reported | 37 | 69.0 ± 8.0 | Not reported | Older adults who regularly (at least once a week) participated in exercise groups (dance, aerobics, tai chi, and other physical activities) and were able to independently arrive at their classes, and thus considered ‘highly functional’. In addition, participants had no known visual, vestibular, or neurologic impairment. | Community dwelling | Authors’ country: Costa Rica |
| The Balance Tracking System (BTrackS) | Levy, 2018 | Validation (Internal consistency; concurrent validity; test-retest reliability) | Not reported | Concurrent validity study: n=49  test-retest reliability: n=47 | Concurrent validity study,  71.3 ±7.3  Test-retest reliability, 75.8 ± 7.7 | Concurrent validity study, 53.06  Test-retest reliability study, 65.96 | Being ambulatory, free of any injury to the hip, lower limbs, or back in the past 6 months, and being able to stand unassisted for 3 minutes (the average amount of total testing time). | Community dwelling | USA |
| The NeuroCom Smart Equitest Research System (Natus Medical Inc, Pleasanton,  California) | Harro, 2019 | Validation (Reliability and validity) | Not reported | 46 | 67.7 ± 5.1 | 52.17 | (1) 60 to 80 years old, (2) functional vision with or without corrective lenses, (3) able to walk 300 ft with or without an assistive device, and (4) able to independently ascend and descend 6 stairs with or without a railing or assistive device. | Community-dwelling | USA |

**Appendix 2.b**: Characteristics of the included studies on muscle strength assessment tools

| **Instrument** | **Study reference** | **Type of study**  (As stated in the objectives) | **Study design** | **N** | **Age, years**  Mean ± SD | **% Female** | **Inclusion criteria** | **Setting** | **Country** |
| --- | --- | --- | --- | --- | --- | --- | --- | --- | --- |
| The JAMAR hand-held hydraulic dynamometers (pressure, pinch, platform/anchor) | Abizanda, 2012 | Validation (validity and reliability) | Cross-sectional study. | 281 | 74.3 ± 4.9 | 63.35 | Subjects aged over 65 years from the  community, recruited voluntarily in senior centers where they participated in recreational activities and exercise programs, and independent for all basic activities of daily living (ADL). | Community | Spain |
|  | Silva, 2019 | Validation (reliability and measurement error) | Not reported | Total: 100  Nursing homes (n =70)  Day Care Centers (n =30) | Total: 82.3 ± 8.1  Nursing homes: 83.2 ± 7.4  Day Care Centers: 80.2 ± 9.4 | Total: 62.00  Nursing homes: 60.00  Day Care Centers: 66.70 | Participants scoring 4 or less in the Short Portable Mental Status Questionnaire (SPMSQ). A score between 0 and 2 is indicative of normal cognitive functioning; a score between 3 and 4 is indicative of mild cognitive impairment. | Day Care Centers and Nursing Homes | Portugal |
| The MicroFET2 hand-held dynamometer  (Hoggan Indiustries, Inc., West Jordan, UT, USA) | Buckinx, 2017 | Validation (test–retest reliability: intra- and inter-observers) | Cross-sectional study | 30 | 75.0 ± 11.2 | 50% | Living in a nursing home and being able to stand (with or without technical assistance). | Nursing home residents | Belgium |
| A uni-axial load cell device | Alqahtani, 2019 | Validation (test–retest reliability and construct validity) | An ancillary study of a cluster randomized clinical trial (RCT). | 131  Validity (n=131)  Reliability (n = 38) | Validity: 80.3 ± 7.7  Reliability: 76.4 ± 6.5 | Validity: 85.00  Reliability: 87.00 | (1) 65 years of age or older; (2) a resident of a University of Pittsburgh Medical Center (UPMC) independent living facility (ILF), senior high rise, or a senior community center; (3) ability to ambulate independently within the household with or without a straight cane; and (4) gait speed greater than or equal to 0.60 m/s. | Community  Settings (independent living facility or senior community center) | USA |
| The calf-raise senior (CRS) test | Andre, 2016 | Development of the test protocol and validation (reliability and validity) | Involved  five cross-sectional studies | All studies, Total N=41  Pilot: 12  Inter- and intrarater: 12  Test–retest: 41  Construct: 41  Criterion: 33 | 73.9 ± 7.7 | 56.10 | Subjects aged 65 years and older, of both sexes, …. randomly selected from day care centers, senior schools, fitness centers, and community physical activity (PA) programs. This procedure aimed at establishing a cohort of older adults with different levels of PA and functional fitness. | Community | Portugal |
| Handheld Dynamometry (HHD)  The Lafayette Manual Muscle Tester, Model # 01163, (Lafayette Instrument Inc.,  Lafayette, Indiana) | Arnold, 2010 | Validation (intra-rater and inter-rater  reliability; validity, by comparing measurements to the gold standard) | A repeated measures design | 18 | Mean (range): 74.0 (65.0–92.0) | 77.78 | (a) aged 65 years or older and (b) living independently in the community. | Community | Canada |
|  | Bohannon, 2005 | Validation (Test–Retest Reliability) | Longitudinal measurement of grip strength over a 12-week period. | 21 | 75.0 ± 5.9 | 80.95 | Apparently healthy community-dwelling men and women. All were at least 65 years of age. They were independently ambulatory and community dwelling. | Community | USA |
|  | Bohannon, 1997 | Validation (Internal consistency) | Retrospective study | 37 (with analyses on variable number of patient) | Mean (range): 77.7 (36.0-94.0) | 59.46 | A sample of home care patients.  The records of 37 consecutive patients were examined. | Home care patients | Author’s country: USA |
|  | Martin, 2006  ***Muscle group***: The supine quadriceps  strength | Validation (validity: compared with gold standard Biodex system II dynamometer) | Not reported | 20 | 72.6 ± 5.0 | 55.00 | Participants aged 61–81 years, recruited through local retirement clubs, churches, and activity clubs. | Community | UK |
| The Nintendo Wii Balance Board (WBB) | Blomkvist, 2016 | Validation (relative and absolute reproducibility & concurrent validity) | Intra-rater test-retest cohort design with randomized validity testing on the first session | 30 | 69.0 ± 4.2 | 60.00 | Participants were included if they were 65 years or more, willing, and able to come to the hospital twice within a week by themselves, and able to pass a small custom dementia screening. | Community | Denmark |
|  | Jorgensen, 2015  ***Muscle group:***  isometric muscle strength in the lower limb | Validation (reproducibility; concurrent validity) | intra-rater inter-day design | 30 | 69.0 ± 4.2 | 60.00 | Participants included if they were 65 years or more, willing and capable of coming to the hospital by themselves twice within a 7-day span and could pass a small custom cognitive impairment screening (answering the current year, month and prime minister of Denmark). | Community | Denmark |
| The Modified Sphygmomanometer Test (MST) | Brito, 2022 | Validation (test-retest/inter-rater reliabilities and criterion-related validity & SEM and SRD) | Not reported | 50 | 69.9 ± 5.5 | 58.00 | Community-dwelling older adults, both men and women, age ≥ 60 years old (per definition of older adults for people living in developing countries according to the World Health Organization) and with a body mass index ≤ 29.9 kg/m^2^. | Community | Authors’ country: Brazil |
| The isometric knee extension (IKE) test  (IKE test + strain gauge) | Buendía-Romero, 2021 | Validation (intra- and inter-session repeatability) | Part of an ongoing multicentre, randomized controlled trial (the HEAL study) | 13 | 87.0 ± 10.9 | NA | Inclusion criteria for the HEAL study: “men and women aged ≥70 years, be able to follow an active physical rehabilitation program and voluntary participation. Enrolment of cognitively impaired older adults will require proxy permission (family member or caregiver)” | Nursing home | Spain |
| The Q Force | Douma, 2016 | Validation (test-retest reliability) | Not reported | 41 | 81.9 ± 4.9 | 68.29 | At least 70 years of age, being able to walk ≥10 m without support, and rise from a chair without resources or assistance; Absence of cardiovascular/respiratory or neurological disorders; No comorbidity or cognitive disorders that influence mobility, understanding, or execution of measurements. Etc. | Community | The  Netherlands |
| An analog dynamometer (SENSIX®, Poitiers, France) coupled with the DELSYS System (Trigno sensor, DELSYS, INC Boston; MA)  (A dynamometer fixed to a custom-made frame for a hip abductor and  Adductor test) | Gafner, 2017  Hip abductor and adductor maximum voluntary isometric strength (MVIS) and rate of force generation (RFG) test. | Validation (feasibility and repeatability/test-retest reliability) | A measurement focused study | 76  (Half of them underwent the abduction and the other half, the adduction test) | 80.5 ± 7.1 | 53,95 | Participants had to be over 65 years. They were excluded if their medical record contained a history or evidence of any significant central nervous system dysfunction, any neuromuscular disorder other than a distal symmetric peripheral neuropathy, or evidence of vestibular dysfunction. (+ other exclusion criteria) | Community (Geriatric hospital and outpatients practice). | Switzerland |
| The Biodex System 3 isokinetic dynamometer  (Biodex Medical Systems, Shirley, N.Y., USA) | Hartmann, 2009 | Validation (inter- and intrarater reliability) | Not reported | 24 | 71.2 ± 5.5 | 75.00 | Independently living older subjects (a minimum age of 65 years). | Elderly residence and local community | Switzerland |
|  | Symons, 2004 | Validation (test–retest reliability) | Not reported | 25 | 72.0 ± 6.0 | 100.00 | Participants were free of any cardiovascular or lower limb neuromuscular and musculoskeletal limitations. | Community | Canada |
| Isokinetic dynamometer (KinCom 500H, Chattecx Corp., Hixson, TN, USA) | Holsgaard Larsen, 2007  ***3 muscle groups tested***:  Quadriceps;Hamstring; Plantar flexors.  ***Note***: also assessed another tool for Power | Validation (test–retest reproducibility) | Not reported | 18 | 72.3 ± 6.6 | 100.00 | The subjects participated in multi-component activities once per week and were considered as moderately trained. … None of the participants had a history of orthopedic or neurological disorders and did not report any bone fractures in the lower extremities within the last 5 years. | Community-dwelling | Authors’ country: Denmark |
| The Leg Press Sled (LPS) | Hutchison, 2006 | Validtation (reliability; concurrent validity) | Prospective validation study | 11 | 81.7 ± 7.0 | NA | Ability to both rise from a seated position with minimal assistance and to walk at least 20 steps with at most a walker or cane. | Assisted-living facility | USA |
| The Microfet 2000 strain gauge portable dynamometer (PD) | Karner, 1998 | Validation (intrarater and interrater  reliability) | Not reported | 15 | Mean age= 83.3 | 100.00 | Subjects were residents at Marian Villa, a Home for the Aged in London, Ontario. All participants were capable of independent ambulation with or without gait aid and were able to follow simple instructions. | Nursing home | Canada |
| A load cell setup | Keshavarzi, 2022  ***Muscle group***: isometric back extensor strength. | Validation (intra-rater test-retest reliability) | Cross-sectional observational study | 25 healthy subjects (control group) | 67.2 ± 4.5 | 56.00 | Older adults over the age of 60 who could stand and walk without assistance were included in the study. | Community-dwelling (“…recruited through public advertisement) | Iran |
| The push-off test (POT) | Legg, 2020  ***Note***: 1 other tool assessed for strength: The functional multi-joint isokinetic dynamometer) | Validation (test–retest reliability and concurrent validity) | A repeated measures design | 17 | 71.0 ± 10.0 | 64.71 | A convenience sample of 20 participants over the age of 60 years was recruited from the local community via posters and a posting on the University intranet noticeboard. | Community-dwelling | Canada |
| The functional multi-joint isokinetic dynamometer (concentric (CON) and eccentric (ECC)  strength) | Legg, 2020  ***Note***: 1 other tool assessed for strength: The POT | Validation (test–retest reliability and concurrent validity) | A repeated measures design | 17 | 71.0 ± 10.0 | 64.71 | A convenience sample of 20 participants over the age of 60 years was recruited from the local community via posters and a posting on the University intranet noticeboard. | Community-dwelling | Canada |
| The MyBalance test | Mansson, 2021  ***Note***: A tool for 2 different tests, Balance and muscle strength | Validation of a prototype  (Concurrent validity) | An observational explorative study | 31 | 78.7 ± 4.7 | 77.42 | ≥70 years old, able to rise from a chair independently, community-dwelling, and able to understand and read Swedish. | Community (outpatient physiotherapy setting) | Sweden |
| The maximal isometric strength test of the trunk  (measured by a precalibrated digital loading cell connected to the MuscleLab software) | Mesquita, 2019 | Validation (Reliability) | Not reported | 21 | 64.0 ± 4.0 | 100.00 | Physically inactive elderly women, who had not engaged in any activity or exercise program in the past three months, (a) age above 60 years, (b) no limiting back pain in the previous year, and (c) no medical or physiotherapeutic treatment for back pain in the previous year. | Community-dwelling | Brazil |
| The one-repetition maximum (1 RM)  (using elastic resistance bands test) | Nyberg, 2014 | Validation (validity) | Not reported | 30 | 60.2 ± 6.9 | 50.00 | Participants had to be 50 years of age or older, without any current musculoskeletal disorders in the upper extremity. | Community-dwelling | Sweden |
| The lateral step (LS) test  (For the Indirect Assessment of Hip Abductor Muscle Strength) | Porto, 2020  ***Note***: 2 other tools assessed, TG and SS | Validation (To determine the accuracy of the test for the identification of reduced hip abductor  muscle strength) | Cross-sectional study | 123 | 68.9 ± 5.4 | 100.00 | Independent older women aged 60 years or older. Due to the more pronounced dysfunctions in females, the present study opted to include only older women. | Community | Brazil |
| Tandem Gait (TG)  (For the Indirect Assessment of Hip Abductor Muscle Strength) | Porto, 2020  ***Note***: 2 other tools assessed LS and SS | Validation (To determine the accuracy of the test for the identification of reduced hip abductor  muscle strength) | Cross-sectional study | 123 | 68.9 ±5.4 | 100.00 | Independent older women aged 60 years or older. Due to the more pronounced dysfunctions in females, the present study opted to include only older women. | Community | Brazil |
| Single-leg stance (SS) test  (For the Indirect Assessment of Hip Abductor Muscle Strength) | Porto, 2020  ***Note***: 2 other tools assessed, TG and LS | Validation (accuracy of the test for identification of reduced hip abductor  muscle strength) | Cross-sectional study | 123 | 68.9 ± 5.4 | 100.00 | Independent older women aged 60 years or older. Due to the more pronounced dysfunctions in females, the present study opted to include only older women. | Community | Brazil |
| The one repetition maximum (1 RM)  (using a muscle strength training device for the arm/shoulder [Pull Down, Norway]) | Rydwik, 2007 | Validation (test-retest; and to compare subjects with and without  previous muscle strength training experience) | Reliability study | 34  Group 1 (n=23)  Group 2 (n=11) | *Group 1*:  Women: 80.3 ± 3.1; Men: 81.5 ± 3.1  *Group 2*:  Women: 85.4 ± 1.7; Men: 83 ± 4.7 | *Group 1*: 43.47  *Group 2*: 45.45 | Subjects recruited from a database of  1700 subjects that previously had shown interest in participating in research concerning nutrition and physical activity, aged 75 years and older. Two groups were recruited: one group with no previous muscle strength training experience (*Group* *1*) and one group who had already experienced muscle strength training (*Group 2*). | Community | Sweden |
| The five-repetition sit-to-stand (STS) test | Schaubert, 2005 | Validation (Reliability) | Not reported | 21 | 75.0 ± 5.9 | 80.95 | Nondisabled individuals who were at least 65 years of age and who were independently ambulatory and  community-dwelling were eligible to participate. | Community-dwelling (recruited at a local senior center) | Authors’ country: USA |
| A standardized heel-rise test  (using trunk accelerometry).  (For vertical ground reaction ***force*** and external mechanical ***power***) | Schmid, 2011  ***Note***: same tool used for muscle strength and power | Validation (intrasession reliability; concurrent validity) | Cross-sectional study | 54 | 81.2 ± 6.4 | 74.07 | A minimum age of 65 years and the ability to perform at least one of the following daily-living activities without assistance: standing up from a normal chair, walking 10 meters, and climbing up and down a stair with six steps. | Retirement homes | Switzerland |
| The one-repetition maximum (1 RM)  [performed on the Keiser A-300 pneumatic equipment (Keiser Corp., Fresno, CA) or on selectorized weight-stack resistance exercise machines (Cybex VR2; Cybex International Inc., Medway, MA)]. | Schroeder, 2007  ***Note***: 1 other tool for power assessed in this study | Validation (Reliability) | Data from 3 RCTs, forming 2 cohorts (*Cohort 1* & *Cohort 2*) | N = 116  *Cohort 1* (n = 90)  *Cohort 2* (n = 26) | Total, range: 60.0–87.0  *Cohort 1:* 72.0 ± 5.0  *Cohort 2:* 69.0 ± 3.0 | 0% | Participants must not have participated in regular resistance training, physical activity (with the exception of a walking program), or competitive sports for the previous 6 months. Participants had to have a body mass index (BMI) ≤ 35 kg/m^2^, repeated resting blood pressure < 180/95 mmHg, prostate specific antigen (PSA) ≤ 4.1 ng/mL, serum haematocrit ≤ 50%, alanine aminotransferase less than three times the upper limit of normal, and serum creatinine < 2 mg/dL. | Community | USA |
| Grip strength,  measured using a  Smedley-type dynamometer (T.K.K.5401, TAKEI Scientifc  Instruments Co., Ltd., Niigata, Japan). | Suzuki, 2019  ***Note***: 1 other tool assessed, a Knee extension strength tool | Validation (absolute reliability) | Not reported | 718 | Men: 73.4 ± 5.3  Women: 71.2 ± 4.5 | 72.56 | Age 65 years and older, and able to perform activities of daily living (ADL) independently. | Community-dwelling | Japan |
| Knee extension strength,  measured using a handheld dynamometer (μ-Tas F-1; Anima Inc.,  Tokyo, Japan). | Suzuki, 2019  ***Note***: 1 other tool assessed, a Grip strength tool | Validation (absolute reliability) | Not reported | 718 | Men: 73.4 ± 5.3  Women: 71.2 ± 4.5 | 72.56 | Age 65 years and older, and able to perform activities of daily living (ADL) independently. | Community-dwelling | Japan |
| The 30-s Chair-Stand Test | Jones, 1999  ***Muscle group***:  lower body strength | Validation (test-retest reliability and the criterion-related and construct validity) | Not reported | 76 | 70.5 ± 5.5 | 55.30 | Participants were over the age of 60 years, community-residing, functionally independent, ambulatory, and did not suffer lower extremity pain, unstable cardiovascular disease, or any other medical condition that would be contraindicated for maximal strength testing of the lower extremity according to American College of Sports Medicine guidelines. | Community | Authors’ country: USA |

**Appendix 2.c**: Characteristics of the included studies on muscle power assessment tools

| **Instrument** | **Study Reference** | **Type of study**  (As stated in the objectives) | **Study design** | **N** | **Age, years**  Mean ± SD | **% Female** | **Inclusion criteria** | **Setting** | **Country** |
| --- | --- | --- | --- | --- | --- | --- | --- | --- | --- |
| The 30-s sit-to-stand (STS) muscle  power test | Alcazar, 2020 | Validation (validity) | Cross-sectional study | 628 | 72.4±7.7 | 55.10 | Older people (≥60 years old) participating in the Copenhagen Sarcopenia Study, a population-based cross-sectional study that included men and women aged 20–93 years living in the Copenhagen metropolitan area. | Community | Denmark |
| The sit-to-stand (STS) muscle power test | Alcazar, 2018 | Validation (validity) | Not reported | 40 | 77.6 ± 5.4 | 60.00 | Participants recruited through advertisements and community newsletters and screened if they were aged ≥70 years. | Community | Spain |
| The sit-to-stand power test (STSp), using a portable linear transducer | Balachandran, 2021 | Validation (construct validity,  reliability, and measurement error) | Not reported | Validity: n = 51  Reliability:  n = 36 | Validity: 71.3 ± 5.7  Reliability:  70.4 ± 5.4 | Validity: 62.70  Reliability: 47.20 | Older than 65 years of age, live independently in the community, and be able to communicate in English. | Community | Authors’ country: USA |
| The Vertical Jump (VJ) measured by a contact mat | Farias, 2013 | Validation (reliability) | Not reported | 31 | 69.5 ± 5.6 | 100.00 | Age ≥ 60 years and regular physical exercise. | Elderly Living Center | Brazil |
| The Tendo Weightlifting Analyzer (Trencin, Slovak Republic) | Gray, 2014 | Validation (validity and reliability) | Cross-sectional design | 20 | 71.6 ± 5.6 | 60.00 | Community-dwelling older adults (> 65 yrs). | Community-dwelling | USA |
| The Counter-movement jump (CMJ) test performed on a force platform (Kistler Instruments 9281 B, Winterthur, Switzerland, 40 x 60 cm) | Holsgaard Larsen, 2007  ***Note***: also assessed another tool for Stength only) | Validation (test–retest reproducibility) | Not reported | 18 | 72.3 ± 6.6 | 100.00 | Subjects participating in multi-component activities once per week, considered as moderately trained. … None of the participants had a history of orthopedic or neurological disorders and did not report any bone fractures in the lower extremities within the last 5 years. | Community-dwelling | Authors’ country: Denmark |
| The chair stand mean power (CSMP) test, using the Fitro Dyne device (Fitronic S. R. O. Co, Slovakia). | Kato, 2015 | Validation (reproducibility) | Not reported | 87  IG: n = 48  DG: n =  39 | Needed no assistance (IG: 78.7 ± 4.6)  Needed assistance (DG: 80.2 ± 4.8) | 100.00 | Women who received permission to participate from their physicians were divided into 2 groups: an independent group (IG) who needed no assistance, and a dependent group (DG) who were beneficiaries of long-term care insurance and needed assistance to perform ADL. | Community-Dwelling | Authors’ country: Japan |
| The sit-to-stand  (STS) performance power using a linear encoder (MuscleLab Power model MLPRO, Ergotest Technology, Langesund, Norway) | Lindemann, 2015 | Validation (construct validity) | Cross-sectional study | 88 | 78.0 ± 5.7 | 100.00 | Only women included, in order to avoid a possible sex effect. Other inclusion criteria were: age ≥ 70 years, living in the community (both criteria preselected by the health insurance company), and no cognitive impairment. | Community | Authors’ country: Germany |
| The Jumping Mechanography | Rittweger, 2004 | Validation (reproducibility) | Not reported | 36 | 60.8 ± 19.0 | 61,10 | Physically Competent Adult and Elderly Subjects. | Geriatric clinic | Germany |
| A standardized heel-rise test  (Using trunk accelerometry)  (For vertical ground reaction ***force*** and external mechanical ***power***) | Schmid, 2011  ***Note***: same tool used to assess muscle strength and power | Validation (intrasession reliability; concurrent validity) | Cross-sectional study | 54 | 81.2 ± 6.4 | 74,07 | A minimum age of 65 years and the ability to perform at least one of the following daily-living activities without assistance: standing up from a normal chair, walking 10 meters, and climbing up and down a stair with six steps. | Retirement homes | Switzerland |
| Unilateral leg extension power (W) using the Bassey Power Rig (University of Nottingham, Nottingham, U.K.) | Schroeder, 2007  ***Note***: 1 other tool for muscle strength assessed in this study | Validation (Reliability) | Data from 3 RCTs, forming 2 cohorts (*Cohort 1* & *Cohort 2*) | n = 116  *Cohort 1* (n = 90)  *Cohort 2* (n = 26) | *Cohort 1:* 72.0 ± 5.0  *Cohort 2:* 69.0 ± 3.0 | 0% | Participants must not have participated in regular resistance training, physical activity (with the exception of a walking program), or competitive sports for the previous 6 months. Participants had to have a body mass index (BMI) ≤ 35 kg/m^2^, repeated resting blood pressure < 180/95 mmHg, prostate specific antigen (PSA) ≤ 4.1 ng/mL, serum haematocrit ≤ 50%, alanine aminotransferase less than three times the upper limit of normal, and serum creatinine < 2 mg/dL. | Community | USA |
| The Ramp Power Test | Signorile, 2007 | Validation (validity and reliability) | Not reported | Total: 520  Validity: n = 59  Reliability: n = 59 | Validity:  73.2 ± 7.3  Reliability: 73.8 ± 6.1 | Validity: 71.20  Reliability: 64.40 | Participants presenting a comprehensive health evaluation from their doctor certifying that they had no acute systemic illnesses or conditions that would disqualify them from participating in an exercise study. | Community-dwelling | USA |

USA = United States of America

**Appendix 2.d**: Characteristics of the included studies on endurance assessment tools

| **Instrument** | **Study Reference** | **Type of study**  (As stated in the objectives) | **Study design** | **N** | **Age, years**  Mean ± SD | **% Female** | **Inclusion criteria** | **Setting** | **Country** |
| --- | --- | --- | --- | --- | --- | --- | --- | --- | --- |
| The 6-Minute Walk Test | Rikli, 1998 | Validation (Test-retest reliability; convergent validity; ability to discriminate) | Not reported | 77 | 73.1 ± 7.2 | 62.3 | Participants had to be over age 60, community residing, functionally independent, and ambulatory (without the use of assistive devices); they could have no medical conditions that would contraindicate submaximal testing according to ACSM (American College of Sports Medicine) guidelines. | Community | Authors’ country: USA |
