# Supplementary material for: Measures of attributes of locomotor capacity in older people: a systematic literature review following the COSMIN methodology
Source: Age Ageing. 2023 Oct 30;52(Suppl 4):iv44–66. doi: 10.1093/ageing/afad139 (PMC10615073; doi:10.1093/ageing/afad139)
Supplement: aa-23-0360-File004_afad139 [file aa-23-0360-file004_afad139.docx]

World Health Organisation: *Measurements of Healthy Ageing*

**Measures of attributes of locomotor capacity in older people: A systematic literature review following the COSMIN methodology**

**SUPPLEMENTARY DATA**

**Appendix 3: Characteristics of the included tools**

| **Instrument** | **Construct or attribute measured** | **Details about attributes measured** | **Mode of administration** | **(Sub)scale (s) (number of items),**  (if applicable) | **Range of scores/scoring**  (if applicable) | **Original language** /**Translations**  (if applicable) |
| --- | --- | --- | --- | --- | --- | --- |
| The 6-Minute Walk Test | Endurance | Physical Endurance, defined as the ability to maintain submaximal aerobic exercise for an extended time.  The 6-min walk is a time-constant walking test. | Clinician report | NA | The score is the total yards walked, determined by having an observer record a hash mark on the score card for each completed lap, then multiplying the total number of completed laps by 50 and adding the additional yards covered. | NA |
|  |  |  |  |  |  |  |
| The Stepping Threshold Test (STT) | Balance | Reactive balance  Using a new strategy developed to evaluate the step and stepping behavior of the STT, which is called the  ‘direction-sensitive evaluation’ (DSE). | Clinician report | NA |  | NA |
| Unstable board  (DYJOC BOARD, SAKAI Medical Co., Ltd.) | Balance | Dynamic balance  a new method to measure dynamic balance, which mimics the Biodex balance  system but uses relatively inexpensive equipment that is highly portable. | Clinician report | NA |  | NA |
| The Balance Evaluation Systems Test  (BESTest) | Balance | Static and dynamic balance  (6 subsystems: biomechanical constraints, stability  limits/verticality, anticipatory postural adjustments, postural  responses, sensory orientation, and stability in gait) | Clinician report | 36 items organized into 6 subsections. | Each item is scored from 0 (severe balance impairment) to 3 (no balance impairment), and the maximum possible total score is 108 points. | **Original language**: English  **Translations:**   - Spanish |
| The Mini-Balance Evaluation Systems Test  (Mini-BESTest) | Balance | Static and dynamic balance  This is a shortened version of the BESTest. This version was designed  to reduce the time burden while focusing on dynamic balance assessment. | Clinician report | Includes 14 items from sections of the BESTest.  Two of the 14 items are scored bilaterally. | Each item is scored from  0 (severe balance impairment) to 2 (no balance impairment), and  the maximum possible score is 28 points. Higher scores indicate  better balance performance. | **Original language**: English  **Translations:**   - Spanish |
| The Brief‐Balance Evaluation Systems Test (Brief‐BESTest) | Balance | Static and dynamic balance.  This in an abbreviated version of the BESTest. | Clinician report | 6-item balance test that contains 1 item from each of the 6 subsections of the BESTest. Two of the 6 items are scored bilaterally, resulting in an 8-item balance test. | Each item is scored from 0 (severe balance impairment) to 3 (no balance impairment), and the maximum possible score is 24 points. Higher scores indicate better balance performance. | **Original language**: English |
| The modified Clinical test of Sensory Interaction in Balance (mCTSIB) of the Balance Platform Biodex Balance System (BBS)  (Review authors’ Note: This is also an “Instrumented” mCTSIB (i-mCTSIB) | Balance | A dynamic platform  Test (A tool designed to assess postural stability).  “The Biodex Balance platform (BBS) is …  a circular platform that measures the degree of tilt in each axis rather than measuring the deviation of the CoP.” | objective assessment | NA |  | NA |
| The instrumented modified Clinical Test of Sensory Interaction on Balance (i-mCTSIB) utilizing the  Neurocom Very Simple Rehab (VSR) Sport force plate (Natus Medical  Incorporated, Pleasanton, California). | Balance | A tool designed to assess postural stability.  It tests static and  dynamic balance under static surface conditions only, with  the measurement device consisting of a static force plate.  “The VSR Sport is a computerized tool that utilizes a static force plate to assess postural stability by measuring postural sway.” | objective assessment | The i-mCTSIB test includes 4 separate test conditions including (1) eyes open, firm surface,  (2) eyes closed, firm surface, (3) eyes open, foam surface,  and (4) eyes closed, foam surface. | Sway velocity (°/s) measurements: mean sway velocity score for three 10-second trials in each condition and composite sway  velocity score that is averaged over the twelve 10-second trials. Sway velocity is measured as the distance traveled by the COP (expressed in degrees) per unit of time (second)  for the trial. | NA |
| The Berg Balance Scale (BBS) | Balance | Static and dynamic balance abilities. | Clinician report | 14 items common in everyday life | All items are graded on a five-point scale, 0 (impossible) to 4 (normal function). Points are based on the time the position can be maintained, the distance the arm is able to reach forward, or the time to complete the task. The maximum score is 56 points. | **Original language:**  English  **Translations:**   - Brazilian-Portuguese - Turkish Version - Persian version |
| The Lateral Reach Test | Balance | “a clinical test to reflect the ability to move to the lateral limits of stability.” | Clinician report | NA |  | NA |
| The Functional reach (FR) test | Balance | Standing balance  “Functional reach is a test of balance that  measures the furthest distance a person can reach forward  while standing and not taking a step.” | Clinician report | NA |  | NA |
| The Six-Spot Step Test | Balance | Dynamic balance  “The Six-Spot Step Test is a timed measure of the  participants’ ability to maintain balance while challenging their stability while walking.”  “When applying the conceptual understanding of balance as proposed by Fay Horak, the Six-Spot Step Test covers and challenges physiological domains of the balance control system related to anticipatory postural adjustments and stability in gait, and to a lesser  degree of biomechanical constraints, stability limits/verticality, and sensory orientation.” | Clinician report | NA |  | NA |
| Gait Initiation Assessment | Balance | Dynamic balance  “Measurements of center of gravity-center of pressure separation (CG-CP moment arm)  during gait initiation.”  “Peak CG-CP moment arm measures how far the subject will tolerate the whole-body CG to deviate from the ground reaction force’s CP; it represents dynamic balance control.” | objective assessment | NA |  | NA |
| The Wii Fit Balance Board  (Wii Fit® platform,  Nintendo, Japan) | Balance | Dynamic balance  “… we designed a  balance assessment device based on the Wii Fit balance board. This real-time device comprises the balance board, software, and database. It can show and calculate the distance of weight shifting.” | objective assessment | NA |  | NA |
| The limits of stability (LOS) test | Balance | Dynamic Balance | objective assessment | NA |  | NA |
| The Four Square Step Test (FSST) | Balance | A clinical test of dynamic standing balance.  The FSST is a performance-based balance tool involving stepping over four single-point canes placed on the floor in a cross configuration.  The FSST was originally validated in 2002 as a fall risk tool and balance assessment measure for older adults. | objective assessment | NA |  | NA |
| The mediolateral balance assessment (MELBA) tool | Balance | Mediolateral (ML) balance: balance in the mediolateral direction.  A tool in which subjects track a visually presented target with ML movements of their Center of Mass (CoM) | objective assessment | NA |  | NA |
| The Spring Scale Test (SST) | Balance | The spring scale test (SST) is ***a standing test of reactive and proactive balance*** that assesses the maximum waist-pull perturbation force against which a person can maintain postural stability against both anterior and posterior direction perturbations. | objective assessment | NA |  | NA |
| The Microsoft Xbox One Kinect (Kinect v2) | Balance | Static and Dynamic Balance.  “The Kinect v2 is a videogame accessory that employs video and infrared cameras to create a three-dimensional (3D) representation of the space, thus combining a video cameras’ cost effectiveness and the ease of use with the analysis capabilities of 3D motion analysis systems.” | objective assessment | NA |  | NA |
| The TURN 180 test | Balance | The test is devised to measure *dynamic balance* at the impairment level especially among people who may be afraid of falling. | Clinician report | NA | The number of  steps taken by the subjects to complete the Turn 180 Test is noted. | NA |
| The Lower Quarter Y-Balance Test (LQ-YBT) | Balance | A measure of dynamic balance  The LQ-YBT was completed using the wooden Professional Y Balance Test KitTM (Move2Perform, LLC). | objective assessment | NA |  | NA |
| The Narrow Path Walking Test (NPWT) | Balance | The Narrow Path Walking Test was developed to challenge one’s balance when walking in a narrowed pathway | Clinician report | NA |  | NA |
| One leg standing (OLS) | Balance | Defined as the number of seconds each subject was able to maintain balance on one foot, with a possible range of 0–30 s. | Clinician report | NA |  | NA |
| Tandem Gait (TG) | Balance | Defined as the number of steps taken in a heel-to-toe fashion before deviating from a taped line on the floor, with a range of steps from 0 to 10. | Clinician report | NA |  | NA |
| The five-times-sit-to-stand test (FTSST) | Balance | FTSST as a measure of dynamic balance | Clinician report | NA |  | NA |
| The Maximum Step Length (MSL) test | Balance | A measure of balance capabilities in older adults. | Clinician report | NA |  | NA |
| The Thirty-Rapid-Step test (30-RST) | Balance | A measure of dynamic balance | Clinician report | NA |  | NA |
| The Community Balance and  Mobility Scale (CBM) | Balance | Except for the unilateral stance, all items evaluate dynamic balance. | Clinician report | 13 items | Range of scoring: 0–5  (A score of 0 denotes the  inability to perform the task. Scores  1–5 correspond to better performance, with a maximum  score of 96 points possible.) | **Original language**: English  **Translations**: German |
| The Shortened version of the Community Balance and Mobility Scale (s-CBM) | Balance | Factor 1 represents balance with reduced base of support, whereas Factor 2 represents dynamic balance with an additional visual target. | Clinician report | 4 items, which  are all performed bilaterally  (“Unilateral Stance,” “Lateral Foot Scooting,” “Hopping Forward,” “Walk, Look and Carry”) | Range of scoring: 0–5  (a maximum score of 40 points is achievable on the s-CBM) | **Original language**: English |
| The Modified Version of the Community Balance and Mobility Scale (CBMS-Home) | Balance | The CBMS was modified by reducing the length of the walkway used for 4 items from 8 m to 4 m and modifying the scoring criteria for the 5-point scales for these items to accommodate this change. These 4 modified CBMS items using the 4-m walkway included “forward to backward walk”; “walk and look”; “walk, look, and carry”; and “run with controlled stop.” | Clinician report | 12-item CBMS-Home (8 original items and 4 modified items of the CBMS) | 5-point scales (The maximum score for CBMS-Home is 90  points.) | **Original language**: English |
| The “Step-Ex”  (New Development Technologies [NDT], Stockholm, Sweden) | Balance | A step-execution test: Step-Ex consists of two portable force platforms that register vertical ground reaction forces connected to a computer for easy detection of temporal events and phases (reaction-, preparation-, stepping- and step execution phase). | objective assessment | NA |  | NA |
| Tinetti's POMA balance subscale | Balance | Measures an individual's position changes and ability to balance while performing certain activities | Clinician report | 9 items | The total score on the POMA balance subscale can range from 0 to 16, with a higher score indicating better balance. | **Original language**: English |
| The Short Berg Balance Scale  (BBS-9) | Balance | Static and dynamic balance | Clinician report | 9 items | Total score range: 0-36 | **Original language**: English |
| The Multi-Directional Reach Test (MDRT) | Balance | The MDRT is a modification of the FRT and involves the individual reaching forward, backward, right, and left (measure of limits of stability in the anterior-posterior and medial-lateral  directions). The FRT and MDRT are  measures of balance while standing in place. | Clinician report | NA |  | NA |
| The Kinect system (Kinect for Xbox 360™, Microsoft Corp, Seattle, WA, USA) | Balance | Static and dynamic balance.  Uses depth-imaging camera to track the movement of the participant’s skeleton. Using this method, it is possible to measure velocity (V) in addition to the distance of forward reach. | Objective assessment | NA |  | NA |
| The Fullerton Advanced Balance (FAB) Scale | Balance | Static and dynamic balance.  “The FAB scale was developed in order to identify more subtle changes in the multiple dimensions of balance (e.g., motor, sensory, musculoskeletal) among higher-functioning active older adults. | Clinician report | 10 test items | Each test item is scored using a 0–4 scale. The highest score that can be obtained is 40 points, and the lowest is zero. Higher scores indicate better balance abilities. | **Original language**:  English  **Translations**:   - Turkish version (FAB-T) |
| The short form of the Fullerton Advanced Balance (SF-FAB) scale | Balance | Provides information about sensory and motor aspects of static and dynamic balance. | Clinician report | 4 items: step up and over a 6-in. bench; tandem walking; stand on one leg; stand on a foam with eyes closed. | Each item is scored using a 0 (unable/unwilling to perform the test item) to 4 (correctly performs the test item) Likert scale. The total score  of the SF-FAB scale ranges from 0 to 16 with participants  achieving 9 points or less considered to be at high risk of falling. | **Original language**:  English |
| The parallel walk test | Balance | Dynamic balance.  “The parallel walk test was devised to measure the lateral movement during gait.” | Clinician report | NA | The scoring was based on +1 for foot placement on the line and +2 outside the line or grasping something to maintain balance (eg, wall or railing). A larger score denoted a worse performance. | NA |
| Timed Up and Go (TUG) | Balance | The purpose of this test is to assess physical mobility in terms of speed, agility, and dynamic balance. | Clinician report | NA | The test score corresponds to the time (in seconds)  elapsed from the "starting" signal until the participant sat in the chair. | NA |
| The Balance Computerized Adaptive Testing (Balance CAT) | Balance | Balance type not specified.  The Balance CAT is a computer-based instrument, and the program is installed on a web-based server (website: http://140. 112.116.44/cat/). | Clinician report | 34 | The total score of the Balance CAT ranges from 0 to 10, with a higher score representing better balance function. | **Original language**: English |
| The MyBalance test  (A tool for 2 different tests: Balance and muscle strength) | Balance | The MyBalance test measures body sway in static quiet standing and leg strength when rising from a chair.  Sensor measurements with the MyBalance prototype: Three Maximal Chair Stand Test (leg strength test), a modification of the traditional Five Times Sit-To-Stand test. | Self-test application (Objective measurement) | NA |  | NA |
| The Functional Gait Assessment (FGA) | Balance | The FGA is a tool that evaluates the individual’s postural stability during tasks that require change in gait patterns. In other words, it is a clinical outcome measure that assesses balance while walking. | Clinician report | 10-items | Graded on a four-point ordinal scale ranging from 0 (severe impairment), 1 (moderate impairment), 2 (mild impairment), to 3 (normal). Maximum score of 30 points. The higher the score, the better the individual’s postural stability during gait tasks. | **Original language**:  English  **Translations**:  Brazilian Portuguese (The FGA-Brazil) |
| The ‘‘Get-up and Go’’ Test | Balance | Dynamic balance  The “‘Get-up and Go test’’ requires patients to stand up from a chair, walk a short distance, turn around, return, and sit down again. | Clinician report |  | Five-point scale:  1=normal; 2=very slightly abnormal; 3 = mildly abnormal; 4=moderately abnormal; 5=severely abnormal. | NA |
| The apparatus for assessment of postural responses | Balance | A center-of-pressure (COP) based balance measure.  This is an objective balance assessment method for purposes of telemonitoring and telerehabilitation in elderly population. | Objective assessment | NA |  | NA |
| A comprehensive set of inertial sensor measures of postural sway  (The Balance Score (BS) & The Weighted Balance Score (WBS)) | Balance | Static balance assessment using inertial sensor-derived measures.  Sensor Data Acquisition and Processing: Using a single body-worn inertial sensor, containing both a tri-axial accelerometer and gyroscope (SHIMMERTM, Shimmer Research, Dublin, Ireland) | Objective assessment  (could allow for older adults to perform a directed self-assessment in the home.) | NA |  | NA |
| The Pavia  Instrumented Tinetti Test (PITT) | Balance | The PITT measures the participant’s movement during the execution of the items of the Tinetti balance scale (Tinetti, 1986), and computes performance indicators from these measurements. | objective evaluation | 6 manoeuvres are considered | A global performance score (PTOT), obtained from the sum of all the standardized values. | NA |
| Dynamic Gait Index  (DGI) | Balance | The test measures the dynamic balance abilities of  participants under eight different functional gait  demands. | Clinician report | 8 items | Each item is  scored from 0 to 3, where lower scores indicate more  severe impairments, and a total score ranges from 0 to 24. Higher total scores denote greater functional walking abilities. | NA  (English assumed)  **Versions**:  Danish Version |
| The NIH Toolbox^®^ Standing Balance Test | Balance | Assessment of standing balance.  As per the protocol, the NIH Toolbox®  app was set up on an iPad while an iPod was secured to each participant anteriorly between the iliac crests using a gait belt. Data were wirelessly transmitted from the iPod to the iPad via a Bluetooth connection. | Objective assessment. | NA | Item response models are used to derive the Theta score representing the participants’ balance.  Balance dysfunction is determined if a fully corrected Theta score is 2 standard deviations below the norm  reported by the NIH. | NA |
| The Biodex SD (Biodex Medical Systems, Shirley NY) | Balance | A commercially available  stabilometer that records center of pressure (COP) data at a 40 Hz sampling frequency. | objective assessment | NA | Average medial–lateral COP velocity was computed from the COP data. | NA |
| The Balance Scale (by Roberts) | Balance | This scale consisted of eight stances: bipedal stance with eyes open and closed, monopedal stance with eyes open and closed and these four stances repeated on a beam. These stances reflect the two factors related to balance, base of support and visual cues. | Clinician report | This scale consisted of eight stances | The ability of subjects to maintain these stances was timed for a maximum of 30 sec. The score for each stance was the time in seconds the subject maintained the stance. | NA |
| The Nintendo Wii Fit *exergame* | Balance | Used to assess postural sway through measurement of the center of pressure (COP).  A Wii Balance Board (WBB) was used in conjunction with dedicated software such as Wii Fit (Nintendo Co., Ltd,  Kyoto, Japan) to challenge the user’s balance ability through games (also known as *exergames*) with different levels of difficulties. | objective assessment | Three balance activities were selected for COP assessment: a.) Snowboard slalom; b.) Ski slalom; c.) Balance bubble | Time scores for ski slalom and snowboard slalom, and distance scores for balance bubble. | NA |
| The Wii Balance Board™ (WBB) | Balance | For assessment of center of pressure (CoP) motions during standing balance trials: A tandem test is performed on a Wii BB (Nintendo, Kyoto, Japan). | objective assessment | NA | The distance travelled by the CoP is the main outcome measure, and  was chosen because it takes into account the displacement in both mediolateral and anteroposterior directions. | NA |
| The Wii Stillness (WST) Test | Balance | A test for assessing static balance performance.  A pre-programmed balance assessment test as part of Nintendo’s Wii Fit exergame.  This test has the participant stand on the WBB as still as possible for 30-seconds. | objective assessment | NA | A stillness test score (ST) was given by the WST to indicate balance performance. Scores were given as a percentage from 0-100%. Scores closer to 0 indicating poorer balance performance and scores closer to 100% better balance performance. | NA |
| The 'balance meter' | Balance | A system which may be used to monitor lateral and anteroposterior sway. | Objective measure | NA | The index used as a measurement of postural sway is the root mean square (RMS) of the centre of pressure (COP). RMS is the displacement of the COP relative to the mean COP, over a defined period of time.  RMS COP displacement relative to the mean COP is an amplitude-based measurement. This is the parameter measured by the balance meter. | NA |
| The AMTI Accusway system for balance and postural sway  measurement (Advanced Mechanical Technology, Inc.,  Watertown, Massachusetts) | Balance | The postural balance measurements were collected under two task conditions: standing quiet (without a secondary cognitive task) and standing quiet combined with counting backwards in steps of seven. Both tasks were measured with and without vision. | Objective measure | NA | The system measures ground reacting force and  moments in 3 orthogonal directions with a sampling frequency of 50 Hz. These provide the COP coordinates,  which enables the calculation of the maximum displacement in the anterior-posterior and medial-lateral direction (Max-AP; Max-ML), the root-mean-square amplitude in anterior-posterior and medial-lateral direction from the centroid in x- and y-axis (RMS-AP; RMS-ML), the mean velocity (MV) and the area of the 95th percentile ellipse (AoE). | NA |
| A dual-task computer game-based platform (TGP) | Balance | The TGP integrates head tracking and cognitive tasks with balance activities. | objective assessment | NA | Balance performance measures under altered sensory and cognitive conditions: The root mean squared (RMS) ML-COP and AP-COP excursions dimensions were computed for each task. Increases in RMS COP were interpreted as decreases in stability. | NA |
| The Modified Bathroom Scale | Balance | A modified bathroom scale that enables elderly people to measure their own balance by stepping onto the scale.  The modified bathroom scale uses the signals from four force sensors located in the corners of the scale to collect information regarding various parameters. | objective assessment | NA | An overall balance indicator is calculated using the information from four parameters: step on delay, rise rate, surface under the stabilogram, and the average velocity of the trajectory. Each parameter is scored on a scale from 0 to 4, which results in an overall balance score between 0 and 16. A higher score indicates better balance. | NA |
| Models for estimating decline in balance using accelerometry-based gait features | Balance | Inertial sensor-based gait analysis (a novel method for using wearable accelerometers to detect early signs of deficits in balance from gait). | objective assessment | NA | Altogether 43 features were extracted from the accelerometer signals, including Frequency features, Basic features, Temporal gait features, and Resultant acceleration amplitude features. Two control variables were included (Age and BMI). | NA |
| The FICSIT Balance Scales (FICSIT-3 and FICSIT-4) | Balance | Two simple balance scales comprising three or four familiar tests of static balance (A composite measure of static balance status based on ability to maintain balance over a diminishing base of support).  FICSIT (Frailty and Injuries: Cooperative Studies of Intervention Techniques) was a series of eight linked but independent trials carried out at Portland, New Haven (Yale), Seattle, Atlanta, Iowa, Farmington, San Antonio, and Boston. | Clinician report | FICSIT  used tests requiring subjects to maintain three different stance tests without assistive devices: parallel stance; semi-tandem stance; tandem stance.  A four-test version of the balance scale was defined (the FICSIT-4 balance scale), using one-leg balance times in addition to the three measures. | The time for which each stance could be maintained (1-10 seconds) was measured with a stopwatch. | NA |
| The Balance Tracking System (BTrackS) | Balance | Tool used to collect medial-lateral (COP_x_) and anterior-posterior (COP_y_) data (in eyes open (EO) and eyes closed (EC) static balance conditions).  BTrackS collects COP postural sway data in a similar manner to the force plate (FP) with individuals standing on the  BTrackS in a variety of balance conditions. | Objective assessment | NA | BTrackS uses customized software executed in LabVIEW to provide values for EO and EC balance conditions.  COP distance (in cm) traveled in each balance condition was used. | NA |
| The NeuroCom Smart Equitest Research System (Natus Medical Inc, Pleasanton,  California) | Balance | A force platform system that offers established standardized tests for assessment of postural control and different elements of the balance system: The limits of stability (LOS) test; The motor control test (MCT); and the sensory organization test (SOT). | Objective assessment | NA |  | NA |
|  |  |  |  |  |  |  |
| JAMAR hand-held hydraulic dynamometers  (pressure, pinch, platform/anchor) | Muscle  Strength | Isometric muscle strength  of ***six muscle groups***: elbow flexion (EFS), trunk extension (TES), knee extension (KES), hip flexion (HFS),  pinch (PIS) and dominant hand grip (HGS). (Note: Not all these muscle groups in all studies) | objective assessment | NA |  | NA |
| Handheld Dynamometry (HHD): The Lafayette Manual Muscle Tester, Model # 01163,  (Lafayette Instrument Inc.,  Lafayette, Indiana) | Muscle  Strength | Isometric muscle strength,  for muscle groups at the hip, knee, and ankle, incorporating different start positions (**three muscle groups**). | objective assessment | NA | Measurement of the peak force in kilograms during 5 s of muscle contraction. | NA |
| MicroFET2 hand-held dynamometer  (Hoggan Indiustries, Inc., West Jordan, UT, USA) | Muscle  Strength | To measure maximal isometric voluntary contraction eight different muscle groups: Knee extensors; Knee flexors; Hip abductors; Hip extensors; Ankle flexors; Ankle extensors; Elbow flexors; Elbow extensors. | objective assessment | NA |  | NA |
| A uni-axial load cell device  (Measurement Specialties XTC Series) | Muscle  Strength | Testing was limited to **three muscle groups**, i.e. knee extensors, hip abductors, and ankle plantarflexors. | objective assessment | NA |  | NA |
| The Nintendo Wii Balance Board (WBB) | Muscle  Strength | Isometric muscle strength  (Isometric hand grip strength; isometric whole-lower limb strength) | objective assessment | NA | For use in isometric whole-lower limb strength testing, a custom software recorded the isometric force-time curve from the lower extremities during a 20-second period and kilogram was the unit of measurement. | NA |
| The Modified Sphygmomanometer Test (MST) | Muscle  Strength | “This test uses the aneroid sphygmomanometer, a commonly acquired equipment by healthcare professionals for the assessment of blood pressure. This instrument is portable, has a low-cost and  provides an objective measurement.”  In Brito 2022, “seven muscles groups of the lower limb (bag method) and the grip strength (bag and non-adapted methods)” were tested.  Muscle groups:  Hip flexors; Hip extensors; Hip abductors; Ankle dorsiflexors; Ankle plantar flexors; Knee extensors; Knee flexors; Grip Strength (bag method); Grip Strength (non-adapted method). | objective measurement | NA | NA | NA |
| The Q Force | Muscle  Strength | A tool developed to measure isometric muscle strength of the Quadriceps muscle in different joint angles. | objective assessment | NA |  | NA |
| The calf-raise senior (CRS) test | Muscle strength | Strength and power in plantar flexor muscles | objective assessment | NA |  | NA |
| The isometric knee extension (IKE) test | Muscle strength | “a complement to the handgrip, which informs about the neural and muscular capability of knee extensor elements” | objective assessment | NA |  | NA |
| An analog dynamometer (SENSIX®, Poitiers, France) coupled with the DELSYS System (Trigno sensor, DELSYS, INC Boston; MA)  (A dynamometer fixed to a custom-made frame for a hip abductor and  Adductor test) | Muscle strength | Frontal plane hip muscles (abductors / adductors).  Hip abductor and adductor maximum voluntary isometric strength (MVIS) and rate of force generation (RFG) test (using a dynamometer fixed to a custom-made frame) | objective assessment | NA |  | NA |
| The Biodex System 3 isokinetic dynamometer  (Biodex Medical Systems, Shirley, N.Y., USA) | Muscle strength | Multi-Joint isokinetic strength-testing system. | objective assessment | NA | The Biodex System 3 Advantage software calculate the highest peak torque, the average peak torque and average power for each movement and speed combination. Force measured in Newton-Meter (Nm). | NA |
| Isokinetic dynamometer (KinCom 500H, Chattecx Corp., Hixson, TN, USA) | Muscle strength | Single-joint isokinetic/isometric leg muscle strength: Thigh muscles (quadriceps and hamstring) strength & Maximal plantar flexor strength | objective assessment | NA |  | NA |
| The Leg Press Sled (LPS) | Muscle strength | To measure isometric leg strength. | objective assessment | NA |  | NA |
| The Microfet 2000 strain gauge portable dynamometer (PD) | Muscle strength | Ankle joint muscle strength (isometric strength): Strength of plantarflexors and dorsiflexors. | objective assessment | NA |  | NA |
| A load cell setup | Muscle strength | Isometric back extensor strength | objective assessment | NA |  | NA |
| The push-off test (POT) | Muscle strength | Isokinetic strength.  Assessment using a calibrated handgrip dynamometer (Model #5030J1, JAMAR, DMM Canada) | objective assessment | NA |  | NA |
| The functional multi-joint isokinetic dynamometer (concentric (CON) and eccentric (ECC)  strength) | Muscle strength | Isokinetic strength.  Maximal voluntary strength measures from CON pushing and ECC resisting trials were obtained using an isokinetic dynamometer with a custom linear motion dynamometer attachment (Wheel attachment, Humac NORM Isokinetic Dynamometer, CSMi, Stoughton, MA). | objective assessment | NA |  | NA |
| The MyBalance test  (A tool for 2 different tests: Balance and muscle strength) | Muscle strength | The MyBalance test measures body sway in static quiet standing and **leg strength** when rising from a chair. | Self-test application (Objective measurement) | NA |  | NA |
| The maximal isometric strength test of the trunk  (measured by a precalibrated digital loading cell connected to the MuscleLab software) | Muscle strength | Isometric strength of trunk extensors and flexors was measured by a precalibrated digital loading cell (Kyoto, 333 A, Hown Dong, South Korea), connected to the MuscleLab software (Ergotest Innovation, Porsgrunn, Norway). | objective assessment | NA |  | NA |
| The one-repetition maximum (1 RM) using elastic resistance bands test | Muscle strength | Elastic resistance  during shoulder flexion.  The elastic resistance band test was performed using Thera-Band elastic bands (Thera-Band, The Hygienic Corporation, USA). | objective assessment | NA |  | NA |
| The one repetition maximum (1 RM) using a muscle strength training device for the arm/  shoulder (Pull Down, Norway) | Muscle strength | Dynamic muscle strength, measured by one repetition maximum (1 RM), defined as the maximum weight a person can lift only once in a complete range of motion. | objective assessment | NA | In the Pull Down device, the peak load is provided at the end of the range of motion, 180° shoulder flexion. | NA |
| The one-repetition maximum (1 RM) performed on the Keiser A-300 pneumatic equipment (Keiser Corp., Fresno, CA) or on selectorized weight-stack resistance exercise machines (Cybex VR2; Cybex International Inc., Medway, MA). | Muscle strength | 1-RM strength of both upper and lower body muscle groups (bilateral leg press, leg extension, leg flexion, latissimus pull-down – lat pull –, and chest press). | objective assessment | NA | Force displayed as Newtons or kilograms.  The greatest 1-RM measured for each exercise during the two strength testing sessions was used as the value for Maximal voluntary muscle strength (MVMS). |  |
| The lateral step (LS) test  (For the Indirect Assessment of Hip Abductor Muscle Strength) | Muscle strength | For its execution, 4 fitting steps (one 10-cm step, two 20-cm steps, and one 5-cm step) were used. | Clinician report | NA | The final score of the test corresponds to the greatest height of the step that the participant is able to climb up and down independently, with a maximum score of 40 cm. | NA |
| Tandem Gait (TG)  (For the Indirect Assessment of Hip Abductor Muscle Strength) | Muscle strength | Defined as the number of steps taken in a heel-to-toe fashion before deviating from a taped line on the floor, with a range of steps from 0 to 10.  The test challenges dynamically (TG) the mediolateral stability. | Clinician report | NA | Mean value of the variables time of TG  execution (s) and percentage of errors in relation to the number of steps taken (%). | NA |
| Single-leg stance (SS) test  (For the Indirect Assessment of Hip Abductor Muscle Strength) | Muscle strength | The test challenges statically (SS) the mediolateral stability. | Clinician report | NA | The variable obtained was the time of  permanence on SS, with a maximum score of 30 seconds. | NA |
| The five-repetition sit-to-stand (STS) test | Muscle strength | Tests of functional muscle strength.  Subjects were asked to stand up and sit down five times as fast as possible without delay. | Clinician report | NA | Timing with a digital stopwatch  (Began with the command go and ceased when subjects  returned to sitting after their fifth full stand). | NA |
| A standardized heel-rise test  (Using trunk accelerometry).  (For vertical ground reaction ***force*** and external mechanical ***power***) | Muscle strength | Plantar flexors (PFs) performance (using trunk accelerometry).  For acquisition of the vertical acceleration (VAcc) data, a triaxial accelerometer (Model 317A, Noraxon U.S.A. Inc; Scottsdale, Arizona) was used. | objective assessment | NA | Peak force (Fmax) (body weight);  time to peak force (tFmax) (seconds) | NA |
| Grip strength,  measured using a  Smedley-type dynamometer (T.K.K.5401, TAKEI Scientifc  Instruments Co., Ltd., Niigata, Japan). | Muscle strength | Isometric muscle strength test.  Measurement of maximum voluntary grip strength. | objective assessment | NA | Measurement expressed in kgf (kilogram-force). | NA |
| Knee extension strength,  measured using a handheld dynamometer (μ-Tas F-1; Anima Inc.,  Tokyo, Japan). | Muscle strength | Used to assess isometric muscle strength for the quadriceps muscle | objective assessment | NA | Measurement expressed in kgf (kilogram-force). | NA |
| The 30-s Chair-Stand Test | Muscle strength | Lower body strength measured by a 30-s test trial. | Clinician report | NA | The score was the total number of stands executed correctly within 30-s (more than halfway up at the end of 30-s counted as a fullstand). Incorrectly executed stands were not counted. | NA |
|  |  |  |  |  |  |  |
| The 30-s sit-to-stand (STS) muscle  power test | Muscle  power | STS muscle power was calculated using the subjects’ body mass and height, chair height and the number of repetitions completed in the 30-s STS test. | Clinician report | NA |  | NA |
| The sit-to-stand (STS) muscle power test | Muscle  power | A STS muscle power equation.  Sit-to-stand evaluation: The subjects completed five timed STS repetitions on a standardized armless chair (0.49 m height). | Clinician report | NA | The STS test finished when the subjects sat on the chair after the fifth STS repetition, and the time needed to complete the task was recorded with a stopwatch to the nearest 0.01 s. | NA |
| The sit-to-stand power test (STSp), using a portable linear transducer | Muscle  power | lower body power  A chair and a linear transducer (Tendo Weightlifting Analyzer,  Trencin, Slovak Republic) were used to assess peak power. |  | NA |  | NA |
| The Vertical jump (VJ) measured by a contact mat | Muscle  power | Vertical jump (VJ) performed on a contact mat.  VJ is a relevant tool to estimate jump height, which is an indirect measure of muscle power of the lower limb. | objective assessment | NA |  | NA |
| The Tendo Weightlifting Analyzer (Trencin, Slovak Republic) | Muscle  power | A method of determining muscular power during a functional task. | objective assessment | NA |  | NA |
| Counter-movement jump (CMJ) test performed on a force platform (Kistler Instruments 9281 B, Winterthur, Switzerland, 40 x 60 cm) | Muscle  power | Mechanical muscle output during maximal force plate jumping employing stretch-shortening cycle movements. | objective assessment | NA |  | NA |
| The chair stand mean power (CSMP) test, using the Fitro Dyne device (Fitronic S. R. O. Co, Slovakia). | Muscle  power | The velocity during a single chair stand was measured using the Fitro Dyne device. Power (watts) was calculated as the product of body mass and velocity during the sit-to-stand movement. | objective assessment | NA |  | NA |
| The sit-to-stand  (STS) performance power using a linear encoder (MuscleLab Power model MLPRO, Ergotest Technology, Langesund, Norway) | Muscle  power | Velocity (m/seconds) was calculated by application software (MuscleLab 4010, V8.09, Ergotest Technology), multiplied by body weight (N), and the resulting power (W) was taken as the outcome parameter. | objective assessment | NA |  | NA |
| The Jumping Mechanography  (A test performed on a ground reaction  force platform with a personal computer and an integrated  analog-digital board and software (Novotec Medical,  Pforzheim, Germany)). | Muscle  power | Jumping was performed as counter-movement jump (i.e., brief squat before the jump) with freely moving arms. The instruction was to jump with the head and chest as high as possible. | objective assessment | NA | Instantaneous power is calculated as the product of force and velocity. | NA |
| A standardized heel-rise test  (Using trunk accelerometry).  (For vertical ground reaction ***force*** and external mechanical ***power***) | Muscle  power | Plantar flexors (PFs) performance (using trunk accelerometry).  For acquisition of the vertical acceleration (VAcc) data,  a triaxial accelerometer (Model 317A, Noraxon U.S.A. Inc; Scottsdale, Arizona) was used. | objective assessment | NA | Peak power (Pmax)  (watts/body weight);  Average power (Pmean) (watts/body weight) | NA |
| Unilateral leg extension power (W) using the Bassey Power Rig (University of Nottingham, Nottingham, U.K.) | Muscle  power | Leg extension power | objective assessment | NA | At least 10–  12 trials were performed until a plateau was reached and the  highest score achieved was recorded for leg power (in Watts). | NA |
| The Ramp Power Test | Muscle  power | Knee extensor and plantar flexor power.  A further modification of the ramp test using a standard access ramp commonly found in most facilities (1:12 rise/run) rather than the 30.5° or 35° ramps previously used. | objective assessment | NA | Power output (in Watts) computed using a specific formula based on the following variables: body mass in kg; vertical distance between pads in meters; time necessary to complete the task in seconds; and acceleration due to gravity. | NA |
