# Supplementary material for: Measures of attributes of locomotor capacity in older people: a systematic literature review following the COSMIN methodology
Source: Age Ageing. 2023 Oct 30;52(Suppl 4):iv44–66. doi: 10.1093/ageing/afad139 (PMC10615073; doi:10.1093/ageing/afad139)
Supplement: aa-23-0360-File005_afad139 [file aa-23-0360-file005_afad139.docx]

World Health Organisation: *Measurements of Healthy Ageing*

**Measures of attributes of locomotor capacity in older people: A systematic literature review following the COSMIN methodology**

**SUPPLEMENTARY DATA**

**Appendix 4: Measurement properties of included tools and quality (detailed results)**

- Appendix 4.a: Measurement properties of Balance assessment tools
- Appendix 4.b: Measurement properties of Muscle strength assessment tools
- Appendix 4.c: Measurement properties of Muscle power assessment tools
- Appendix 4.d: Measurement properties of Endurance assessment tools

**Appendix 4.a**: Measurement properties of Balance assessment tools

| **Instrument** | **Reference** | **Reliability** | | | **Validity** | |
| --- | --- | --- | --- | --- | --- | --- |
|  |  | ***Reliability*** | ***Measurement error*** | ***Internal consistency*** | ***Criterion validity*** | ***Hypothesis testing for construct validity*** |
| The Balance Evaluation Systems Test (BESTest) | Anson, 2019 [19] | Test-retest,  ICC = 0.86  (+) | MDC = 8.9  SEM = 3.2 | N/R | N/R | Identification of older adults who had previously fallen (**):  AUC = 0.53, 95% CI = 0.37-0.69.  “95% CI included 0.5: No discriminative ability. (-) |
|  | Marques, 2016 [20] | Inter-rater,  ICC = 0.86  Test-Retest,  ICC = 0.77  (+) | Inter-rater,  LoA = − 6.5 to 7.2  Test-Retest*,*  MDC = 9; SEM=3.3; LoA = − 10.1 to 6.9 | N/R | Against the BBS, r_S_ ≥ 0.83  (+) | 1) Ability to identify fall status (with/without history of falls) (**): p<0.007; AUC = 0.71; 95% CI: 0.60 - 0.82) (+)  2) Against scores of the ABC, Mini-Best, and Brief-Best (#):  ABC, r_S_ = 0.46 (+)  Mini-Best, and Brief-Best, r_S_ ≥ 0.83 (+) |
|  | O'Hoski, 2015 [21] | N/R | N/R | N/R | N/R | 1) Convergent validity, against the ABC: r = 0.67; Vs PASE: r = 0.39; Vs TUG: r = − 0.68 Vs SLS: r = 0.67 (p< 0.001). (+)  2) Discriminative ability (fallers vs non-fallers: p = 0.054. (+) |
|  | Viveiro, 2019 [22] | Inter-rater*,*  ICC = 0.994  Test-retest,  ICC = 0.945  (+) | SEM = 5.6  MDC = 15.6 | N/R | 1) Sensitivity = 0.833  Specificity = 0.613  2) Against the BBS (§): Good agreement demonstrated, PABAK ≥ 83.7%. (+) | 1) Ability to identify fall status (fallers vs non-fallers) (**): p=0.004 (+)  2) Agreement between the balance tests (#): Good agreement demonstrated, PABAK = 83.7% to 98.0%. (+) |
|  | Wang-Hsu, 2018 [23] | Inter-rater,  ICC = 0.97  Test-Retest,  ICC = 0.93  (+) | SEM = 3.0  MDC = 8.2 | N/R | N/R | N/R |
|  | Yingyongyudha, 2016 [24] | N/R | N/R | N/R | N/R | Comparison of balance scores  between the participants (with/without history of falls  (**): p< 0.05 (+) |
| **Pooled or summary result (overall rating)** | | ICC = 0.77−0.99 (+) | MIC not defined (?) | N/R | r ≥ 0.70  (+) | Hypotheses confirmed  (+) |
| The ***Spanish version*** of the BESTest (Spanish BESTest) | Dominguez-Olivan, 2020 [25] | Inter-rater,  ICC = 0.97 | SEM = 1.18  MDC = 3.27 | Total score, Cronbach α = 0.97; Sections I to VI: α ≥ 0.84 (?) | Against the BBS,  r = 0.43 (−) | Against the Mini-BESTest (#):  r = 0.65; p < 0.001. (+)  Vs FES-I: r = 0.90 |
| **Pooled or summary result (overall rating)** | | ICC = 0.97  (+) | MIC not defined (?) | Criteria not met  (?) | r < 0.70  (−) | r > 0.50  (+) |
| The Mini-Balance Evaluation Systems Test  (Mini-BESTest) | Anson, 2019 [19] | Test-retest,  ICC = 0.84  (+) | MDC = 4.0  SEM = 1.4 | N/R | N/R | Identification of older adults who had previously fallen (**): AUC = 0.54, 95% CI = 0.39-0.69. 95% CI included 0.5: No discriminative ability. (−) |
|  | Marques, 2016 [20] | Inter-rater,  ICC = 0.71  Test-Retest,  ICC = 0.73  (+) | Inter-rater,  LoA = −4.2 to 3.7  Test-Retest,  MDC = 3.8  SEM = 1.4  LoA = −4.2 to 3.3 | N/R | Vs BBS, r_S_ ≥ 0.83  (+) | 1) Ability to identify fall status (with/without history of falls) (**): P<0.007; AUC = 0.76; 95% CI: 0.66 − 0.86. (+)  2) Against scores of the ABC, Best, and Brief-Best (#)  ABC, r_S_ =0.57 (+)  Best, and Brief-Best, r_S_ ≥ 0.83 (+) |
|  | O'Hoski, 2015 [21] | N/R | N/R | N/R | N/R | 1) Convergent validity: Against the ABC: r = 0.62; PASE: r = 0.33 (p = 0.004); TUG: r = −0.66; SLS: r = 0.68  (p < 0.001 unless otherwise stated) (+)  2) Discriminative ability (fallers vs non-fallers): p = 0.17 (−) |
|  | Viveiro, 2019 [22] | Inter-rater,  ICC = 0.992  Test-retest,  ICC = 0.933  (+) | SEM = 1.8  MDC = 4.9 | N/R | 1) Sensitivity: 0.778  Specificity = 0.709  2) Against the BBS (§): Good agreement, PABAK ≥ 83.7%. (+) | 1) Ability to identify fall status (fallers vs non-fallers)  (**): p=0.007 (+)  2) Agreement between the balance tests (#): Good agreement, PABAK = 83.7% to 98.0%. (+) |
|  | Yingyongyudha, 2016 [24] | N/R | N/R | N/R | N/R | Comparison of balance scores  between the participants (with/without history of falls)  (**): p<0.05 (+) |
| **Pooled or summary result (overall rating)** | | ICC = 0.71−0.99  (+) | MIC not defined (?) | N/R | r ≥ 0.83  (+) | (±), for discriminative validity  (+), for convergent validity |
| The ***Spanish version*** of the Mini-BESTest (Spanish Mini-BESTest) | Dominguez-Olivan, 2020 [25] | Inter-rater,  ICC = 0.79  (+) | SEM = 0.74  MDC = 2.04 | Total score, Cronbach α = 0.79; Items 1-4: α ≥ 0.80 | Against the BBS, r = 0.18  (−) | Against the BESTest (#): r = 0.65; p < 0.001 (+)  Against FES-I: r = −0.18; p = 0.37 (+) |
| **Pooled or summary result (overall rating)** | | ICC = 0.79  (+) | MIC not defined (?) | Criteria not met  (?) | r = 0.18  (-) | Hypothesis confirmed  (+) |
| The modified Clinical test of Sensory Interaction in Balance (mCTSIB) of the Balance Platform Biodex Balance System (BBS) | Antoniadou, 2020 [26] | Test-retest,  ICC = 0.628  (−) | N/R | N/R | N/R | Against the Mini-BESTest-GR (#): r_S_ = −0.239 to −0.652  (+) |
| **Pooled or summary result (overall rating)** | | ICC = 0.628  (-) | N/R | N/R | N/R | r > 0.50  (+) |
| The Berg Balance Scale (BBS) | Berg, 1992a [27] | Inter-observer, ICC = 0.98  (+) | N/R | N/R | Against Laboratory measures of postural sway (Force plates), r = −0.46 to −0.67  (−) | 1) Against the Tinetti Sub-scale on balance (#), r = 0.91 (+); and TUG: r= −0.76 (+)  2) Discrimination amongst the individuals (walk with/without aides) (**): p<0.0001. (+) |
|  | Berg, 1992b [28] | N/R | N/R | N/R | 1) Against laboratory measures of postural sway (Force plates), satisfactory correlations (?)  2) Predictive (multiple falls vs no fall over 12 months), adjOR for prediction of multiple falls =0.90 (95%CI: 0.83-0.97) (+) | 1) Differentiation amongst known groups (use/non-use of walking aids) (**): Difference in scores (p<0.0001) (+)  2) Against clinical judgements of balance (#): Global ratings of caregivers (r= 0.47 to 0.61); Self-ratings of participants (r = 0.39 to 0.41) (+) |
|  | Bogle Thorbahn, 1996 [29] | Inter-rater,  r_S_ = 0.88  (+) | N/R | N/R | 1) Relationship with reported frequency of falls (‡), Relationship found. (?)  2) Sensitivity: 53%; Specificity: 92% − 96% | 1) Against the individual's self-perceived overall balance ability (#), no relationship found. (?)  2) Differentiation among known groups (use/non-use of assistive devices) (**): Relationship existed (p<0.001) (+) |
|  | Harada, 1995 [30] | N/R | N/R | N/R | Sensitivity: 84%  Specficity: 78%  (Both at a cut-off score of 48, by ROC analysis) (+) | N/R |
|  | Holbein-Jenny, 2005 [31] | Test-retest,  ICC = 0.77  Inter-rater,  ICC = 0.88  (+) | N/R | BBS and 4 separate MDRT directions, Cronbach’s α=0.80  (?) | N/R | Against 4 MDRT directions and ABC (#), Vs 4 MDRT, r = 0.53 to 0.78 (+)  Forward and backward MDRT directions (r = 0.78 and 0.77); lateral directions (r = 0.53 and 0.63)  Vs ABC, r = 0.50 (+) |
|  | Marques, 2016 [20] | Inter-rater,  ICC = 0.88 (+)  Test-retest,  ICC = 0.50 | Inter-rater,  LoA = −2.2 to 2  Test-Retest,  MDC = 4.6  SEM = 1.7  LoA = −4.6 to 3.2 | N/R | N/R | 1) Ability to identify fall status (with/without history of falls) (**): P<0.007; AUC = 0.78; 95% CI: 0.68 − 0.87. (+)  2) Against scores of the ABC, Mini-Best, and Brief-Best (#)  ABC, r_S_ =0.58 (+)  Mini-Best, and Brief-Best, r_S_ ≥ 0.83 (+) |
|  | Muir, 2008 [32] | N/R | N/R | N/R | 1) Predictive validity**,** “good discriminative ability to predict multiple falls, with ROC”. (?)  2) Sensitivity = 0.25  Specificity = 0.87 | N/R |
|  | Pelicioni, 2022 [33] | Inter-rater,  via telehealth,  ICC = 0.96  Intra-rater,  in person,  ICC = 0.82  (+) | N/R | N/R | N/R | Against Physiological Profile Assessment (PPA) fall risk score (#), r= −0.45 and −0.52 (+) |
|  | Viveiro, 2019 [22] | Inter-rater,  ICC = 0.993  Test-retest,  ICC = 0.886  (+) | SEM = 3.8  MDC = 10.5 | N/R | Sensitivity: 0.944  Specificity: 0.548 | 1) Ability to identify fall status (fallers vs non-fallers) (**), p=0.003 (+)  2) Agreement between the balance tests (#): Good agreement, PABAK = 83.7% to 98.0%. (+) |
|  | Wang, 2006 [34] | Inter-rater,  ICC = 0.87  (+) | N/R | Cronbach’s α=0.77  (?) | N/R | 1) Convergent validity: against the TUG, Spearman’s ρ = −0.53  Against usual gait speed, ρ= 0.46 (+)  2) Discriminant validity (older adults with/without mobility/IADL disability), p < 0.0001 (+) |
|  | Yingyongyudha, 2016 [24] | N/R | N/R | N/R | N/R | Comparison of balance scores  between the participants (with/without history of falls  (**): p<0.05 (+) |
| **Pooled or summary result (overall rating)** | | ICC = 0.77−0.99  (+) | MIC not defined (?) | Criteria not met  (?) | Indeterminate  (?) | Hypotheses confirmed  (+) |
| The ***Brazilian version*** of the Berg balance scale (Brazilian BBS) | Miyamoto, 2004 [35] | Intra-observer, ICC = 0.9917  Inter-observer, ICC = 0.9856 (+) | N/R | N/R | N/R | N/R |
| **Pooled or summary result (overall rating)** | | ICC ≥ 0.99  (+) | N/R | N/R | N/R | N/R |
| The Lateral Reach (LR) Test | Brauer, 1999 [36] | Test–retest,  ICC = 0.999 (+) | N/R | N/R | Against Laboratory measures (Dual force platform system) (§), r = 0.331  (−) | N/R |
| **Pooled or summary result (overall rating)** | | ICC = 0.999  (+) | N/R | N/R | r<0.70  (−) | N/R |
| The Six-Spot Step Test | Brincks, 2021 [37] | Test-retest, within-day, ICC=0.96  between-day: ICC=0.94  (+) | MDC, within-day: 17.5  between-day: 21.6  LoA, within-day: −12.6 to 18.7; between-day: −13.3 to 22.9 | N/R | N/R | Convergent validity,  Against the Mini-BESTest, r_S_= −0.62  Against the Dynamic Gait Index, r_S_= −0.55  (+) |
| **Pooled or summary result (overall rating)** | | ICC = 0.94−0.96  (+) | MIC not defined (?) | N/R | N/R | r > 0.50  (+) |
| The Functional reach (FR) test | Brooks, 2006 [38] | N/R | N/R | N/R | N/R | Construct validity (known groups of patients according to their ambulatory status): no significant difference across the 3 levels of ambulation (p=0.40). Similar findings if 2 categories considered (aid/no aid). (−) |
|  | Galhardas, 2020 [39] | Test-retest,  ICC = 0.85  (+) | SEM = 1.5  MDC = 4.0 | N/R | N/R | N/R |
|  | Giorgetti, 1998 [40] | Inter-rater,  ICC = 0.73  (+) | N/R | N/R | N/R | N/R |
|  | Lin, 2004 [41] | Test-Retest,  ICC ≥ 0.93  (+) | N/R | N/R | Predictive ability (Odds ratios estimated)**:** Falls: 1.00 (0.98–1.02)**;** ADL decline: 0.96 (0.94–0.97)**;** ADL improvement: 0.99 (0.97–1.02)  (?) | 1) Discriminant validity: Subjects who were older, had experienced a fall in the previous year, used a walking aid, and suffered more ADL disabilities reached a shorter distance for the FR (p=0.000) (+)  2) Convergent validity, Against the Tinetti Balance scores, r = 0.48 (−) |
| **Pooled or summary result (overall rating)** | | ICC ≥ 0.73  (+) | MIC not defined (?) | N/R | Indeterminate  (?) | (+), for discriminative validity  (−), for convergent validity |
| Gait Initiation Assessment | Chang, 1999 [42] | N/R | N/R | N/R | N/R | Discrimination: Validly discriminates groups with sensitivity and specificity ≥ 50%. Test values significantly greater in healthy elders than in disabled elders and vestibular hypofunction subjects (p<0.O1) (+) |
| **Pooled or summary result (overall rating)** | | N/R | N/R | N/R | N/R | Hypothesis confirmed  (+) |
| The modified Wii Fit balance board | Chang, 2013 [43] | Inter-rater,  ICC = 0.93 to 0.99  (+) | N/R | N/R | N/R | N/R |
| **Pooled or summary result (overall rating)** | | ICC ≥ 0.93  (+) | N/R | N/R | N/R | N/R |
| The Stepping Threshold Test (STT) | Adams, 2021 [44] | N/R | N/R | N/R | N/R | 1) Convergent validity, STT (DSE) vs Brief-BESTest, r_S_ = 0.388**;** STT (DSE) vs 8LBS, r_S_ = 0.246 (−)  2) Discriminative validity: The STT sum score and subscores (DSE) showed no significant differences between fallers and non-fallers (p=0.799). (−) |
| **Pooled or summary result (overall rating)** | | N/R | N/R | N/R | N/R | Hypotheses not confirmed  (−) |
| The Unstable board  (DYJOC BOARD, SAKAI Medical Co., Ltd.) | Akizuki, 2018 [45] | N/R | N/R | N/R | N/R | Relationship with existing dynamic balance indices (#):  Vs Mini-BESTest Total: r = −0.115 and −0.316; (−)  Vs Functional Reach Test (FRT),  r= 0.067 and 0.083 (−) |
| **Pooled or summary result (overall rating)** | | N/R | N/R | N/R | N/R | Hypothesis not confirmed  (−) |
| The limits of stability (LOS) test | Clark, 1997 [46] | Repeated measures, no significant differences in measurements across the three  testing days. (?) | SEM values range: For 75% LOS: 0.038 – 5.50  For 100% LOS: 0.041 – 4.92 | N/R | N/R | N/R |
| **Pooled or summary result (overall rating)** | | ICC not reported  (?) | MIC not defined (?) | N/R | N/R | N/R |
| The Four Square Step Test (FSST) | Cleary, 2017 [48] | N/R | N/R | N/R | 1) Against the BBS (§),  ρ = −0.74 (+)  2) Prediction of future falls (‡): At 3, 6 and 12 months: p = 0.000 (+) | 1) Against the Tinetti and TUG tests (#), Vs TUG, Spearman’s ρ **=** 0.89; Vs Tinetti, ρ = −0.60 (+)  2) Comparison of test scores between groups (**): non-fallers vs single fallers vs multiple fallers (p = 0.03). Use/non-use of any type of assistive devices (p = 0.003). (+) |
|  | Işik, 2015 [49] | Test-Retest, Cronbach α = 0.96  (?) | N/R | N/R | Against the BBS (§),  r=−0.641 (−) | Relation between FSST and other balance tests (#):  Vs Single Leg Stance Test:  r=−0.348 (−)  Vs FRT: r =−0.232 (−)  *Vs* Timed Up and Go Test:  r = 0.595 (+) |
|  | Dite, 2002 [47] | Inter-rater,  ICC = 0.99  Test-retest,  ICC = 0.98  (+) | N/R | N/R | Sensitivity: 89%  Specificity: 85%  (Optimal cut-off score:15 seconds)  (+) | 1) Differences between multiple fallers, non-multiple fallers, and healthy comparisons, on the FSST (**): Significant differences were found (p<0.001). (+)  2) Against Balance and Mobility Measures (#): Vs TUG, r_S_ = 0.88 (+)  Vs Functional Reach Test (FRT),  r_S_ = − 0.47 (−) |
| **Pooled or summary result (overall rating)** | | ICC ≥ 0.98  (+) | N/R | N/R | Inconsistent  (±) | (±), for convergent validity  (+), for discriminative validity |
| The mediolateral balance assessment (MELBA) tool | Cofré Lizama, 2015 [50] | N/R | N/R | N/R | Predictive ability regarding gait stability (‡),  r=−0.48 and r=−0.57 (−) | N/R |
| **Pooled or summary result (overall rating)** | | N/R | N/R | N/R | r < 0.70  (−) | N/R |
| The Spring Scale Test (SST) | DePasquale, 2009 [51] | Test-retest,  ICC = 0.94  (+) | Absolute error: Method error (ME) = 0.74  (?) | N/R | N/R | 1) Convergent validity: Vs Timed Up & Go (r=−0.67); Vs Single limb stance (r=0.54); Vs Tandem stance (r= 0.55) (+)  2) Known groups validity (distinction between subjects with/without a history of  falling), p = 0.001. (+) |
| **Pooled or summary result (overall rating)** | | ICC = 0.94  (+) | MIC not defined (?) | N/R | N/R | Hypotheses confirmed  (+) |
| The Microsoft Xbox One Kinect (Kinect v2) | Eltoukhy, 2018 [52] | Relative consistency between systems, ICC > 0.75 | Absolute agreement,  ICC > 0.75  (?) | N/R | Against a gold standard 3D motion analysis system (§): Concordance between systems ranged from poor to nearly perfect. (?) | N/R |
| **Pooled or summary result (overall rating)** | | ICC > 0.75  (+) | MIC not defined (?) | N/R | r not reported  (?) | N/R |
| The TURN 180 test | Fitzpatrick, 2005 [53] | Within-observer consistency,  ICC = 0.828 (+) | LoA, −1.04 to 1.56  (?) | N/R | N/R | Comparison of mean step counts between fallers vs non-fallers (**): p = 0.008 (+) |
|  | Ranji, 2020 [54] | N/R | N/R | N/R | Against the BBS (§),  The two-tailed P-value is <0.0001 (r not reported) | N/R |
| **Pooled or summary result (overall rating)** | | ICC = 0.828  (+) | MIC not defined (?) | N/R | r not reported  (?) | Hypothesis confirmed  (+) |
| The Lower Quarter Y-Balance Test (LQ-YBT) | Freund, 2019 [55] | Inter-rater,  ICC ≥ 0.997 (right, left)  Test-retest,  ICC ≥ 0.978 (right, left)  (+) | Inter-rater, SEM ≤ 1.46, MDC ≤ 4.04  Test-retest, SEM ≤ 3.95, MDC ≤ 10.94  (?) | N/R | N/R | LQ-YBT Composite against other balance tests (#): Vs Same side single leg stance, r= 0.412 and 0.448. (−) |
| **Pooled or summary result (overall rating)** | | ICC ≥ 0.98  (+) | MIC not defined (?) | N/R | N/R | Hypothesis not confirmed  (−) |
| The Narrow Path Walking Test (NPWT) | Gimmon, 2013  [56] | Relative reliability, ICC = 0.77 to 0.92  (+) | SEM = 0.24 to 0.78, and 0.03 to 1.12  SRD = 0.10 to 1.58, and 0.78 to 3.77 (?) | N/R | N/R | Against the POMA and SPPB (#):  Vs POMA balance: r_S_ = 0.28 to 0.48; (−)  Vs SPPB balance: r_S_ = 0.21 to 0.45.  (+) |
| **Pooled or summary result (overall rating)** | | ICC = 0.77−0.92  (+) | MIC not defined (?) | N/R | N/R | Inconsistent  (±) |
| One leg standing (OLS) | Giorgetti, 1998 [40] | Inter-rater,  ICC = 0.75  (+) | N/R | N/R | N/R | N/R |
|  | Lin, 2004 [41] | Test-Retest,  ICC ≥ 0.93  (+) | N/R | N/R | Predictive ability (Odds ratios estimated): Falls: 0.99 (0.98–1.01); ADL decline: 0.98 (0.97–0.99); ADL improvement: 1.00 (0.99–1.01)  (?) | 1) Discriminant validity: Subjects who had experienced a fall in the previous year (p=0.001), were older, used a walking aid, and suffered more ADL disabilities stood a shorter time on the OLS (p=0.000). (+)  2) Convergent validity: Not moderately or strongly correlated with any other measures. (−) |
| **Pooled or summary result (overall rating)** | | ICC ≥ 0.75  (+) | N/R | N/R | Indeterminate  (?) | (+), for discriminative validity  (−), for convergent validity |
| Tandem Gait (TG) | Giorgetti, 1998 [40] | Inter-rater,  ICC = 0.31  (−) | N/R | N/R | N/R | N/R |
| **Pooled or summary result (overall rating)** | | ICC = 0.31  (−) | N/R | N/R | N/R | N/R |
| The five-times-sit-to-stand test (FTSST) | Goldberg, 2012 [57] | Test-retest,  ICC = 0.95  (+) | LoA : –2.6 to +2.6  SEM = 0.9  MDC = 2.5  (?) | N/R | N/R | Vs clinical balance measures,  (#): Vs TUG, r=0.64 (+)  Vs FR, r= −0.36 (−) |
| **Pooled or summary result (overall rating)** | | ICC = 0.95  (+) | MIC not defined (?) | N/R | N/R | Inconsistent  (±) |
| The Maximum Step Length (MSL) test | Goldberg, 2010 [58] | Intra-rater, same day, ICC = 0.96  Intra-rater, one month, ICC ≥ 0.90  Same-day Inter-rater, ICC = 0.95  (+) | Intra-rater, same day, SEM ≤ 1.87  Intra-rater, 1 month, SEM ≤ 2.64  Same-day inter-rater, SEM = 1.75  MDC, 1 month interval = 7.32 (rater 1) to 7.29 (rater 2) (?) | N/R | N/R | 1) Against other clinical balance measures (#): Vs Single Limb Stance Time test (r=0.68); Vs  FR test (r=0.65) (+)  2) Relationships between MSL test and number of falls in past 12 months (**): r= − 0.32 (p = 0.06)  (−) |
| **Pooled or summary result (overall rating)** | | ICC = 0.90−0.96  (+) | MIC not defined (?) | N/R | N/R | Inconsistent  (±) |
| The Thirty Rapid-Step test (30-RST) | Goldberg, 2015 [59] | Test–retest,  ICC = 0.85  (+) | LoA = −5.1 to + 13.7  SEM = 3.4  MDC = 9.4  (?) | N/R | N/R | Against other balance tests  (#): Vs dynamic performance-based measures (r_S_ = − 0.35 to 0.73) (+)  Vs performance-based measures of static balance (r_S_ = 0.04 to 0.19) (−) |
| **Pooled or summary result (overall rating)** | | ICC = 0.85  (+) | MIC not defined (?) | N/R | N/R | Inconsistent  (±) |
| The Community Balance and Mobility Scale (CBM) | Weber, 2018 [60] | Inter-rater,  ICC = 0.97  Intra-rater,  ICC = 1.00  (+) | N/R | Cronbach’s α =0.88.  Item-total correlations ranged from 0.81 to 0.28 | N/R | 1) Against other balance and mobility tests (#): With FAB  (ρ=0.75); vs 3MTW errors (ρ=−0.61): (+);  Vs gait speed (ρ = 0.46) (+); with TUG (ρ = 0.42); with 8-level balance scale (ρ = 0.35) (−), and with 3MTW time (ρ = −0.35); (−).  2) Discriminative ability (fallers vs non-fallers): No statistically significant differences (p = 0.09) (−) |
| **Pooled or summary result (overall rating)** | | ICC ≥ 0.97  (+) | N/R | Criteria not met  (?) | N/R | Inconsistent  (±) |
| The ***German***-Community Balance and Mobility Scale (German CBM) | Gordt, 2019 [61] | Inter-rater,  ICC = 0.996  Intra-rater,  ICC = 0.998  (+) | N/R | Cronbach’s α =0.998 | Against the BBS (§),  r_S_ = 0.78 | Against other balance tools (#): with the Fullerton Advanced Balance Scale (r_S_ = 0.85) (+);  with the “30 s Chair Stand Test” (r_S_ = 0.43) (+); with “3 m Tandem Walk” (r_S_= –0.61) (+); Vs Timed Up and Go –(r_S_=0.58). (+) |
| **Pooled or summary result (overall rating)** | | ICC ≥ 0.99  (+) | N/R | Criteria not met  (?) | r > 0.70  (+) | Hypothesis confirmed  (+) |
| The Shortened version of the Community Balance and Mobility Scale (s-CBM) | Gordt, 2020 [62] | N/R | N/R | Cronbach α =0.84 for the entire s-CBM (factor 1: 0.81; factor 2: 0.71) | N/R | 1) Against Established Assessments for Balance (#): Vs CBM (r = 0.97); vs “Fullerton Advanced Balance Scale” (r= 0.72); vs Timed Up-and-Go = (r= −0.44). (+)  2) Discriminant validity (fallers/non-fallers): Falls in the previous 6 months, AUC s-CBM = 0.66 (0.44–0.87), indicating a limited discriminatory ability (−) |
| **Pooled or summary result (overall rating)** | | N/R | N/R | Criteria met  (+) | N/R | Inconsistent  (±) |
| The “Step-Ex”  (New Development Technologies [NDT], Stockholm, Sweden) | Halvarsson, 2012 [63] | Test–retest, healthy elderly: ICC = 0.83 to 0.87.  elderly with balance problems: ICC = 0.71 to 0.83  (+) | Healthy elderly: SEM = 0.02–0.06  Elderly with balance problems: SEM = 0.03–0.08  SRD, both groups = 0.07 to 0.22  All values within or very close to LoA. | N/R | N/R | N/R |
| **Pooled or summary result (overall rating)** | | ICC = 0.71−0.87  (+) | MIC not defined (?) | N/R | N/R | N/R |
| Tinetti's POMA balance subscale | Harada, 1995 [30] | N/R | N/R | N/R | Sensitivity: 68%  Specficity: 78%  (Both at cut-off score of 14, by ROC analysis) | N/R |
|  | Lin, 2004 [41] | Test-Retest,  ICC ≥ 0.93 | N/R | N/R | Predictive ability (Odds ratios estimated): Falls: 0.96 (0.93–0.99); ADL decline: 0.92 (0.90–0.94); ADL improvement: 0.94 (0.91–0.97)  (?) | 1) Discriminant validity: Subjects who were older, had experienced a fall in the previous year, used a walking aid, and suffered more ADL disabilities obtained lower scores on the Tinetti Balance (p=0.000). (+)  2) Convergent validity, Vs Functional reach (FR), r = 0.48 (−) |
| **Pooled or summary result (overall rating)** | | ICC ≥ 0.93  (+) | N/R | N/R | Indeterminate  (?) | (+), for discriminative validity  (−), for convergent validity |
| The Short Berg Balance Scale (BBS-9) | Hohtari-Kivimaki, 2012 [64] | N/R | N/R | Cronbach’s α=0.69 | Vs static and dynamic aspects of balance measured with a force platform (§),  r_S_= −0.247 to −0.454 (−) | N/R |
| **Pooled or summary result (overall rating)** | | N/R | N/R | Criteria met  (+) | r < 0.70  (−) | N/R |
| The Multi-Directional Reach Test (MDRT) | Holbein-Jenny, 2005 [31] | Test-retest,  ICC = 0.66 to 0.83  Inter-rater,  ICC = 0.91 to 0.98  (+) | N/R | BBS and 4 separate MDRT directions, Cronbach’s α=0.80  With only the 4 MDRT directions, Cronbach’s α=0.89 (?) | Against the BBS (§),  r = 0.53 to 0.80. Stronger correlations with the forward and backward MDRT directions (0.78 and 0.77) (+) than the lateral directions (0.53 and 0.63) | Vs ABC (#), r = 0.41 to 0.59 (+) |
|  | Newton, 2001  [65] | Test-retest,  ICC = 0.0926 to 0.942  (+) | N/R | Cronbach’s α=0.842 (?) | Against the BBS (§),  r = 0.356 to 0.476 (−) | 1) Against scores on the TUG (#), r = −0.442 to −0.260 (−)  2) To examine the mean for each direction of the MDRT in relation to health status, use of an assistive device, and history of falls (**): No significance associations (p>0.5) noted. (−) |
| **Pooled or summary result (overall rating)** | | ICC = 0.83−0.98  (+) | N/R | Criteria not met  (?) | Inconsistent  (±) | (±), for convergent validity  (−), for discriminative validity |
| The Kinect system (Kinect for Xbox 360™, Microsoft Corp, Seattle, WA, USA) | Hsiao, 2018 [66] | Within-session, ICC = 0.820 and 0.775  (+) | N/R | N/R | Vs posturography (§), r=−0.257 and −0.062 (−) | Against traditional functional reach (#),  Maximum forward reach, r = 0.719 (+)  Maximal velocity at the halfway time, r = 0.239 |
| **Pooled or summary result (overall rating)** | | ICC ≥ 0.775  (+) | N/R | N/R | r < 0.70  (−) | r_S_ > 0.50  (+) |
| The ***Turkish version*** of Fullerton Advanced Balance (FAB-T) scale | Iyigun, 2018 [67] | Inter-rater,  ICC = 0.96  Intra-rater,  ICC = 0.96  (+) | N/R | N/R | Against the BBS (§),  r_S_ = 0.70 | N/R |
| **Pooled or summary result (overall rating)** | | ICC = 0.96  (+) | N/R | N/R | r_S_ = 0.70  (+) | N/R |
| The Fullerton Advanced  Balance (FAB) Scale | Klein, 2011  [68] | N/R | N/R | N/R | N/R | N/R |
|  | Rose, 2006 [69] | Test-Retest,  ρ = 0.96  Intra-rater,  ρ = 0.97 to 1.00.  Inter-rater,  ρ = 0.94 to 0.97  (?) | N/R | N/R | Against the BBS (§),  r_S_ = 0.75 | N/R |
| **Pooled or summary result (overall rating)** | | ICC not reported  (?) | N/R | N/R | r ≥ 0.70  (+) | N/R |
| The parallel walk test | Lark, 2009 [70] | N/R | N/R | N/R | N/R | 1) Against the tandem stance static balance test (#), r_S_ = 0.28 to 0.49 (−)  2) Discrimination (percentage correctly classified as fallers): Significant difference (p<0.05) for 2 of the 3 widths (20 and 30.5cm) but not for 38cm. (?) |
| **Pooled or summary result (overall rating)** | | N/R | N/R | N/R | N/R | Hypotheses not confirmed  (−) |
| The timed up and go (TUG) test | Galhardas, 2020 [39] | Test-retest,  ICC = 0.99  (+) | SEM = 0.5  MDC = 1.5 | N/R | N/R | N/R |
|  | Lin, 2004 [41] | Test-Retest,  ICC ≥ 0.93  (+) | N/R | N/R | Predictive ability (Odds ratios estimated): Falls: 1.02 (1.01–1.03); ADL decline: 1.03 (1.01–1.04); ADL improvement: 1.03 (1.01–1.05)  (?) | 1) Discriminant validity: Subjects who were older, had experienced a fall in the previous year, used a walking aid, and suffered more ADL disabilities required a longer time to complete the TUG (p=0.000). (+)  2) Convergent validity, Vs Tinetti Balance scores, r = −0.55 (+) |
|  | Nightingale, 2019 [71] | N/R | N/R | N/R | N/R | Against OptoGait mean and standard deviation variable scores  (#), r= −0.067 to −0.68  (+) |
|  | Pelicioni, 2022 [33] | Inter-rater,  via telehealth,  ICC = 1.00  Intra-rater,  in person,  ICC = 0.83  (+) | N/R | N/R | N/R | Against Physiological Profile Assessment (PPA) fall risk score (#), r= 0.54 and 0.64 (+) |
|  | Yingyongyudha, 2016 [24] | N/R | N/R | N/R | N/R | Comparison of balance scores  between the participants (with/without history of falls)  (**): p<0.05 (+) |
| **Pooled or summary result (overall rating)** | | ICC ≥ 0.83  (+) | MIC not defined  (?) | N/R | r not reported  (?) | Hypotheses confirmed  (+) |
| The Balance Computerized Adaptive Testing (Balance CAT) | Lu, 2015 [72] | N/R | IRT reliability (relative to the SEM),  Mean (SD) IRT reliability = 0.93 (0.03)  IRT reliability of each score = 0.85 to 0.97 | N/R | Against the BBS,  r = 0.90 | Discriminative validity: The mean scores of the Balance CAT between the four groups with various levels of dependence were significantly different (p < 0.001). (+) |
| **Pooled or summary result (overall rating)** | | N/R | MIC not defined  (?) | N/R | r = 0.90  (+) | Hypothesis confirmed  (+) |
| The MyBalance test | Mansson, 2021 [73] | N/R | N/R | N/R | N/R | Against Mini-BESTest; FR; Modified 4-stage Balance Test; Modified Maximal Stepping test (#): Moderate to poor and low correlations between the clinical balance instruments and the sensor-test variables *for Feet Together*. No significant correlations observed between the *Semi Tandem Stance* and the clinical instruments. (?) |
| **Pooled or summary result (overall rating)** | | N/R | N/R | N/R | N/R | r not reported  (?) |
| The Brief‐Balance Evaluation Systems Test (Brief‐BESTest) | Marques, 2016 [20] | Inter-rater,  ICC = 0.93  Test-Retest,  ICC = 0.82  (+) | Inter-rater,  LoA = −2.5 to 2.1  Test-Retest,  MDC = 4.1  SEM = 1.5  LoA = −4.4 to 3.5 (?) | N/R | Against the BBS,  r_S_ ≥ 0.83  (+) | 1) Ability to identify fall status (with/without history of falls)  (**): P<0.007; AUC = 0.76 95% CI: 0.66 - 0.86. (+)  2) Against scores of the ABC, Mini-Best, and Best (#):  ABC, r_S_ =0.61 (+)  Mini-Best, and Best, r_S_ ≥ 0.83 (+) |
|  | O'Hoski, 2015 [21] | N/R | N/R | N/R | N/R | 1) Convergent validity:  Vs ABC: r = 0.66 (+); Vs PASE: r = 0.40 (+); Vs TUG: r = −0.60 (+); Vs SLS: r = 0.77 (+) (p<0.001)  2) Discriminative ability (fallers vs non-fallers: p = 0.08. (−) |
|  | Viveiro, 2019 [22] | Inter-rater,  ICC = 0.993  Test-retest,  ICC = 0.939  (+) | SEM = 1.4  MDC = 4.0  (?) | N/R | 1) Sensitivity = 0.944  Specificity = 0.581  2) Against the BBS (§): Good agreement, PABAK ≥ 83.7%. (+) | 1) Ability to identify fall status (fallers vs non-fallers)  (**): P=0.001 (+)  2) Agreement between the balance tests (#): Good agreement. PABAK = 83.7% to 98.0%. (+) |
| **Pooled or summary result (overall rating)** | | ICC = 0.82−0.99  (+) | MIC not defined  (?) | N/R | r_S_ ≥ 0.83  (+) | (±), for discriminative validity  (+), for convergent validity |
| The Functional Gait Assessment-***Brazil***  (FGA- Brazil) | Marques, 2021 [74] | N/R | N/R | N/R | Against the BBS,  r_S_ = 0.80 (+) | Ability to differentiate the groups with low and high concern about falls (**): Significant difference found (p<0.001) in scores between the groups. (+) |
|  | Kirkwood, 2021 [75] | Intra and inter-rater, ICC > 0.90 | SEM = 1.03 to 1.52 | Cronbach’s α for the total items combined =0.858. Total correlations (r) between the items and the total score of the FGA-Brazil >0.30  (?) | N/R | N/R |
| **Pooled or summary result (overall rating)** | | ICC > 0.90  (+) | MIC not defined  (?) | Criteria not met  (?) | r_S_ = 0.80  (+) | Hypothesis confirmed  (+) |
| The ‘‘Get-up and Go’’ Test | Mathias, 1986 [76] | Observer variation, Kendall coefficient of concordance = physiotherapists, W=0.85, p<0.001; senior doctors, W = 0.686, p<0.001. (?) | N/R | N/R | Against sway as recorded from the Kistler force platform (§): r = 0.50 (−) | N/R |
| **Pooled or summary result (overall rating)** | | ICC not reported  (?) | N/R | N/R | r < 0.70  (−) | N/R |
| The apparatus for assessment of postural responses | Matjacic, 2010  [77] | N/R | N/R | N/R | Against the BBS (§),  r = 0.667 (−) | Comparison of means of peak amplitude responses between Fallers and non-Fallers (**): Statistically significant differences (p=0.025 and p=0.009). (+) |
| **Pooled or summary result (overall rating)** | | N/R | N/R | N/R | r < 0.70  (−) | Hypothesis confirmed  (+) |
| A comprehensive set of inertial sensor measures of postural sway  (The Balance Score (BS) & The Weighted Balance Score (WBS)) | Mcmanus, 2022 [78] | Test-retest, ICC<0.75 and ICC≥0.75 (+) | N/R | N/R | N/R | 1) Against the TUG time (#),  ρ = 0.30 to 0.36 (−)  2) Comparison of values of the BS and WBS for participants in two categories (‘normal’ vs ‘impaired’ balance) (**): Statistically significant difference (p<0.01). (+) |
| **Pooled or summary result (overall rating)** | | ICC ≥ 0.75  (+) | N/R | N/R | N/R | Inconsistent  (±) |
| The Modified Version of the Community Balance and Mobility Scale (CBMS-Home) | Ng, 2021 [79] | Test-retest, ICC for CBMS-Home= 0.95  (+) | SEM = 3.12  MDC = 8.7 (for CBMS-Home)  (?) | Cronbach α values for the individual  components (ie, balance, coordination, and muscle performance) =  0.88, 0.90, and 0.90, respectively.  Overall Cronbach  α = 0.94  (?) | N/R | Against the Functional Reach Test (FRT), and Step Test (ST)  (#): with FRT (ρ = 0.39) (−)  with ST (ρ = 0.63) (+)  CBMS vs CBMS-Home (#),  ICC = 0.94 (+) |
| **Pooled or summary result (overall rating)** | | ICC = 0.95  (+) | MIC not defined  (?) | Criteria not met  (?) | N/R | Inconsistent  (±) |
| The Pavia Instrumented Tinetti Test (PITT) | Panella, 2008 [80] | N/R | N/R | Cronbach’s  α =0.88  (?) | Sensitivity = 85.5%  Specificity = 75%  (Optimal threshold = 153.7) (?) | 1) Against the global score of the Tinetti test (#), r= 0.39 and 0.41 (+)  2) Discriminant validity: PITT discriminated between the two considered samples (the fact of being healthy or not) on the basis of their performance, with sensitivity and specificity greater than 80%. (+) |
| **Pooled or summary result (overall rating)** | | N/R | N/R | Criteria not met  (?) | r not reported  (?) | Hypotheses confirmed  (+) |
| The Dynamic Gait Index (DGI) | Pelicioni, 2022 [33] | Inter-rater,  via telehealth,  ICC = 0.85  Intra-rater,  in person,  ICC= 0.86  (+) | N/R | N/R | N/R | Against Physiological Profile Assessment (PPA) fall risk score (#), r= −0.57 and −0.53 (+) |
| **Pooled or summary result (overall rating)** | | ICC ≥ 0.85  (+) | N/R | N/R | N/R | r > 0.50  (+) |
| The ***Danish Version*** of the Dynamic Gait Index (Danish DGI) | Jønsson, 2011 [98] | Intra-rater,  ICC = 0.89  Inter-rater,  ICC = 0.82  (+) | Intra-rater agreement,  SRD = 3.49  Inter-rater agreement, SRD=3.99 | N/R | N/R | N/R |
| **Pooled or summary result (overall rating)** | | ICC = 0.82−0.89  (+) | MIC not defined (?) | N/R | N/R | N/R |
| The Functional Gait  Assessment (FGA) | Pelicioni, 2022 [33] | Inter-rater, via telehealth,  ICC = 0.80  Intra-rater, in person, ICC = 0.87 | N/R | N/R | N/R | Against Physiological Profile Assessment (PPA) fall risk score (#), r= − 0.64 and − 0.68 (+) |
|  | Wrisley, 2010 [99] | N/R | N/R | N/R | 1) Concurrent, vs BBS: r=0.84 (+)  2) Predictive validity: An FGA score of ≤22/30 provides predictive validity. | 1) Against TUG and ABC (#),  Vs ABC, r=0.53 (+)  Vs TUG, r=−0.84 (+)  2) Discriminative validity (increased risk for falls by the TUG and DGI): An FGA score of ≤22/30 provides discriminative validity (+) |
|  | Beninato, 2016 [100] $ | N/R | N/R | N/R | N/R | N/R |
| **Pooled or summary result (overall rating)** | | ICC ≥ 0.80  (+) | N/R | N/R | r=0.84  (+) | Hypotheses confirmed  (+) |
| The NIH Toolbox^®^ Standing Balance Test | Peller, 2022 [81] | Test–retest,  ICC = 0.84  (+) | MDC = 0.65 | N/R | Criterion validity, Against the Biodex Balance System SD,  r = 0.52 (−) | N/R |
| **Pooled or summary result (overall rating)** | | ICC = 0.84  (+) | MIC not defined  (?) | N/R | r < 0.70  (−) | N/R |
| The Biodex SD (Biodex Medical Systems, Shirley NY) | Riemann, 2017 [82] | Test–retest,  ICC = 0.74 to 0.86 | SEM = 15.9 to 23.6% | N/R | N/R | N/R |
| **Pooled or summary result (overall rating)** | | ICC = 0.74−0.86  (+) | MIC not defined  (?) | N/R | N/R | N/R |
| The Balance Scale (by Roberts) | Roberts, 1987 [83] | N/R | N/R | Standardized item coefficient alpha, for total scale, and for each of the 4 factors = 0.60 to 0.82 | N/R | N/R |
| **Pooled or summary result (overall rating)** | | N/R | N/R | Cronbach’s α not reported (?) | N/R | N/R |
| The ***Turkish Version*** of the Berg Balance Scale (BBS) | Sahin, 2008 [84] | Test-retest,  ICC = 0.98  Intra- and inter- rater, ICC = 0.98 and 0.97 (+) | N/R | Cronbach’s α =0.93 | N/R | Against the Modified Barthel Index (MBI) scores, r = 0.67 (+)  Against the TUG, r = −0.75 (+) |
| **Pooled or summary result (overall rating)** | | ICC = 0.97−0.98  (+) | N/R | Criteria not met  (?) | N/R | r > 0.50  (+) |
| The ***Persian version*** of the Berg Balance Scale (BBS) | Salavati, 2012 [85] | Inter-rater,  ICC = 0.93  Intra-rater,  ICC = 0.95 (+) | N/R | Cronbach’s α=0.62  (?) | N/R | Against the TUG (#), r_S_=−0.74 |
| **Pooled or summary result (overall rating)** | | ICC = 0.93−0.95  (+) | N/R | Criteria not met  (?) | N/R | r > 0.50  (+) |
| The Nintendo Wii Fit *exergame* | Sato, 2021 [86] | N/R | N/R | N/R | N/R | Against CTSIB parameters (#),  r= −0.52 to −0.48. (+) |
| **Pooled or summary result (overall rating)** | | N/R | N/R | N/R | N/R | r > 0.50  (+) |
| The Wii Stillness (WST) Test | Simms, 2020 [88] | N/R | N/R | N/R | Concurrent validity, against balance metrics collected using the force platform,  ρ= −0.61 to −0.33  (−) | N/R |
| **Pooled or summary result (overall rating)** | | N/R | N/R | N/R | r < 0.70  (−) | N/R |
| The short form of the Fullerton Advanced Balance (SF-FAB) scale | Sinaei, 2021 [89] | Intra-rater,  ICC = 0.94–0.99  Inter-rater,  ICC = 0.92–0.98  (+) | SEM = 0.54  MDC = 1.49 | Cronbach’s α=0.77; Considering item deletion, Cronbach’s α ranged from 0.63 to 0.78. (?) | Sensitivity = 0.82  Specificity = 0.76 | Fall Status (Participants with two or more self-reported fall events during the year prior to the study were categorized as fallers) (**): OR=0.67 (95% CI [0.55, 0.82]) for the likelihood of falling. (+) |
| **Pooled or summary result (overall rating)** | | ICC = 0.92–0.99  (+) | MIC not defined  (?) | Criteria not met  (?) | r not reported  (?) | Hypothesis confirmed  (+) |
| The 'balance meter' | Stokes, 1998  [90] | Test-retest:  reliability was demonstrated (?) | For antero-posterior data, LoA = 0.89 ± 8.14  For lateral data, LoA = 0.69 ± 11.6 | N/R | N/R | 1) Against the Tinetti POMA (#),  r = −0.222 to −0.152 (−)  2) Ability to differentiate between fallers and non-fallers (**), p=0.003  and p=0.005 (+) |
| **Pooled or summary result (overall rating)** | | ICC not reported  (?) | MIC not defined  (?) | N/R | N/R | Inconsistent  (±) |
| The AMTI Accusway system for balance and postural sway measurement (Advanced Mechanical Technology, Inc.,  Watertown, Massachusetts) | Swanenburg, 2008 [91] | Inter-rater and Test-retest,  ICC = 0.52 to 0.89 | SDD = 0.37 to 3.75  LoA analysis: No systematic differences between test-retest. | N/R | N/R | N/R |
| **Pooled or summary result (overall rating)** | | ICC = 0.52−0.89  (+) | MIC not defined  (?) | N/R | N/R | N/R |
| A dual-task computer game-based platform (TGP) | Szturm, 2015 [92] | Test retest,  ICC = 0.55 to 0.7 | SEM **=** 8% to 12% | N/R | N/R | N/R |
| **Pooled or summary result (overall rating)** | | ICC = 0.55−0.7  (+) | MIC not defined  (?) | N/R | N/R | N/R |
| The Modified Bathroom Scale | Vermeulen, 2012 [93] | N/R | N/R | N/R | N/R | 1) Nursing home patients compared with active community-dwelling elderly people (**), nursing home patients had significantly lower scores on the balance test. (+)  2) Against the POMA, TUG, and FTBS scores (#): In nursing home patients, r= 0.49, −0.60, and 0.63. (+)  In active community-dwelling elderly people, r= −0.04, −0.42, and 0.33. (−) |
| **Pooled or summary result (overall rating)** | | N/R | N/R | N/R | N/R | Inconsistent  (±) |
| The instrumented modified Clinical Test of Sensory Interaction on Balance (i-mCTSIB) utilizing the  Neurocom Very Simple Rehab (VSR) Sport force plate (Natus Medical  Incorporated, Pleasanton, California). | Watson, 2021 [94] | Test-retest,  ICC = 0.898 | SEM = 0.122  MDC_90_ = 0.285 | N/R | N/R | N/R |
| **Pooled or summary result (overall rating)** | | ICC = 0.898  (+) | MIC not defined  (?) | N/R | N/R | N/R |
| Models for estimating decline in balance using accelerometry-based gait features | Simila, 2017 [95] | N/R | N/R | N/R | 1) Estimation of BBS result at baseline with baseline gait features; & Correlation plots for estimated and measured BBS scores (§): Mean normalized RMSE of estimation in ten folds was 0.28 for BBS score. (?)  2) Prediction of decline in balance: Mean accuracy of classification was 69.2% for decline in BBS total score and 78.5% for decline in one leg stance. At the sensitivity level of 80%, the specificities were 73% and 67% for predicting decline in BBS total score and one leg stance respectively. | Estimation of the TUG and 4 m walk test results at baseline with baseline gait features; & Correlation plots for estimated and measured TUG and 4 m walk test scores (#):  Mean normalized RMSE of estimation in ten folds was, 0.18 for TUG time, and 0.22 for 4 m walk time, respectively. (?) |
| **Pooled or summary result (overall rating)** | | N/R | N/R | N/R | r not reported  (?) | r not reported  (?) |
| The FICSIT Balance Scales (FICSIT-3 and FICSIT-4) | Rossiter-Fornoff, 1995 [96] | Test-retest,  r = 0.66  (?) | N/R | N/R | N/R | 1) Against age, gait speed, stride length, the Sickness Impact Profile (SIP) ambulation, mobility, and body care and movement (BCM) subscales at baseline (#): Significant correlations (values not reported). (?)  2) Comparison of nursing homes residents scores to community-dwellings (**): Nursing homes residents were substantially worse at maintaining balance. (+) |
| **Pooled or summary result (overall rating)** | | ICC not reported  (?) | N/R | N/R | N/R | Hypothesis confirmed  (+) |
| The Wii Balance Board™ (WBB) | Olvera-Chavez, 2013 [97] | N/R | N/R | N/R | N/R | Against the SPPB (#), Weighted Kappa = 0.6 (+) |
|  | Scaglioni-Solano, 2014 [87] | Test–retest,  ICC = 0.64− 0.85  (+) | SEM = 7.6–13.9%,  MDC = 21.1–38.5% | N/R | Concurrent validity, against force plate (FP) data: Regression model shows that Wii BB is able to explain most of the variation in CoP displacement data of the FP (between 78 and 96%) for all testing conditions. (?) | N/R |
| **Pooled or summary result (overall rating)** | | ICC = 0.64− 0.85  (+) | MIC not defined  (?) | N/R | r not reported  (?) | Hypothesis confirmed  (+) |
| The Balance Tracking System (BTrackS) | Levy, 2018 [101] | Test-retest,  ICC = 0.83  (+) | SEM= 3.47 and 7.0 cm.  MDC = 9.6 and 19.4 cm. | Internal consistency reliability of 3 trials, ICC = 0.95, and 0.97 (?) | Concurrent, vs Force plate data, r = 0.82 and 0.89 (+) | N/R |
| **Pooled or summary result (overall rating)** | | ICC = 0.83  (+) | MIC not defined  (?) | Criteria not met  (?) | r ≥ 0.82  (+) | N/R |
| The NeuroCom Smart Equitest Research System (Natus Medical Inc, Pleasanton, California) | Harro, 2019 [102] | Test-retest,  ICC= 0.71−0.90 | SEM= 0.1−9.0  MDC = 0.3−25.0 | N/R | N/R | 1) Against gait and balance measures (#)  Vs Mini-BESTest, r = 0.347 (−)  Vs Functional Gait Assessment, r=0.490 (−)  Vs 6MWT: r= 0.309 (+)  2) Age and activity level association with force platform measures performance (**): No significant association (−) |
| **Pooled or summary result (overall rating)** | | ICC ≥ 0.71  (+) | MIC not defined  (?) | N/R | N/R | Hypotheses not confirmed  (−) |

§ = Concurrent validity assumed; ‡ = Predictive validity assumed; ** = Discriminative validity assumed; # = Convergent validity assumed; N/R = Not reported; $ = Only structural validity assessed.

Measurement property rating: sufficient (+), insufficient (−), inconsistent (±), indeterminate (?)

ABC = The Activities-specific Balance Confidence Scale; DSE = direction-sensitive evaluation (new strategy proposed by the authors in this study); 8LBS = The 8-level balance scale; APSI = anteroposterior stability index ; MLSI = mediolateral stability index; PABAK = prevalence-adjusted bias-adjusted kappa; PASE = Physical Activity Scale for the Elderly; FAB = The Fullerton Advanced Balance scale ; 3MTW = The three meter tandem walk; TUG = The Timed-Up-and-Go test; SRD = Smallest Real Difference; Falls Efficacy Scale-International (FES-I); SEM = Standard Error of Measurement; MDC = Minimum Detectable Change; SD = Standard Deviation; POMA = Performance-Oriented Mobility Assessment (Tinetti); CTSIB = Clinical Test of Sensory Interaction on Balance; FRT = The Functional Reach Test; SLS = Single Leg Stance; MDRT = The Multi-Directional Reach Test; STT = The Stepping Threshold Test

**Appendix 4.b**: Measurement properties of Muscle strength assessment tools

| **Instrument** | **Reference** | **Reliability** | | | **Validity** | |
| --- | --- | --- | --- | --- | --- | --- |
|  |  | ***Reliability*** | ***Measurement error*** | ***Internal consistency*** | ***Criterion validity*** | ***Hypothesis testing for construct validity*** |
| The JAMAR hand-held hydraulic dynamometer | Abizanda, 2012  [115] | Test–retest, ICC for 6 different muscle groups, = 0.98−0.99 (+) | N/R | N/R | N/R | Against functionality tests (#): Good to very good linear association with POMA, 6-min walk test, and TUG. (?) |
|  | Silva, 2019 [116] | Inter-rater, ICC for nursing home, day care, and all patients = 0.90−0.97 (+) | SEM, 1.66−1.82  MDC, 4.60−5.04  (?) | N/R | N/R | N/R |
| ***Pooled or summary result (overall rating)*** | | ICC = 0.90−0.99  (+) | MIC not defined  (?) | N/R | N/R | r not reported  (?) |
| A uni-axial load cell device | Alqahtani, 2019 [118] | Test–retest, ICC for 3 different muscle groups = 0.90−0.99 (+) | SEM = 6.78 (Hip abduction) to 28.07 (Plantarflexion)  MCID values reported.  *SDC Estimated from SEM as = 1.96 × √2 × SEM = 18,79 to 77,81* | N/R | N/R | Against mobility measurements (#): Vs 6MWT, gait speed, r_S_ = 0.22− 0.29 (−);  Vs GES, and repeated chair-stands test, r_S_ = 0.21−0.28 (−);  Vs SPPB, r_S_ = 0.28− 0.38 (+)  F8WT, r_S_ =− 0.15 to − 0.26 (−). |
| ***Pooled or summary result (overall rating)*** | | ICC = 0.90−0.99  (+) | SDC > MIC (MCID)  (−) | N/R | N/R | Inconsistent  (±) |
| The calf-raise senior (CRS) test | Andre, 2016 [119] | Inter-rater, Intra-rater, ICC = 0.79 – 0.93 (+)  Test–retest, ICC = 0.90 (+) | SEM = 1.8 | N/R | Concurrent validity, against measures from the Biodex,  r = 0.77−0.87 (+) | Comparison of test results between subgroups of subjects (**): Participants with better results in the CRS test were younger and presented higher levels of physical activity and functional fitness (statistically significant differences). (+) |
| ***Pooled or summary result (overall rating)*** | | ICC = 0.79−0.93  (+) | MIC not defined  (?) | N/R | r ≥ 0.70  (+) | Hypothesis confirmed  (+) |
| The Handheld Dynamometry (HHD): The Lafayette Manual Muscle Tester, Model # 01163, (Lafayette Instrument Inc., Lafayette, Indiana) | Arnold, 2010 [120] | Intra-rater, ICC = 0.90 to 0.98 (+)  Test-retest,  ICC 0.76 to 0.93 (+)  Inter-rater, ICC = 0.48 to 0.94 (+) | Test-retest and inter-rater,  SEM = 0.87 to 1.89 | N/R | Concurrent validity, against the Biodex 3,  r = 0.57 to 0.86 (+) | N/R |
|  | Bohannon, 2005 [121] | Test–retest, ICC = 0.95 and 0.91(left and right) (+) | Technical errors of measurement, 15.8 and 21.3 N | N/R | N/R | N/R |
|  | Bohannon, 1997 [122] | N/R | N/R | Between strengths of different muscle actions  Cronbach α = 0.59 to 0.88 | N/R | N/R |
|  | Martin, 2006 [123] | N/R | N/R | N/R | Against the Biodex (§), r = 0.91 (+) | N/R |
| ***Pooled or summary result (overall rating)*** | | ICC = 0.76−0.98  (+) | MIC not defined  (?) | Criteria not met  (?) | r ≥ 0.70  (+) | N/R |
| The Nintendo Wii Balance Board (WBB) | Blomkvist, 2016 [124] | Test–retest,  ICC = 0.96 (+) | SEM = 0.4  LoA= 3.4 | N/R | N/R | Against the Jamar hand dynamometer (#), r = 0.87 (+) |
|  | Jorgensen, 2015 [125] | Reproducibility,  ICC = 0.97 (+) | SEM = 15.6  LoA = 32.4 | N/R | N/R | Against a Stationary isometric dynamometer (#),  ICC = 0.81 to 0.84 (+) |
| ***Pooled or summary result (overall rating)*** | | ICC = 0.96−0.97  (+) | MIC not defined  (?) | N/R | N/R | ICC > 0.50  (+) |
| The Modified Sphygmomanometer Test (MST) | Brito, 2022  [126] | Inter-rater and test-retest for all muscle groups, ICC = 0.80 to 0.99 (+) | SEM = 3.66 to 20.46  SRD = 10.15 to 56.70 | N/R | N/R | Against the Microfet2® and the Saehan® hydraulic handgrip dynamometer (#)  0.80 ≤ r ≤ 0.91, for all muscle groups (dominant side) (+) |
| ***Pooled or summary result (overall rating)*** | | ICC = 0.80−0.99  (+) | MIC not defined  (?) | N/R | N/R | r > 0.50  (+) |
| MicroFET2 hand-held dynamometer (Hoggan Indiustries, Inc., West Jordan, UT, USA) | Buckinx, 2017 [117] | Inter-observer, ICC for 8 different muscle groups,  0.62 to 0.87 (+) | SEM%= 8.80 to 24.42  MDC% = 24.38 to 67.69 | N/R | N/R | N/R |
| ***Pooled or summary result (overall rating)*** | | ICC = 0.62 to 0.87  (+) | MIC not defined  (?) | N/R | N/R | N/R |
| The isometric knee extension (IKE) test (IKE test + strain gauge) | Buendía-Romero, 2021 [127] | Intra- and inter-session, ICC = 0.99 and 0.96 (+) | SEM = 0.09 and 0.26 | N/R | N/R | N/R |
| ***Pooled or summary result (overall rating)*** | | ICC = 0.96−0.99  (+) | MIC not defined  (?) | N/R | N/R | N/R |
| The Q Force | Douma, 2016 [128] | Test-retest, ICC = 0.80 to 0.96 (+) | LoA: 25.1 to 39.9 | N/R | N/R | N/R |
| ***Pooled or summary result (overall rating)*** | | ICC = 0.80−0.96  (+) | MIC not defined  (?) | N/R | N/R | N/R |
| An analog dynamometer (SENSIX®, Poitiers, France) coupled with the DELSYS System (Trigno sensor, DELSYS, INC Boston; MA) | Gafner, 2017 [129] | Test-retest, ICC for 2 different muscle groups, 0.90 to 0.94 (+) | SEM= 0.12 to 1.05  SDD= 32% to 51% | N/R | N/R | N/R |
| ***Pooled or summary result (overall rating)*** | | ICC = 0.90−0.94  (+) | MIC not defined  (?) | N/R | N/R | N/R |
| The Biodex System 3 isokinetic dynamometer (Biodex Medical Systems, Shirley, N.Y., USA) | Hartmann, 2009 [130] | Intra-rater and Inter-rater, different muscle groups,  ICC = 0.81 to 0.99 (+) | SEMs = 6.3 to 17.2%  RLOA % = 18.1 to 47.7 | N/R | N/R | N/R |
|  | Symons, 2004 [131] | Test–retest, ICCs > 0.88 (+) | LoA values larger than desired. | N/R | N/R | N/R |
| ***Pooled or summary result (overall rating)*** | | ICC ≥ 70  (+) | MIC not defined  (?) | N/R | N/R | N/R |
| The Isokinetic dynamometer (KinCom 500H, Chattecx Corp., Hixson, TN, USA) | Holsgaard Larsen, 2007  [108] | Test–retest, 3 muscle groups,  r = 0.64 to 0.91 (?) | N/R | N/R | N/R | N/R |
| ***Pooled or summary result (overall rating)*** | | ICC not reported  (?) | N/R | N/R | N/R | N/R |
| The Leg Press Sled (LPS) | Hutchison, 2006 [132] | Reliability**,**  ICC = 0.98 and 0.93 for the knee angles of 60° and 90°, respectively. (+) | SEM **=** 19.8 kg and 23.1 kg. | N/R | Concurrent validity, against the Biodex System 3,  r = 0.81 and 0.74 (+) | N/R |
| ***Pooled or summary result (overall rating)*** | | ICC ≥ 0.70  (+) | MIC not defined  (?) | N/R | r ≥ 0.70  (+) | N/R |
| The Microfet 2000 strain gauge portable dynamometer (PD) | Karner, 1998 [133] | Intra-rater and inter-rater, ICC = 0.70 and 0.86 (+) | N/R | N/R | N/R | N/R |
| ***Pooled or summary result (overall rating)*** | | ICC ≥ 0.70  (+) | N/R | N/R | N/R | N/R |
| A load cell setup | Keshavarzi, 2022 [134] | Test-retest, ICC = 0.99 (+) | MDC = 4.2  SEM = 1.51 | N/R | N/R | N/R |
| ***Pooled or summary result (overall rating)*** | | ICC = 0.99  (+) | MIC not defined  (?) | N/R | N/R | N/R |
| The push-off test (POT) | Legg, 2020 [135] | Test–retest, ICC = 0.92 (+) | SEM = 1.153  MDC = 2.7 | N/R | N/R | Against the Hand-held dynamometry (#), r = 0.87 |
| ***Pooled or summary result (overall rating)*** | | ICC = 0.92  (+) | MIC not defined  (?) | N/R | N/R | r > 0.50  (+) |
| The functional multi-joint isokinetic dynamometer | Legg, 2020 [135] | Test–retest, concentric and eccentric strengths, ICCs = 0.98 (+) | SEM **=** 0.64 and 0.67  MDC = 1.5 and 1.6 | N/R | N/R | Against Strength by hand-held dynamometry (#), r = 0.93 and 0.90 (+) |
| ***Pooled or summary result (overall rating)*** | | ICC = 0.98  (+) | MIC not defined  (?) | N/R | N/R | r > 0.50  (+) |
| The MyBalance test | Mansson, 2021 [73] | N/R | N/R | N/R | N/R | Against clinical leg strength instruments (#): Low to moderate significant correlations with the Five Times Sit-To-Stand test and the 30s Chair Stand Test; and with the Maximal stepping test. (?) |
| ***Pooled or summary result (overall rating)*** | | N/R | N/R | N/R | N/R | r not reported  (?) |
| The maximal isometric strength test of the trunk (measured by a precalibrated digital loading cell connected to the MuscleLab software) | Mesquita, 2019 [136] | Test-retest, 2 muscle groups, ICC = 0.93 and 0.86 (+) | SEM = 18.4 and 17.6  MDD = 51.1 and 48.9  LoA: Almost all individuals present within acceptable LoA. | N/R | N/R | N/R |
| ***Pooled or summary result (overall rating)*** | | ICC ≥ 0.70  (+) | MIC not defined  (?) | N/R | N/R | N/R |
| The one-repetition maximum (1 RM) using elastic resistance bands test | Nyberg, 2014 [137] | N/R | N/R | N/R | Against isokinetic dynamometer Kin-Com^®^ (§)  ICC = 0.43 to 0.85 (+) | N/R |
| ***Pooled or summary result (overall rating)*** | | N/R | N/R | N/R | Correlation > 0.70  (+) | N/R |
| The lateral step (LS) test | Porto, 2020 [138] | Test–retest,  ICC = 0.95 (+) | N/R | N/R | Against the Biodex System 4 Pro (§)  r = 0.36 (−) | Discrimination of older women with reduced hip abductor peak torque: The LS test is able to discriminate, with low accuracy (AUC = 0.67; p=0.004) (+) |
| ***Pooled or summary result (overall rating)*** | | ICC = 0.95  (+) | N/R | N/R | r < 0.70  (−) | Hypothesis confirmed  (+) |
| Tandem Gait (TG) | Porto, 2020 [138] | Test–retest,  ICC = 0.87 and 0.77 (+) | N/R | N/R | Against the Biodex System 4 Pro (§)  r = −0.20 (−) | Discrimination of older women with reduced hip abductor peak torque: The TG time is able to discriminate, with low accuracy (AUC = 0.65; p=0.009) (+) |
| ***Pooled or summary result (overall rating)*** | | ICC ≥ 0.70  (+) | N/R | N/R | r < 0.70  (−) | Hypothesis confirmed  (+) |
| Single-leg stance (SS) test | Porto, 2020 [138] | N/R | N/R | N/R | Against the Biodex System 4 Pro (§)  r = 0.26 (−) | Discrimination of older women with reduced hip abductor peak torque: AUC = 0.60; p=0.07 (−) |
| ***Pooled or summary result (overall rating)*** | | N/R | N/R | N/R | r < 0.70  (−) | Hypothesis not confirmed  (−) |
| The one repetition maximum (1 RM) using a muscle strength training device for the arm/shoulder (Pull Down, Norway) | Rydwik, 2007 [139] | Test-retest, r=0.97 (?) | LoA = −2.6/+5.6 kg | N/R | N/R | Comparison of subjects with and without previous muscle strength training experience (**): No significant differences between the groups. (−) |
| ***Pooled or summary result (overall rating)*** | | ICC not reported  (?) | MIC not defined  (?) | N/R | N/R | Hypothesis not confirmed  (−) |
| The five-repetition sit-to-stand (STS) test | Schaubert, 2005 [140] | Test-retest,  ICC = 0.82 (+) | Technical error of measurement between sessions, 1.6 to 2.8 | N/R | N/R | N/R |
| ***Pooled or summary result (overall rating)*** | | ICC = 0.82  (+) | MIC not defined  (?) | N/R | N/R | N/R |
| A standardized heel-rise test (Using trunk accelerometry) | Schmid, 2011 [112] | Intra-session  ICC =0.31 and 0.79 (+) | SEM = 0.03 and 0.04  MDD = 0.08 and 0.11 | N/R | N/R | VGRF-derived against VAcc-derived values (#), r = 0.98 (+) |
| ***Pooled or summary result (overall rating)*** | | ICC = 0.31 and 0.79  (+) | MIC not defined  (?) | N/R | N/R | r > 0.50  (+) |
| The one-repetition maximum (1 RM) performed on the Keiser A-300 pneumatic equipment (Keiser Corp., Fresno, CA) or on selectorized weight-stack resistance exercise machines (Cybex VR2; Cybex International Inc., Medway, MA) | Schroeder, 2007 [113] | Test–retest,  r = 0.84 to 0.98 (?) | N/R | N/R | N/R | N/R |
| ***Pooled or summary result (overall rating)*** | | ICC not reported  (?) | N/R | N/R | N/R | N/R |
| Grip strength, measured using a Smedley-type dynamometer (T.K.K.5401, TAKEI Scientifc Instruments Co., Ltd., Niigata, Japan) | Suzuki, 2019 [141] | Relative reliability,  ICC = 0.96 (+) | MDC = 1.18 | N/R | N/R | N/R |
| ***Pooled or summary result (overall rating)*** | | ICC = 0.96  (+) | MIC not defined  (?) | N/R | N/R | N/R |
| Knee extension strength, measured using a handheld dynamometer (μ-Tas F-1; Anima Inc., Tokyo, Japan) | Suzuki, 2019 [141] | Relative reliability, ICC = 0.90 (+) | MDC = 3.29 | N/R | N/R | N/R |
| ***Pooled or summary result (overall rating)*** | | ICC = 0.90  (+) | MIC not defined  (?) | N/R | N/R | N/R |
| The 30-s Chair-Stand Test | Jones, 1999 [142] | Test-retest, ICC = 0.84 for men and 0.92 for women.  (+) | N/R | N/R | N/R | 1) Against the leg press (#), r= 0.77 (+)  2) Discriminant validity: Discrimination of age groups (p<0.01) and low-active vs high-active participants (p<0.0001). (+) |
| ***Pooled or summary result (overall rating)*** | | ICC ≥ 0.70  (+) | N/R | N/R | N/R | Hypotheses confirmed  (+) |

§ = Concurrent validity assumed; ‡ = Predictive validity assumed; ** = Discriminative validity assumed; # = Convergent validity assumed; N/R = Not reported;

Measurement property rating: sufficient (+), insufficient (−), inconsistent (±), indeterminate (?)

MDD = minimum detectable difference; RLOA = ratio of limits of agreement; SDD = smallest detectable difference; SEM = Standard Error of Measurement; SRD = smallest real difference; SPPB = Short Physical Performance Battery balance; GES = Gait Efficacy Scale; F8WT = Figure of 8 Walk Test; 6MWT = Six-Minute Walk Test; MCID = Minimal Clinically Important Difference

**Appendix 4.c**: Measurement properties of Muscle power assessment tools

| **Instrument** | **Reference** | **Reliability** | | | **Validity** | |
| --- | --- | --- | --- | --- | --- | --- |
|  |  | ***Reliability*** | ***Measurement error*** | ***Internal consistency*** | ***Criterion validity*** | ***Hypothesis testing for construct validity*** |
| The 30-s sit-to-stand (STS) muscle power test | Alcazar, 2020 [103] | N/R | N/R | N/R | Against leg extension power assessed by the Nottingham power rig  (§), r=0.75 (+) | N/R |
| ***Pooled or summary result (overall rating)*** | | N/R | N/R | N/R | r ≥ 0.70  (+) | N/R |
| The sit-to-stand (STS) muscle power test | Alcazar, 2018 [104] | N/R | N/R | N/R | N/R | Against power produced in the leg press exercise (#), r = 0.72 (+) |
| ***Pooled or summary result (overall rating)*** | |  |  |  |  | r > 0.50  (+) |
| The sit-to-stand power test (STSp), using a portable linear transducer | Balachandran, 2021 [105] | Test-retest,  ICC = 0.96 (+) | SEM = 70.4 W  SDC = 192.8 W  LoA : − 187.9, 201.1 | N/R | N/R | 1) Against the Pneumatic leg press measures (#), r= 0.90 (+)  2) Discriminant validity, Males peak power vs females: p<0.001 (+) |
| ***Pooled or summary result (overall rating)*** | | ICC = 0.96  (+) | MIC not defined  (?) | N/R | N/R | Hypotheses confirmed  (+) |
| The Vertical jump (VJ) measured by a contact mat | Farias, 2013 [106] | Inter-session,  ICC = 0.91 (+)  Intra-session, ICC session 1 = 0.96; ICC session 2 = 0.95. (+) | SEM, jump 1=0.0037  SEM, jump 2=0.0052 | N/R | N/R | N/R |
| ***Pooled or summary result (overall rating)*** | | ICC = 0.91−0.96  (+) | MIC not defined  (?) | N/R | N/R | N/R |
| The Tendo Weightlifting Analyzer (Trencin, Slovak Republic) | Gray, 2014 [107] | Reliability: The Cronbach’s alpha was 0.98 for the 10 repeated trials. (?) | N/R | N/R | N/R | Against power measured using Center Of Mass (#), r = 0.76 (+).  T-test comparing the two methods (p = 0.86) |
| ***Pooled or summary result (overall rating)*** | | ICC not reported  (?) | N/R | N/R | N/R | r > 0.50  (+) |
| Counter-movement jump (CMJ) test performed on a force platform (Kistler Instruments 9281 B, Winterthur, Switzerland, 40 x 60 cm) | Holsgaard Larsen, 2007 [108] | Test–retest,  r = 0.71 to 0.95, for concentric and eccentric phases. (?) | N/R | N/R | N/R | N/R |
| ***Pooled or summary result (overall rating)*** | | ICC not reported  (?) | N/R | N/R | N/R | N/R |
| The chair stand mean power (CSMP) test, using the Fitro Dyne device (Fitronic S. R. O. Co, Slovakia). | Kato, 2015 [109] | Test-Retest, ICC of repeated measures = 0.92 (W/height), = 0.88 (W/body mass), and = 0.89 (W/BMI)). (+) | N/R | N/R | N/R | Discriminatory performance: Good (AUC = 0.899; 95% CI = 0.833-0.964). (+) |
| ***Pooled or summary result (overall rating)*** | | ICC = 0.88−0.92  (+) | N/R | N/R | N/R | Hypothesis confirmed  (+) |
| The sit-to-stand (STS) performance power using a linear encoder (MuscleLab Power model MLPRO, Ergotest Technology, Langesund, Norway) | Lindemann, 2015 [110] | N/R | N/R | N/R | Against power assessed by the Nottingham Power Rig (§), r = 0.646 (−) | N/R |
| ***Pooled or summary result (overall rating)*** | | N/R | N/R | N/R | r < 0.70  (−) | N/R |
| The Jumping Mechanography | Rittweger, 2004 [111] | Repeated  Measurements,  r = 0.99 | Short-Term Error (EST), EST = 3.60% | N/R | N/R | Against the Chair-Rise Test (#)  r = 0.86 (+) |
| ***Pooled or summary result (overall rating)*** | | ICC not reported  (?) | MIC not defined  (?) | N/R | N/R | r > 0.50  (+) |
| A standardized heel-rise test (Using trunk accelerometry) | Schmid, 2011 [112] | Intra-session,  for VAcc-Derived (vertical acceleration), Pmax (watts/body weight); ICC = 0.80**;** Pmean (watts/body weight): ICC = 0.78 (+) | Pmax (watts/body weight): SEM = 0.025; MDD = 0.069  Pmean (watts/body weight): SEM = 0.015; MDD = 0.043. | N/R | N/R | Against VGRF-(vertical ground reaction force) derived values (#)  r = 0.96, and 0.95, for Pmax, and Pmean, respectively. (+) |
| ***Pooled or summary result (overall rating)*** | | ICC = 0.78−0.80  (+) | MIC not defined  (?) | N/R | N/R | r > 0.50  (+) |
| Unilateral leg extension power (W) using the Bassey Power Rig (University of Nottingham, Nottingham, U.K.) | Schroeder, 2007 [113] | Test–retest,  r = 0.74 (?) | N/R | N/R | N/R | N/R |
| ***Pooled or summary result (overall rating)*** | | ICC not reported  (?) | N/R | N/R | N/R | N/R |
| The Ramp Power Test | Signorile, 2007 [114] | Test–retest,  ICC = 0.921 (+) | Cronbach’s alpha between Day 1 and Day 2 for Test 1 =  0.921 (?) | N/R | N/R | Against isokinetic knee extension and ankle plantar flexion power data (#): Bland–Altman Plots, only one or two points fell outside the 95% CI for comparisons with isokinetic plantar flexion; all points fell within the 95% CI for comparisons with isokinetic knee extension. (?) |
| ***Pooled or summary result (overall rating)*** | | ICC = 0.921  (+) | MIC not defined  (?) | N/R | N/R | r not reported  (?) |

§ = Concurrent validity assumed; ‡ = Predictive validity assumed; ** = Discriminative validity assumed; # = Convergent validity assumed; N/R = Not reported

Measurement property rating: sufficient (+), insufficient (−), inconsistent (±), indeterminate (?)

MDD = minimum detectable difference; SEM = Standard Error of Measurement; SDC = Smallest Detectable Change; LoA = Limits of Agreement; SRD = Smallest Real Difference

**Appendix 4.d**: Measurement properties of Endurance assessment tools

| **Instrument** | **Reference** | **Reliability** | | | **Validity** | |
| --- | --- | --- | --- | --- | --- | --- |
|  |  | ***Reliability*** | ***Measurement error*** | ***Internal consistency*** | ***Criterion validity*** | ***Hypothesis testing for construct validity*** |
| The 6-Minute Walk Test | Rikli, 1998 [143] | Test-retest,  Tests 1 & 2, ICC=0.91  Tests 2 & 3, ICC=0.94  (+) | Absolute consistency: ANOVA analysis revealed a significant overall improvement across trials for total subjects: Lack of stability of group means over the 3 trials (p<0.0001, for Total subjects) (?) | N/R | N/R | 1) Convergent validity, against the Treadmill score, r = 0.78 (+)  2) Ability to discriminate among various groups: Walking scores declined significantly across decades (age groups; p<0.0001) and were significantly lower for low-active subjects compared to high-active subjects (p<0.0001).  (+) |
| ***Pooled or summary result (overall rating)*** | | ICC ≥ 0.70  (+) | MIC not defined  (?) | N/R | N/R | Hypotheses confirmed  (+) |

ANOVA = Analysis of variance; N/R = Not reported

Measurement property rating: sufficient (+), insufficient (−), inconsistent (±), indeterminate (?)
