# Supplementary material for: Measures of attributes of locomotor capacity in older people: a systematic literature review following the COSMIN methodology
Source: Age Ageing. 2023 Oct 30;52(Suppl 4):iv44–66. doi: 10.1093/ageing/afad139 (PMC10615073; doi:10.1093/ageing/afad139)
Supplement: aa-23-0360-File006_afad139 [file aa-23-0360-file006_afad139.docx]

World Health Organisation: *Measurements of Healthy Ageing*

**Measures of attributes of locomotor capacity in older people: A systematic literature review following the COSMIN methodology**

**SUPPLEMENTARY DATA**

# Appendix 5: Methodological quality of studies on tools for attributes of locomotor capacity

- Appendix 5.a: Methodological quality of studies on tools for Balance assessment
- Appendix 5.b: Methodological quality of studies on tools for Muscle strength assessment
- Appendix 5.c: Methodological quality of studies on tools for Muscle power assessment
- Appendix 5.d: Methodological quality of studies on tools for Endurance assessment

*RoB Scores and colors: V= very good; A = adequate; D = doubtful; I = inadequate;*

**Appendix 5.a**: Methodological quality of studies on tools for Balance assessment

| **Instrument** | **Studies** | **Reliability** | | | **Validity** | | | | **Responsiveness** |
| --- | --- | --- | --- | --- | --- | --- | --- | --- | --- |
|  |  | ***Reliability*** | ***Measurement error*** | ***Internal consistency*** | ***Criterion validity*** | ***Hypothesis testing for construct validity*** | | ***Structural validity*** | ***Responsiveness*** |
|  |  |  |  |  |  | ***Convergent validity*** | ***Discriminative validity*** |  |  |
| The Balance Evaluation Systems Test (BESTest) | Anson, 2019 [1] | D | D |  |  |  | V |  |  |
|  | Marques, 2016 [2] | D | D |  | V | V | V |  |  |
|  | O'Hoski, 2015 [3] |  |  |  |  | V | V |  |  |
|  | Viveiro, 2019 [4] | V | V |  | V | V | V |  |  |
|  | Wang-Hsu, 2018 [5] | A | A |  |  |  |  |  |  |
|  | Yingyongyudha, 2016 [6] |  |  |  |  |  | V |  |  |
|  |  |  |  |  |  |  |  |  |  |
| The ***Spanish version*** of the BESTest (Spanish BESTest) | Dominguez-Olivan, 2020 [7] | A | A | V | V | V |  |  |  |
|  |  |  |  |  |  |  |  |  |  |
| The Mini-Balance Evaluation Systems Test  (Mini-BESTest) | Anson, 2019 [1] | D | D |  |  |  | V |  |  |
|  | Marques, 2016 [2] | D | D |  | V | V | V |  |  |
|  | O'Hoski, 2015 [3] |  |  |  |  | V | V |  |  |
|  | Viveiro, 2019 [4] | V | V |  | V | V | V |  |  |
|  | Yingyongyudha, 2016 [6] |  |  |  |  |  | V |  |  |
|  |  |  |  |  |  |  |  |  |  |
| The ***Spanish version*** of the Mini-BESTest (Spanish Mini-BESTest) | Dominguez-Olivan, 2020 [7] | A | A | V | V | V |  |  |  |
|  |  |  |  |  |  |  |  |  |  |
| The modified Clinical test of Sensory Interaction in Balance (mCTSIB) | Antoniadou, 2020 [8] | A |  |  |  | V |  |  |  |
|  |  |  |  |  |  |  |  |  |  |
| The Berg Balance Scale (BBS) | Berg, 1992a [9] | V |  |  | V | V | V |  |  |
|  | Berg, 1992b [10] |  |  |  | V | D | V |  |  |
|  | Bogle Thorbahn, 1996 [11] | A |  |  | V | D | V |  |  |
|  | Harada, 1995 [12] |  |  |  | V |  |  |  |  |
|  | Holbein-Jenny, 2005 [13] | A |  | V |  | V |  |  |  |
|  | Marques, 2016 [2] | D | D |  |  | V | V |  |  |
|  | Muir, 2008 [14] |  |  |  | V |  |  |  |  |
|  | Pelicioni, 2022 [15] | A |  |  |  | D |  |  |  |
|  | Viveiro, 2019 [4] | V | V |  | V | V | V |  |  |
|  | Wang, 2006 [16] | V |  | V |  | V | V |  |  |
|  | Yingyongyudha, 2016 [6] |  |  |  |  |  | V |  |  |
|  |  |  |  |  |  |  |  |  |  |
| The ***Brazilian version*** of the Berg balance scale (Brazilian BBS) | Miyamoto, 2004 [17] | A |  |  |  |  |  |  |  |
|  |  |  |  |  |  |  |  |  |  |
| The Lateral Reach (LR) Test | Brauer, 1999 [18] | A |  |  | V |  |  |  |  |
|  |  |  |  |  |  |  |  |  |  |
| The Six-Spot Step Test | Brincks, 2021 [19] | D | D |  |  | V |  |  |  |
|  |  |  |  |  |  |  |  |  |  |
| The Functional reach (FR) test | Brooks, 2006 [20] |  |  |  |  |  | V |  | D |
|  | Galhardas, 2020 [21] | A | A |  |  |  |  |  |  |
|  | Giorgetti, 1998 [22] | A |  |  |  |  |  |  |  |
|  | Lin, 2004 [23] | D |  |  | V | V | V |  | A |
|  |  |  |  |  |  |  |  |  |  |
| Gait Initiation Assessment | Chang, 1999 [24] |  |  |  | V |  |  |  |  |
|  |  |  |  |  |  |  |  |  |  |
| The modified Wii Fit balance board | Chang, 2013 [25] | A |  |  |  |  |  |  |  |
|  |  |  |  |  |  |  |  |  |  |
| The Stepping Threshold Test (STT) | Adams, 2021 [26] |  |  |  |  | V | V |  |  |
|  |  |  |  |  |  |  |  |  |  |
| The Unstable board | Akizuki, 2018 [27] |  |  |  |  | V |  |  |  |
|  |  |  |  |  |  |  |  |  |  |
| The limits of stability (LOS) test | Clark, 1997 [28] | D | A |  |  |  |  |  |  |
|  |  |  |  |  |  |  |  |  |  |
| The Four Square Step Test (FSST) | Cleary, 2017 [29] |  |  |  | V | V | V |  |  |
|  | Işik, 2015 [30] | D |  |  | V | V |  |  |  |
|  | Dite, 2002 [31] | D |  |  | V | V | V |  |  |
|  |  |  |  |  |  |  |  |  |  |
| The mediolateral balance assessment (MELBA) tool | Cofré Lizama, 2015 [32] |  |  |  | V |  |  |  |  |
|  |  |  |  |  |  |  |  |  |  |
| The Spring Scale Test (SST) | DePasquale, 2009 [33] | A | A |  |  | V | V |  |  |
|  |  |  |  |  |  |  |  |  |  |
| The Microsoft Xbox One Kinect (Kinect v2) | Eltoukhy, 2018 [34] | D | D |  | V |  |  |  |  |
|  |  |  |  |  |  |  |  |  |  |
| The TURN 180 test | Fitzpatrick, 2005 [35] | D | D |  |  |  | V |  |  |
|  | Ranji, 2020 [36] |  |  |  | V |  |  |  |  |
|  |  |  |  |  |  |  |  |  |  |
| The Lower Quarter Y-Balance Test (LQ-YBT) | Freund, 2019 [37] | D | D |  |  | V |  |  |  |
|  |  |  |  |  |  |  |  |  |  |
| The Narrow Path Walking Test (NPWT) | Gimmon, 2013  [38] | A | A |  |  | V |  |  |  |
|  |  |  |  |  |  |  |  |  |  |
| One leg standing (OLS) | Giorgetti, 1998 [22] | A |  |  |  |  |  |  |  |
|  | Lin, 2004 [23] | D |  |  | V | V | V |  | A |
|  |  |  |  |  |  |  |  |  |  |
| Tandem Gait (TG) | Giorgetti, 1998 [22] | A |  |  |  |  |  |  |  |
|  |  |  |  |  |  |  |  |  |  |
| The five-times-sit-to-stand test (FTSST) | Goldberg, 2012 [39] | A | A |  |  | V |  |  |  |
|  |  |  |  |  |  |  |  |  |  |
| The Maximum Step Length (MSL) test | Goldberg, 2010 [40] | D | D |  |  | V | V |  |  |
|  |  |  |  |  |  |  |  |  |  |
| The Thirty Rapid-Step test (30-RST) | Goldberg, 2015 [41] | D | D |  |  | V |  |  |  |
|  |  |  |  |  |  |  |  |  |  |
| The Community Balance and Mobility Scale (CBM) | Weber, 2018 [42] | V |  | I |  | V | V |  |  |
|  |  |  |  |  |  |  |  |  |  |
| The ***German***-Community Balance and Mobility Scale (German CBM) | Gordt, 2019 [43] | A |  | I | V | V |  |  |  |
|  |  |  |  |  |  |  |  |  |  |
| The Shortened version of the Community Balance and Mobility Scale (s-CBM) | Gordt, 2020 [44] |  |  | V |  | V | V | V |  |
|  |  |  |  |  |  |  |  |  |  |
| The “Step-Ex” | Halvarsson, 2012 [45] | A | A |  |  |  |  |  |  |
|  |  |  |  |  |  |  |  |  |  |
| Tinetti's POMA balance subscale | Harada, 1995 [12] |  |  |  | V |  |  |  |  |
|  | Lin, 2004 [23] | D |  |  | V | V | V |  | A |
|  |  |  |  |  |  |  |  |  |  |
| The Short Berg Balance Scale (BBS-9) | Hohtari-Kivimaki, 2012 [46] |  |  | V | V |  |  | V |  |
|  |  |  |  |  |  |  |  |  |  |
| The Multi-Directional Reach Test (MDRT) | Holbein-Jenny, 2005 [13] | A |  | D | V | V |  |  |  |
|  | Newton, 2001  [47] | D |  | D | V | V | V |  |  |
|  |  |  |  |  |  |  |  |  |  |
| The Kinect system (Kinect for Xbox 360™, Microsoft Corp, Seattle, WA, USA) | Hsiao, 2018 [48] | A |  |  | V | V |  |  |  |
|  |  |  |  |  |  |  |  |  |  |
| The ***Turkish version*** of Fullerton Advanced Balance (FAB-T) scale | Iyigun, 2018 [49] | D |  |  | V |  |  |  |  |
|  |  |  |  |  |  |  |  |  |  |
| The Fullerton Advanced  Balance (FAB) Scale | Klein, 2011  [50] |  |  |  |  |  |  | A |  |
|  | Rose, 2006 [51] | D |  |  | V |  |  |  |  |
|  |  |  |  |  |  |  |  |  |  |
| The parallel walk test | Lark, 2009 [52] |  |  |  |  | A | V |  |  |
|  |  |  |  |  |  |  |  |  |  |
| The timed up and go (TUG) test | Galhardas, 2020 [21] | A | A |  |  |  |  |  |  |
|  | Lin, 2004 [23] | D |  |  | V | V | V |  | A |
|  | Nightingale, 2019 [53] |  |  |  |  | D |  |  |  |
|  | Pelicioni, 2022 [15] | A |  |  |  | D |  |  |  |
|  | Yingyongyudha, 2016 [6] |  |  |  |  |  | V |  |  |
|  |  |  |  |  |  |  |  |  |  |
| The Balance Computerized Adaptive Testing (Balance CAT) | Lu, 2015 [54] | D |  |  | V |  | V |  |  |
|  |  |  |  |  |  |  |  |  |  |
| The MyBalance test | Mansson, 2021 [55] |  |  |  |  | V |  |  |  |
|  |  |  |  |  |  |  |  |  |  |
| The Brief‐Balance Evaluation Systems Test (Brief‐BESTest) | Marques, 2016 [2] | D | D |  | V | V | V |  |  |
|  | O'Hoski, 2015 [3] |  |  |  |  | V | V |  |  |
|  | Viveiro, 2019 [4] | V | V |  | V | V | V |  |  |
|  |  |  |  |  |  |  |  |  |  |
| The Functional Gait Assessment-***Brazil***  (FGA- Brazil) | Marques, 2021 [56] |  |  |  | V |  | V | A |  |
|  | Kirkwood, 2021 [57] | A | A | V |  |  |  |  |  |
|  |  |  |  |  |  |  |  |  |  |
| The ‘‘Get-up and Go’’ Test | Mathias, 1986 [58] | D |  |  | V |  |  |  |  |
|  |  |  |  |  |  |  |  |  |  |
| The apparatus for assessment of postural responses | Matjacic, 2010  [59] |  |  |  | V |  | V |  |  |
|  |  |  |  |  |  |  |  |  |  |
| A comprehensive set of inertial sensor measures of postural sway | Mcmanus, 2022 [60] | A |  |  |  | V | V |  |  |
|  |  |  |  |  |  |  |  |  |  |
| The Modified Version of the Community Balance and Mobility Scale (CBMS-Home) | Ng, 2021 [61] | A | A | V |  | V |  | I |  |
|  |  |  |  |  |  |  |  |  |  |
| The Pavia Instrumented Tinetti Test (PITT) | Panella, 2008 [62] |  |  | V | V | V | V |  |  |
|  |  |  |  |  |  |  |  |  |  |
| The Dynamic Gait Index (DGI) | Pelicioni, 2022 [15] | A |  |  |  | D |  |  |  |
|  |  |  |  |  |  |  |  |  |  |
| The ***Danish Version*** of the Dynamic Gait Index (Danish DGI) | Jønsson, 2011 [63] | V | V |  |  |  |  |  |  |
|  |  |  |  |  |  |  |  |  |  |
| The Functional Gait  Assessment (FGA) | Pelicioni, 2022 [15] | D |  |  |  | D |  |  |  |
|  | Wrisley, 2010 [64] |  |  |  | V | V | V |  |  |
|  | Beninato, 2016 [65] |  |  |  |  |  |  | A |  |
|  |  |  |  |  |  |  |  |  |  |
| The NIH Toolbox^®^ Standing Balance Test | Peller, 2022 [66] | A | A |  | V |  |  |  |  |
|  |  |  |  |  |  |  |  |  |  |
| The Biodex SD (Biodex Medical Systems, Shirley NY) | Riemann, 2017 [67] | A | A |  |  |  |  |  |  |
|  |  |  |  |  |  |  |  |  |  |
| The Balance Scale (by Roberts) | Roberts, 1987 [68] |  |  | I |  |  |  | D |  |
|  |  |  |  |  |  |  |  |  |  |
| The ***Turkish Version*** of the Berg Balance Scale (BBS) | Sahin, 2008 [69] | A |  | I |  | V |  | I |  |
|  |  |  |  |  |  |  |  |  |  |
| The ***Persian version*** of the Berg Balance Scale (BBS) | Salavati, 2012 [70] | A |  | I |  | V |  |  |  |
|  |  |  |  |  |  |  |  |  |  |
| The Nintendo Wii Fit *exergame* | Sato, 2021 [71] |  |  |  |  | V |  |  |  |
|  |  |  |  |  |  |  |  |  |  |
| The Wii Stillness (WST) Test | Simms, 2020 [72] |  |  |  | V |  |  |  |  |
|  |  |  |  |  |  |  |  |  |  |
| The short form of the Fullerton Advanced Balance (SF-FAB) scale | Sinaei, 2021 [73] | V | V | V | V |  | V |  |  |
|  |  |  |  |  |  |  |  |  |  |
| The 'balance meter' | Stokes, 1998  [74] | D | D |  |  | V | V |  |  |
|  |  |  |  |  |  |  |  |  |  |
| The AMTI Accusway system for balance and postural sway measurement | Swanenburg, 2008 [75] | A | A |  |  |  |  |  |  |
|  |  |  |  |  |  |  |  |  |  |
| A dual-task computer game-based platform (TGP) | Szturm, 2015 [76] | A | A |  |  |  |  |  |  |
|  |  |  |  |  |  |  |  |  |  |
| The Modified Bathroom Scale | Vermeulen, 2012 [77] |  |  |  |  | V | V |  |  |
|  |  |  |  |  |  |  |  |  |  |
| The instrumented modified Clinical Test of Sensory Interaction on Balance (i-mCTSIB) | Watson, 2021 [78] | A | A |  |  |  |  |  |  |
|  |  |  |  |  |  |  |  |  |  |
| Models for estimating decline in balance using accelerometry-based gait features | Simila, 2017 [79] |  |  |  | I | A |  |  |  |
|  |  |  |  |  |  |  |  |  |  |
| The FICSIT Balance Scales (FICSIT-3 and FICSIT-4) | Rossiter-Fornoff, 1995 [80] | I |  |  |  | D | D |  |  |
|  |  |  |  |  |  |  |  |  |  |
| The Wii Balance Board™ (WBB) | Olvera-Chavez, 2013 [81] |  |  |  |  | V |  |  |  |
|  | Scaglioni-Solano, 2014 [82] | A | A |  | I |  |  |  |  |
|  |  |  |  |  |  |  |  |  |  |
| The Balance Tracking System (BTrackS) | Levy, 2018 [83] | A | A | I | V |  |  |  |  |
|  |  |  |  |  |  |  |  |  |  |
| The NeuroCom Smart Equitest Research System | Harro, 2019 [84] | A | A |  |  | V | V |  |  |

**Appendix 5.b**: Methodological quality of studies on tools for Muscle strength assessment

| **Instrument** | **Studies** | **Reliability** | | | **Validity** | | | | **Responsiveness** |
| --- | --- | --- | --- | --- | --- | --- | --- | --- | --- |
|  |  | ***Reliability*** | ***Measurement error*** | ***Internal consistency*** | ***Criterion validity*** | ***Hypothesis testing for construct validity*** | | ***Structural validity*** | ***Responsiveness*** |
|  |  |  |  |  |  | ***Convergent validity*** | ***Discriminative validity*** |  |  |
|  |  |  |  |  |  |  |  |  |  |
| The JAMAR hand-held hydraulic dynamometer | Abizanda, 2012  [85] | A | D |  |  | V |  |  |  |
|  | Silva, 2019 [86] | A | A |  |  |  |  |  |  |
|  |  |  |  |  |  |  |  |  |  |
| A uni-axial load cell device | Alqahtani, 2019 [87] | A | A |  |  | V |  |  |  |
|  |  |  |  |  |  |  |  |  |  |
| The calf-raise senior (CRS) test | Andre, 2016 [88] | A | A |  | V |  | V |  |  |
|  |  |  |  |  |  |  |  |  |  |
| The Handheld Dynamometry (HHD): The Lafayette Manual Muscle Tester, Model # 01163, (Lafayette Instrument Inc., Lafayette, Indiana) | Arnold, 2010 [89] | A | A |  | V |  |  |  |  |
|  | Bohannon, 2005 [90] | I | I |  |  |  |  |  |  |
|  | Bohannon, 1997 [91] |  |  | V |  |  |  |  |  |
|  | Martin, 2006 [92] |  |  |  | V |  |  |  |  |
|  |  |  |  |  |  |  |  |  |  |
| The Nintendo Wii Balance Board (WBB) | Blomkvist, 2016 [93] | A | A |  |  | V |  |  |  |
|  | Jorgensen, 2015 [94] | A | A |  |  | A |  |  |  |
|  |  |  |  |  |  |  |  |  |  |
| The Modified Sphygmomanometer Test (MST) | Brito, 2022  [95] | V | V |  |  | V |  |  |  |
|  |  |  |  |  |  |  |  |  |  |
| MicroFET2 hand-held dynamometer | Buckinx, 2017 [96] | A | A |  |  |  |  |  |  |
|  |  |  |  |  |  |  |  |  |  |
| The isometric knee extension (IKE) test (IKE test + strain gauge) | Buendía-Romero, 2021 [97] | A | A |  |  |  |  |  |  |
|  |  |  |  |  |  |  |  |  |  |
| The Q Force | Douma, 2016 [98] | A | A |  |  |  |  |  |  |
|  |  |  |  |  |  |  |  |  |  |
| An analog dynamometer (SENSIX®, Poitiers, France) coupled with the DELSYS System | Gafner, 2017 [99] | A | A |  |  |  |  |  |  |
|  |  |  |  |  |  |  |  |  |  |
| The Biodex System 3 isokinetic dynamometer (Biodex Medical Systems, Shirley, N.Y., USA) | Hartmann, 2009 [100] | A | A |  |  |  |  |  |  |
|  | Symons, 2004 [101] | A | D |  |  |  |  |  |  |
|  |  |  |  |  |  |  |  |  |  |
| The Isokinetic dynamometer (KinCom 500H, Chattecx Corp., Hixson, TN, USA) | Holsgaard Larsen, 2007  [102] | D | D |  |  |  |  |  |  |
|  |  |  |  |  |  |  |  |  |  |
| The Leg Press Sled (LPS) | Hutchison, 2006 [103] | D | D |  | V |  |  |  |  |
|  |  |  |  |  |  |  |  |  |  |
| The Microfet 2000 strain gauge portable dynamometer (PD) | Karner, 1998 [104] | A |  |  |  |  |  |  |  |
|  |  |  |  |  |  |  |  |  |  |
| A load cell setup | Keshavarzi, 2022 [105] | A | A |  |  |  |  |  |  |
|  |  |  |  |  |  |  |  |  |  |
| The push-off test (POT) | Legg, 2020 [106] | A | A |  |  | V |  |  |  |
|  |  |  |  |  |  |  |  |  |  |
| The functional multi-joint isokinetic dynamometer | Legg, 2020 [106] | A | A |  |  | V |  |  |  |
|  |  |  |  |  |  |  |  |  |  |
| The MyBalance test | Mansson, 2021 [55] |  |  |  |  | V |  |  |  |
|  |  |  |  |  |  |  |  |  |  |
| The maximal isometric strength test of the trunk | Mesquita, 2019 [107] | A | A |  |  |  |  |  |  |
|  |  |  |  |  |  |  |  |  |  |
| The one-repetition maximum (1 RM) using elastic resistance bands test | Nyberg, 2014 [108] |  |  |  | V |  |  |  |  |
|  |  |  |  |  |  |  |  |  |  |
| The lateral step (LS) test | Porto, 2020 [109] | A |  |  | V |  | V |  |  |
|  |  |  |  |  |  |  |  |  |  |
| Tandem Gait (TG) | Porto, 2020 [109] | A |  |  | V |  | V |  |  |
|  |  |  |  |  |  |  |  |  |  |
| Single-leg stance (SS) test | Porto, 2020 [109] |  |  |  | V |  | V |  |  |
|  |  |  |  |  |  |  |  |  |  |
| The one repetition maximum (1 RM) using a muscle strength training device for the arm/shoulder | Rydwik, 2007 [110] | D | A |  |  |  | V |  |  |
|  |  |  |  |  |  |  |  |  |  |
| The five-repetition sit-to-stand (STS) test | Schaubert, 2005 [111] | I | I |  |  |  |  |  |  |
|  |  |  |  |  |  |  |  |  |  |
| A standardized heel-rise test (Using trunk accelerometry) | Schmid, 2011 [112] | A | A |  |  | D |  |  |  |
|  |  |  |  |  |  |  |  |  |  |
| The one-repetition maximum (1 RM) performed on the Keiser A-300 pneumatic equipment | Schroeder, 2007 [113] | D | D |  |  |  |  |  |  |
|  |  |  |  |  |  |  |  |  |  |
| Grip strength, measured using a Smedley-type dynamometer | Suzuki, 2019 [114] | D | D |  |  |  |  |  |  |
|  |  |  |  |  |  |  |  |  |  |
| Knee extension strength, measured using a handheld dynamometer (μ-Tas F-1; Anima Inc., Tokyo, Japan) | Suzuki, 2019 [114] | D | D |  |  |  |  |  |  |
|  |  |  |  |  |  |  |  |  |  |
| The 30-s Chair-Stand Test | Jones, 1999 [115] | A |  |  |  | A | V |  |  |

**Appendix 5.c**: Methodological quality of studies on tools for Muscle power assessment

| **Instrument** | **Studies** | **Reliability** | | | **Validity** | | | | **Responsiveness** |
| --- | --- | --- | --- | --- | --- | --- | --- | --- | --- |
|  |  | ***Reliability*** | ***Measurement error*** | ***Internal consistency*** | ***Criterion validity*** | ***Hypothesis testing for construct validity*** | | ***Structural validity*** | ***Responsiveness*** |
|  |  |  |  |  |  | ***Convergent validity*** | ***Discriminative validity*** |  |  |
| The 30-s sit-to-stand (STS) muscle power test | Alcazar, 2020 [116] |  |  |  | V |  |  |  |  |
|  |  |  |  |  |  |  |  |  |  |
| The sit-to-stand (STS) muscle power test | Alcazar, 2018 [117] |  |  |  |  | A |  |  |  |
|  |  |  |  |  |  |  |  |  |  |
| The sit-to-stand power test (STSp), using a portable linear transducer | Balachandran, 2021 [118] | A | A |  |  | A | V |  |  |
|  |  |  |  |  |  |  |  |  |  |
| The Vertical jump (VJ) measured by a contact mat | Farias, 2013 [119] | A | A |  |  |  |  |  |  |
|  |  |  |  |  |  |  |  |  |  |
| The Tendo Weightlifting Analyzer (Trencin, Slovak Republic) | Gray, 2014 [120] | D |  |  |  | A |  |  |  |
|  |  |  |  |  |  |  |  |  |  |
| Counter-movement jump (CMJ) test performed on a force platform | Holsgaard Larsen, 2007 [102] | D | D |  |  |  |  |  |  |
|  |  |  |  |  |  |  |  |  |  |
| The chair stand mean power (CSMP) test, using the Fitro Dyne device (Fitronic S. R. O. Co, Slovakia). | Kato, 2015 [121] | A |  |  |  |  | V |  |  |
|  |  |  |  |  |  |  |  |  |  |
| The sit-to-stand (STS) performance power using a linear encoder | Lindemann, 2015 [122] |  |  |  | V |  |  |  |  |
|  |  |  |  |  |  |  |  |  |  |
| The Jumping Mechanography | Rittweger, 2004 [123] | D | D |  |  | A |  |  |  |
|  |  |  |  |  |  |  |  |  |  |
| A standardized heel-rise test (Using trunk accelerometry) | Schmid, 2011 [112] | A | A |  |  | D |  |  |  |
|  |  |  |  |  |  |  |  |  |  |
| Unilateral leg extension power (W) using the Bassey Power Rig | Schroeder, 2007 [113] | D | D |  |  |  |  |  |  |
|  |  |  |  |  |  |  |  |  |  |
| The Ramp Power Test | Signorile, 2007 [124] | A | D |  |  | A |  |  |  |

**Appendix 5.d**: Methodological quality of studies on tools for Endurance assessment

| **Instrument** | **Study** | **Reliability** | | | **Validity** | | | | **Responsiveness** |
| --- | --- | --- | --- | --- | --- | --- | --- | --- | --- |
|  |  | ***Reliability*** | ***Measurement error*** | ***Internal consistency*** | ***Criterion validity*** | ***Hypothesis testing for construct validity*** | | ***Structural validity*** | ***Responsiveness*** |
|  |  |  |  |  |  | ***Convergent validity*** | ***Discriminative validity*** |  |  |
| The 6-Minute Walk Test | Rikli, 1998 [125] | A | D |  |  | A | V |  |  |

1. Anson E, Thompson E, Ma L, Jeka J. Reliability and Fall Risk Detection for the BESTest and Mini-BESTest in Older Adults. Journal of geriatric physical therapy (2001). 2019;42(2):81-5. doi: <https://dx.doi.org/10.1519/JPT.0000000000000123>.

2. Marques A, Almeida S, Carvalho J, Cruz J, Oliveira A, Jacome C. Reliability, Validity, and Ability to Identify Fall Status of the Balance Evaluation Systems Test, Mini-Balance Evaluation Systems Test, and Brief-Balance Evaluation Systems Test in Older People Living in the Community. Archives of physical medicine and rehabilitation. 2016;97(12):2166-73.e1. doi: <https://dx.doi.org/10.1016/j.apmr.2016.07.011>.

3. O'Hoski S, Sibley KM, Brooks D, Beauchamp MK. Construct validity of the BESTest, mini-BESTest and briefBESTest in adults aged 50 years and older. Gait & posture. 2015;42(3):301-5. doi: <https://dx.doi.org/10.1016/j.gaitpost.2015.06.006>.

4. Viveiro LAP, Gomes GCV, Bacha JMR, Carvas Junior N, Kallas ME, Reis M, et al. Reliability, Validity, and Ability to Identity Fall Status of the Berg Balance Scale, Balance Evaluation Systems Test (BESTest), Mini-BESTest, and Brief-BESTest in Older Adults Who Live in Nursing Homes. Journal of geriatric physical therapy (2001). 2019;42(4):E45-E54. doi: <https://dx.doi.org/10.1519/JPT.0000000000000215>.

5. Wang-Hsu E, Smith SS. Interrater and Test-Retest Reliability and Minimal Detectable Change of the Balance Evaluation Systems Test (BESTest) and Subsystems With Community-Dwelling Older Adults. Journal of geriatric physical therapy (2001). 2018;41(3):173-9. doi: <https://dx.doi.org/10.1519/JPT.0000000000000117>.

6. Yingyongyudha A, Saengsirisuwan V, Panichaporn W, Boonsinsukh R. The Mini-Balance Evaluation Systems Test (Mini-BESTest) Demonstrates Higher Accuracy in Identifying Older Adult Participants With History of Falls Than Do the BESTest, Berg Balance Scale, or Timed Up and Go Test. Journal of geriatric physical therapy (2001). 2016;39(2):64-70. doi: <https://dx.doi.org/10.1519/JPT.0000000000000050>.

7. Dominguez-Olivan P, Gasch-Gallen A, Aguas-Garcia E, Bengoetxea A. Validity and reliability testing of the Spanish version of the BESTest and mini-BESTest in healthy community-dwelling elderly. BMC geriatrics. 2020;20(1):444. doi: <https://dx.doi.org/10.1186/s12877-020-01724-3>.

8. Antoniadou E, Kalivioti X, Stolakis K, Koloniari A, Megas P, Tyllianakis M, et al. Reliability and validity of the mCTSIB dynamic platform test to assess balance in a population of older women living in the community. Journal of musculoskeletal & neuronal interactions. 2020;20(2):185-93.

9. Berg KO, Maki BE, Williams JI, Holliday PJ, Wood-Dauphinee SL. Clinical and laboratory measures of postural balance in an elderly population. Archives of physical medicine and rehabilitation. 1992;73(11):1073-80.

10. Berg KO, Wood-Dauphinee SL, Williams JI, Maki B. Measuring balance in the elderly: validation of an instrument. Canadian journal of public health = Revue canadienne de sante publique. 1992;83 Suppl 2(ck6, 0372714):S7-11.

11. Bogle Thorbahn LD, Newton RA. Use of the Berg Balance Test to predict falls in elderly persons. Physical therapy. 1996;76(6):576-5.

12. Harada N, Chiu V, Damron-Rodriguez J, Fowler E, Siu A, Reuben DB. Screening for balance and mobility impairment in elderly individuals living in residential care facilities. Physical therapy. 1995;75(6):462-9.

13. Holbein-Jenny MA, Billek-Sawhney B, Beckman E, Smith T. Balance in personal care home residents: a comparison of the Berg Balance Scale, the Multi-Directional Reach Test, and the Activities-Specific Balance Confidence Scale. Journal of geriatric physical therapy (2001). 2005;28(2):48-53.

14. Muir SW, Berg K, Chesworth B, Speechley M. Use of the Berg Balance Scale for predicting multiple falls in community-dwelling elderly people: a prospective study. Physical therapy. 2008;88(4):449-59. doi: <https://dx.doi.org/10.2522/ptj.20070251>.

15. Pelicioni PHS, Waters DL, Still A, Hale L. A pilot investigation of reliability and validity of balance and gait assessments using telehealth with healthy older adults. Experimental Gerontology. 2022;162. doi: 10.1016/j.exger.2022.111747.

16. Wang C-Y, Hsieh C-L, Olson SL, Wang C-H, Sheu C-F, Liang C-C. Psychometric properties of the Berg Balance Scale in a community-dwelling elderly resident population in Taiwan. Journal of the Formosan Medical Association = Taiwan yi zhi. 2006;105(12):992-1000.

17. Miyamoto ST, Lombardi Junior I, Berg KO, Ramos LR, Natour J. Brazilian version of the Berg balance scale. Brazilian journal of medical and biological research = Revista brasileira de pesquisas medicas e biologicas. 2004;37(9):1411-21.

18. Brauer S, Burns Y, Galley P. Lateral reach: a clinical measure of medio-lateral postural stability. Physiotherapy research international : the journal for researchers and clinicians in physical therapy. 1999;4(2):81-8.

19. Brincks J, Callesen J. Examining the test-retest reliability and construct validity of the Six-Spot Step Test in older adults with self-reported balance problems. Clinical rehabilitation. 2021;35(10):1478-87. doi: <https://dx.doi.org/10.1177/02692155211010278>.

20. Brooks D, Davis AM, Naglie G. Validity of 3 physical performance measures in inpatient geriatric rehabilitation. Archives of Physical Medicine & Rehabilitation. 2006;87(1):105-10. doi: 10.1016/j.apmr.2005.08.109.

21. Galhardas L, Raimundo A, Marmeleira J. Test-retest reliability of upper-limb proprioception and balance tests in older nursing home residents. Archives of gerontology and geriatrics. 2020;89(8214379, 7ax):104079. doi: <https://dx.doi.org/10.1016/j.archger.2020.104079>.

22. Giorgetti MM, Harris BA, Jette A. Reliability of clinical balance outcome measures in the elderly. Physiotherapy research international : the journal for researchers and clinicians in physical therapy. 1998;3(4):274-83.

23. Lin M-R, Hwang H-F, Hu M-H, Wu H-DI, Wang Y-W, Huang F-C. Psychometric Comparisons of the Timed Up and Go, One-Leg Stand, Functional Reach, and Tinetti Balance Measures in Community-Dwelling Older People. Journal of the American Geriatrics Society. 2004;52(8):1343-8. doi: <https://dx.doi.org/10.1111/j.1532-5415.2004.52366.xhttps://dx.doi.org/10.1111/j.1532-5415.2004.52366.x>.

24. Chang H, Krebs DE. Dynamic balance control in elders: gait initiation assessment as a screening tool. Archives of physical medicine and rehabilitation. 1999;80(5):490-4.

25. Chang W-D, Chang W-Y, Lee C-L, Feng C-Y. Validity and reliability of wii fit balance board for the assessment of balance of healthy young adults and the elderly. Journal of physical therapy science. 2013;25(10):1251-3. doi: <https://dx.doi.org/10.1589/jpts.25.1251>.

26. Adams M, Brull L, Lohkamp M, Schwenk M. The Stepping Threshold Test for Reactive Balance: Validation of Two Observer-Based Evaluation Strategies to Assess Stepping Behavior in Fall-Prone Older Adults. Frontiers in sports and active living. 2021;3(101765780):715392. doi: <https://dx.doi.org/10.3389/fspor.2021.715392>.

27. Akizuki K, Echizenya Y, Kaneno T, Yabuki J, Ohashi Y. Dynamic balance assessment using an unstable board in community-dwelling elderly people. Journal of physical therapy science. 2018;30(8):1086-91. doi: <https://dx.doi.org/10.1589/jpts.30.1086>.

28. Clark S, Rose DJ, Fujimoto K. Generalizability of the limits of stability test in the evaluation of dynamic balance among older adults. Archives of physical medicine and rehabilitation. 1997;78(10):1078-84.

29. Cleary K, Skornyakov E. Predicting falls in older adults using the four square step test. Physiotherapy theory and practice. 2017;33(10):766-71. doi: <https://dx.doi.org/10.1080/09593985.2017.1354951>.

30. Işik Eİ, Altuğ F, Cavlak U. Reliability and validity of four step square test in older adults. Turk Geriatri Dergisi. 2015;18(2):151-5.

31. Dite W, Temple VA. A clinical test of stepping and change of direction to identify multiple falling older adults. Archives of physical medicine and rehabilitation. 2002;83(11):1566-71.

32. Cofre Lizama LE, Pijnappels M, Rispens SM, Reeves NP, Verschueren SM, van Dieen JH. Mediolateral balance and gait stability in older adults. Gait & posture. 2015;42(1):79-84. doi: <https://dx.doi.org/10.1016/j.gaitpost.2015.04.010>.

33. DePasquale L, Toscano L. The Spring Scale Test: a reliable and valid tool for explaining fall history. Journal of geriatric physical therapy (2001). 2009;32(4):159-67.

34. Eltoukhy MA, Kuenze C, Oh J, Signorile JF. Validation of Static and Dynamic Balance Assessment Using Microsoft Kinect for Young and Elderly Populations. IEEE journal of biomedical and health informatics. 2018;22(1):147-53. doi: <https://dx.doi.org/10.1109/JBHI.2017.2686330>.

35. Fitzpatrick C, Simpson JM, Valentine JD, Ryder S, Peacock-Edwards T, Sidnell P, et al. The measurement properties and performance characteristics among older people of TURN180, a test of dynamic postural stability. Clinical rehabilitation. 2005;19(4):412-8.

36. Ranji KV, Sam Thamburaj A, Raj JO, Ahmed SZ, Arul B. Prediction of falls in elderly: Correlation of Berg’s balance scale with turn 180 test. International Journal of Research in Pharmaceutical Sciences. 2020;11(4):6949-53. doi: 10.26452/IJRPS.V11I4.3698.

37. Freund JE, Stetts DM, Oostindie A, Shepherd J, Vallabhajosula S. Lower Quarter Y-Balance Test in healthy women 50-79 years old. Journal of women & aging. 2019;31(6):475-91. doi: <https://dx.doi.org/10.1080/08952841.2018.1510248>.

38. Gimmon Y, Jacob G, Lenoble-Hoskovec C, Bula C, Melzer I. Relative and absolute reliability of the clinical version of the Narrow Path Walking Test (NPWT) under single and dual task conditions. Archives of gerontology and geriatrics. 2013;57(1):92-9. doi: <https://dx.doi.org/10.1016/j.archger.2013.02.001>.

39. Goldberg A, Chavis M, Watkins J, Wilson T. The five-times-sit-to-stand test: validity, reliability and detectable change in older females. Aging clinical and experimental research. 2012;24(4):339-44.

40. Goldberg A, Schepens S, Wallace M. Concurrent validity and reliability of the maximum step length test in older adults. Journal of geriatric physical therapy (2001). 2010;33(3):122-7.

41. Goldberg A, Talley SA. Performance on a test of rapid stepping in community-dwelling older adults: validity, relative and absolute reliability and minimum detectable change. Physiotherapy theory and practice. 2015;31(7):483-8. doi: <https://dx.doi.org/10.3109/09593985.2015.1024805>.

42. Weber M, Van Ancum J, Bergquist R, Taraldsen K, Gordt K, Mikolaizak AS, et al. Concurrent validity and reliability of the Community Balance and Mobility scale in young-older adults. BMC geriatrics. 2018;18(1):156. doi: <https://dx.doi.org/10.1186/s12877-018-0845-9>.

43. Gordt K, Mikolaizak AS, Nerz C, Barz C, Gerhardy T, Weber M, et al. German version of the Community Balance and Mobility Scale : Translation and evaluation of measurement properties. Zeitschrift fur Gerontologie und Geriatrie. 2019;52(1):28-36. doi: <https://dx.doi.org/10.1007/s00391-018-1374-z>.

44. Gordt K, Mikolaizak AS, Taraldsen K, Bergquist R, Van Ancum JM, Nerz C, et al. Creating and Validating a Shortened Version of the Community Balance and Mobility Scale for Application in People Who Are 61 to 70 Years of Age. Physical therapy. 2020;100(1):180-91. doi: <https://dx.doi.org/10.1093/ptj/pzz132>.

45. Halvarsson A, Franzen E, Olsson E, Stahle A. Relative and absolute reliability of the new "Step-Ex" step-execution test in elderly people with and without balance problems. Disability and rehabilitation. 2012;34(23):1986-92.

46. Hohtari-Kivimaki U, Salminen M, Vahlberg T, Kivela S-L. Short Berg Balance Scale - correlation to static and dynamic balance and applicability among the aged. Aging clinical and experimental research. 2012;24(1):42-6.

47. Newton RA. Validity of the multi-directional reach test: A practical measure for limits of stability in older adults. Journals of Gerontology - Series A Biological Sciences and Medical Sciences. 2001;56(4):M248-M52. doi: 10.1093/gerona/56.4.M248.

48. Hsiao M-Y, Li C-M, Lu IS, Lin Y-H, Wang T-G, Han D-S. An investigation of the use of the Kinect system as a measure of dynamic balance and forward reach in the elderly. Clinical rehabilitation. 2018;32(4):473-82. doi: <https://dx.doi.org/10.1177/0269215517730117>.

49. Iyigun G, Kirmizigil B, Angin E, Oksuz S, Can F, Eker L, et al. The reliability and validity of the Turkish version of Fullerton Advanced Balance (FAB-T) scale. Archives of Gerontology and Geriatrics. 2018;78:38-44. doi: 10.1016/j.archger.2018.05.022.

50. Klein PJ, Fiedler RC, Rose DJ. Rasch analysis of the fullerton advanced balance (FAB) scale. Physiotherapy Canada. 2011;63(1):115-25. doi: 10.3138/ptc.2009-51.

51. Rose DJ, Lucchese N, Wiersma LD. Development of a multidimensional balance scale for use with functionally independent older adults. Archives of physical medicine and rehabilitation. 2006;87(11):1478-85.

52. Lark SD, Pasupuleti S. Validity of a functional dynamic walking test for the elderly. Archives of physical medicine and rehabilitation. 2009;90(3):470-4. doi: <https://dx.doi.org/10.1016/j.apmr.2008.08.221>.

53. Nightingale CJ, Mitchell SN, Butterfield SA. Validation of the Timed Up and Go Test for Assessing Balance Variables in Adults Aged 65 and Older. Journal of aging and physical activity. 2019;27(2):230-3. doi: <https://dx.doi.org/10.1123/japa.2018-0049>.

54. Lu W-S, Lien BY-H, Hsieh C-L. Psychometric properties of the Balance Computerized Adaptive Test in residents in long-term care facilities. Archives of gerontology and geriatrics. 2015;61(2):149-53. doi: <https://dx.doi.org/10.1016/j.archger.2015.04.009>.

55. Mansson L, Backman P, Ohberg F, Sandlund J, Selling J, Sandlund M. Evaluation of Concurrent Validity between a Smartphone Self-Test Prototype and Clinical Instruments for Balance and Leg Strength. Sensors (Basel, Switzerland). 2021;21(5). doi: <https://dx.doi.org/10.3390/s21051765>.

56. Marques LBF, Moreira BdS, Ocarino JdM, Sampaio RF, Bastone AdC, Kirkwood RN. Construct and criterion validity of the functional gait assessment-Brazil in community-dwelling older adults. Brazilian journal of physical therapy. 2021;25(2):186-93. doi: <https://dx.doi.org/10.1016/j.bjpt.2020.05.008>.

57. Kirkwood RN, Batista NCL, Marques LBF, de Melo Ocarino J, Neves LLA, de Souza Moreira B. Cross-cultural adaptation and reliability of the Functional Gait Assessment in older Brazilian adults. Braz J Phys Ther. 2021;25(1):78-85. Epub 2020/03/08. doi: 10.1016/j.bjpt.2020.02.004. PubMed PMID: 32143957; PubMed Central PMCID: PMCPMC7817863.

58. Mathias S, Nayak US, Isaacs B. Balance in elderly patients: the "get-up and go" test. Archives of physical medicine and rehabilitation. 1986;67(6):387-9.

59. Matjacic Z, Bohinc K, Cikajlo I. Development of an objective balance assessment method for purposes of telemonitoring and telerehabilitation in elderly population. Disability and rehabilitation. 2010;32(3):259-66. doi: <https://dx.doi.org/10.3109/09638280902943215>.

60. McManus K, Greene BR, Motti Ader LG, Caulfield B. Development of Data-driven Metrics for Balance Impairment and Fall Risk Assessment in Older Adults. IEEE transactions on bio-medical engineering. 2022;PP(gfx, 0012737). doi: <https://dx.doi.org/10.1109/TBME.2022.3142617>.

61. Ng YL, Hill KD, Jacques A, Burton E. Reliability and Validity of a Modified Version of the Community Balance and Mobility Scale (CBMS-Home) for Use in Home Assessment. Physical therapy. 2021;101(8). doi: <https://dx.doi.org/10.1093/ptj/pzab134>.

62. Panella L, Tinelli C, Buizza A, Lombardi R, Gandolfi R. Towards objective evaluation of balance in the elderly: validity and reliability of a measurement instrument applied to the Tinetti test. International journal of rehabilitation research Internationale Zeitschrift fur Rehabilitationsforschung Revue internationale de recherches de readaptation. 2008;31(1):65-72. doi: <https://dx.doi.org/10.1097/MRR.0b013e3282f28f38>.

63. Jonsson LR, Kristensen MT, Tibaek S, Andersen CW, Juhl C. Intra- and interrater reliability and agreement of the Danish version of the Dynamic Gait Index in older people with balance impairments. Arch Phys Med Rehabil. 2011;92(10):1630-5. Epub 2011/08/30. doi: 10.1016/j.apmr.2011.04.020. PubMed PMID: 21872845.

64. Wrisley DM, Kumar NA. Functional gait assessment: concurrent, discriminative, and predictive validity in community-dwelling older adults. Phys Ther. 2010;90(5):761-73. Epub 2010/04/03. doi: 10.2522/ptj.20090069. PubMed PMID: 20360052.

65. Beninato M, Ludlow LH. The Functional Gait Assessment in Older Adults: Validation Through Rasch Modeling. Phys Ther. 2016;96(4):456-68. Epub 2015/09/05. doi: 10.2522/ptj.20150167. PubMed PMID: 26337259.

66. Peller A, Garib R, Garbe E, Komforti D, Joffe C, Magras A, et al. Validity and reliability of the NIH Toolbox® Standing Balance Test As compared to the Biodex Balance System SD. Physiotherapy theory and practice. 2022;((Peller A.; Garib R.; Garbe E.; Komforti D.; Joffe C.; Magras A.; Trapuzzano A.; Stock M.S.; Dawson N.T.) Division of Physical Therapy, School of Kinesiology and Physical Therapy, College of Health Professions and Sciences, Academic Health Sciences Center):1-7. doi: 10.1080/09593985.2022.2027584.

67. Riemann BL, Piersol K. Intersession reliability of self-selected and narrow stance balance testing in older adults. Aging clinical and experimental research. 2017;29(5):1045-8. doi: <https://dx.doi.org/10.1007/s40520-016-0687-2>.

68. Roberts BL, Mueller MG. The Balance Scale: Factor analysis and reliability. Perceptual and Motor Skills. 1987;65(2):367-74. doi: <https://dx.doi.org/10.2466/pms.1987.65.2.367https://dx.doi.org/10.2466/pms.1987.65.2.367>.

69. Sahin F, Yilmaz F, Ozmaden A, Kotevolu N, Sahin T, Kuran B. Reliability and validity of the Turkish version of the Berg Balance Scale. Journal of geriatric physical therapy (2001). 2008;31(1):32-7.

70. Salavati M, Negahban H, Mazaheri M, Soleimanifar M, Hadadi M, Sefiddashti L, et al. The Persian version of the Berg Balance Scale: inter and intra-rater reliability and construct validity in elderly adults. Disability and rehabilitation. 2012;34(20):1695-8. doi: <https://dx.doi.org/10.3109/09638288.2012.660604>.

71. Sato A, Goh A-C. Concurrent and discriminant validity of Nintendo Wii Fit exergame for the assessment of postural sway. Journal of physical therapy science. 2021;33(2):100-5. doi: <https://dx.doi.org/10.1589/jpts.33.100>.

72. Simms AJ, Hernandez LR, Sebastião E. Concurrent validity of the Wii Stillness Test as a measure of balance performance in older adults. Gerontechnology. 2020;19(3):1-5. doi: 10.4017/gt.2020.19.003.04.

73. Sinaei E, Rose DJ, Javadpour S, Yoosefinejad AK. Reliability and Fall-Risk Predictability of the Short Form of the Fullerton Advanced Balance Scale in Iranian Older Adults. Journal of aging and physical activity. 2021;((Sinaei E.; Javadpour S.; Yoosefinejad A.K.) Rehabilitation Sciences Research Center, Shiraz University of Medical Sciences, Shiraz, Iran(Sinaei E.; Javadpour S.) Shiraz Geriatric Research Center, Shiraz University of Medical Sciences, Shiraz, Iran(Rose D):1-8. doi: 10.1123/japa.2021-0137.

74. Stokes EK, Finn AM, Kirkham RJR, Walsh JB, Coakley D. The 'balance meter': investigation of an apparatus to measure postural sway. Health Care in Later Life. 1998;3(3):212-25.

75. Swanenburg J, de Bruin ED, Favero K, Uebelhart D, Mulder T. The reliability of postural balance measures in single and dual tasking in elderly fallers and non-fallers. BMC musculoskeletal disorders. 2008;9(100968565):162. doi: <https://dx.doi.org/10.1186/1471-2474-9-162>.

76. Szturm T, Sakhalkar V, Boreskie S, Marotta JJ, Wu C, Kanitkar A. Integrated testing of standing balance and cognition: Test-retest reliability and construct validity. Gait and Posture. 2015;41(1):146-52. doi: 10.1016/j.gaitpost.2014.09.023.

77. Vermeulen J, Neyens JCL, Spreeuwenberg MD, van Rossum E, Hewson DJ, Duchene J, et al. Construct validity of a modified bathroom scale that can measure balance in elderly people. Journal of the American Medical Directors Association. 2012;13(7):665.e1-5. doi: <https://dx.doi.org/10.1016/j.jamda.2012.06.009>.

78. Watson S, Trudelle-Jackson E. Test-Retest Reliability and Minimal Detectable Change of the Instrumented Modified Clinical Test of Sensory Interaction on Balance in Healthy, Older Adults. Journal of geriatric physical therapy (2001). 2021;44(4):183-8. doi: <https://dx.doi.org/10.1519/JPT.0000000000000274>.

79. Simila H, Immonen M, Ermes M. Accelerometry-based assessment and detection of early signs of balance deficits. Computers in biology and medicine. 2017;85(doc, 1250250):25-32. doi: <https://dx.doi.org/10.1016/j.compbiomed.2017.04.009>.

80. Rossiter-Fornoff JE, Wolf SL, Wolfson LI, Buchner DM, Miller JP, Province MA, et al. A cross-sectional validation study of the FICSIT common data base static balance measures. Journals of Gerontology - Series A Biological Sciences and Medical Sciences. 1995;50(6):M291-M7.

81. Olvera-Chavez A, Garza-Hume C, Gutierrez-Robledo LM, Arango-Lopera VE, Perez-Zepeda MU. A Wii pressure platform to assess balance in the elderly. Gerontechnology. 2013;11(3):452-6. doi: <https://dx.doi.org/10.4017/gt.2013.11.3.003.00https://dx.doi.org/10.4017/gt.2013.11.3.003.00>.

82. Scaglioni-Solano P, Aragon-Vargas LF. Validity and reliability of the Nintendo Wii Balance Board to assess standing balance and sensory integration in highly functional older adults. International journal of rehabilitation research Internationale Zeitschrift fur Rehabilitationsforschung Revue internationale de recherches de readaptation. 2014;37(2):138-43. doi: <https://dx.doi.org/10.1097/MRR.0000000000000046>.

83. Levy SS, Thralls KJ, Kviatkovsky SA. Validity and Reliability of a Portable Balance Tracking System, BTrackS, in Older Adults. J Geriatr Phys Ther. 2018;41(2):102-7. Epub 2016/11/29. doi: 10.1519/JPT.0000000000000111. PubMed PMID: 27893566.

84. Harro CC, Garascia C. Reliability and Validity of Computerized Force Platform Measures of Balance Function in Healthy Older Adults. J Geriatr Phys Ther. 2019;42(3):E57-E66. Epub 2018/01/13. doi: 10.1519/JPT.0000000000000175. PubMed PMID: 29324510.

85. Abizanda P, Navarro JL, Garcia-Tomas MI, Lopez-Jimenez E, Martinez-Sanchez E, Paterna G. Validity and usefulness of hand-held dynamometry for measuring muscle strength in community-dwelling older persons. Archives of Gerontology and Geriatrics. 2012;54(1):21-7. doi: <https://dx.doi.org/10.1016/j.archger.2011.02.006https://dx.doi.org/10.1016/j.archger.2011.02.006>.

86. Silva AG, Cerqueira M, Raquel Santos A, Ferreira C, Alvarelhão J, Queirós A. Inter-rater reliability, standard error of measurement and minimal detectable change of the 12-item WHODAS 2.0 and four performance tests in institutionalized ambulatory older adults. Disability & Rehabilitation. 2019;41(3):366-73. doi: 10.1080/09638288.2017.1393112.

87. Alqahtani BA, Sparto PJ, Whitney SL, Greenspan SL, Perera S, Brach JS. Psychometric properties of lower extremity strength measurements recorded in community settings in independent living older adults. Experimental Aging Research. 2019;45(3):282-92. doi: <https://dx.doi.org/10.1080/0361073X.2019.1609145https://dx.doi.org/10.1080/0361073X.2019.1609145>.

88. Andre H-I, Carnide F, Borja E, Ramalho F, Santos-Rocha R, Veloso AP. Calf-raise senior: a new test for assessment of plantar flexor muscle strength in older adults: protocol, validity, and reliability. Clinical interventions in aging. 2016;11(101273480):1661-74.

89. Arnold CM, Warkentin KD, Chilibeck PD, Magnus CRA. The reliability and validity of handheld dynamometry for the measurement of lower-extremity muscle strength in older adults. Journal of strength and conditioning research. 2010;24(3):815-24. doi: <https://dx.doi.org/10.1519/JSC.0b013e3181aa36b8>.

90. Bohannon RW, Schaubert KL. Test-retest reliability of grip-strength measures obtained over a 12-week interval from community-dwelling elders. Journal of hand therapy : official journal of the American Society of Hand Therapists. 2005;18(4):426-8.

91. Bohannon RW. Internal consistency of manual muscle testing scores. Perceptual and motor skills. 1997;85(2):736-8.

92. Martin HJ, Yule V, Syddall HE, Dennison EM, Cooper C, Aihie Sayer A. Is hand-held dynamometry useful for the measurement of quadriceps strength in older people? A comparison with the gold standard Bodex dynamometry. Gerontology. 2006;52(3):154-9. Epub 2006/04/29. doi: 10.1159/000091824. PubMed PMID: 16645295.

93. Blomkvist AW, Andersen S, de Bruin ED, Jorgensen MG. Isometric hand grip strength measured by the Nintendo Wii Balance Board - a reliable new method. BMC musculoskeletal disorders. 2016;17(100968565):56. doi: <https://dx.doi.org/10.1186/s12891-016-0907-0>.

94. Jorgensen MG, Andersen S, Ryg J, Masud T. Novel Use of the Nintendo Wii Board for Measuring Isometric Lower Limb Strength: A Reproducible and Valid Method in Older Adults. PLoS One. 2015;10(10):e0138660. Epub 2015/10/09. doi: 10.1371/journal.pone.0138660. PubMed PMID: 26444554; PubMed Central PMCID: PMCPMC4596703.

95. Brito SAFd, Santana MdM, Benfica PdA, Aguiar LT, Gomes GdC, Faria CDCdM. The modified sphygmomanometer test for assessment of muscle strength of community-dwelling older adults in clinical practice: reliability and validity. Disability and rehabilitation. 2022;44(1):131-8. doi: <https://dx.doi.org/10.1080/09638288.2020.1758804>.

96. Buckinx F, Croisier J-L, Reginster J-Y, Dardenne N, Beaudart C, Slomian J, et al. Reliability of muscle strength measures obtained with a hand-held dynamometer in an elderly population. Clinical physiology and functional imaging. 2017;37(3):332-40. doi: <https://dx.doi.org/10.1111/cpf.12300>.

97. Buendía-Romero Á, Hernández-Belmonte A, Martínez-Cava A, García-Conesa S, Franco-López F, Conesa-Ros E, et al. Isometric knee extension test: A practical, repeatable, and suitable tool for lower-limb screening among institutionalized older adults. Experimental Gerontology. 2021;155. doi: 10.1016/j.exger.2021.111575.

98. Douma KW, Regterschot GRH, Krijnen WP, Slager GEC, van der Schans CP, Zijlstra W. Reliability of the Q Force; a mobile instrument for measuring isometric quadriceps muscle strength. BMC sports science, medicine & rehabilitation. 2016;8(101605016):4. doi: <https://dx.doi.org/10.1186/s13102-016-0029-x>.

99. Gafner S, Bastiaenen CHG, Terrier P, Punt I, Ferrari S, Gold G, et al. Evaluation of hip abductor and adductor strength in the elderly: a reliability study. European review of aging and physical activity : official journal of the European Group for Research into Elderly and Physical Activity. 2017;14(101284836):5. doi: <https://dx.doi.org/10.1186/s11556-017-0174-6>.

100. Hartmann A, Knols R, Murer K, De Bruin ED. Reproducibility of an isokinetic strength-testing protocol of the knee and ankle in older adults. Gerontology. 2009;55(3):259-68. doi: 10.1159/000172832.

101. Symons TB, Vandervoort AA, Rice CL, Overend TJ, Marsh GD. Reliability of isokinetic and isometric knee-extensor force in older women. Journal of Aging and Physical Activity. 2004;12(4):525-37. doi: 10.1123/japa.12.4.525.

102. Holsgaard Larsen A, Caserotti P, Puggaard L, Aagaard P. Reproducibility and relationship of single-joint strength vs multi-joint strength and power in aging individuals. Scandinavian journal of medicine & science in sports. 2007;17(1):43-53.

103. Hutchison AT, Clarke MSF. An Isometric Strength Testing Device for Use with the Elderly: Validation Compared with Isokinetic Measures. Physical & Occupational Therapy in Geriatrics. 2006;25(2):1-12. doi: <https://dx.doi.org/10.1300/J148v25n02_01https://dx.doi.org/10.1300/J148v25n02_01>.

104. Karner PM, Thompson AL, Connelly DM, Vandervoort AA. Strength testing in elderly women using a portable dynamometer. Physiotherapy Canada. 1998;50(1):35-46.

105. Keshavarzi F, Azadinia F, Talebian S, Rasouli O. Test-retest reliability of a load cell setup, Ito, and timed loaded standing tests for measuring muscle strength and endurance in older adults with and without hyperkyphosis. Musculoskeletal science & practice. 2022;58(101692753):102475. doi: <https://dx.doi.org/10.1016/j.msksp.2021.102475>.

106. Legg HS, Spindor J, Dziendzielowski R, Sharkey S, Lanovaz JL, Farthing JP, et al. The reliability and validity of novel clinical strength measures of the upper body in older adults. Hand Therapy. 2020;25(4):130-8. doi: 10.1177/1758998320957373.

107. Mesquita MMA, Santos MS, Vasconcelos ABS, de Sa CA, Pereira LCD, da Silva-Santos IBM, et al. Reliability of a Test for Assessment of Isometric Trunk Muscle Strength in Elderly Women. Journal of aging research. 2019;2019(101543460):9061839. doi: <https://dx.doi.org/10.1155/2019/9061839>.

108. Nyberg A, Hedlund M, Kolberg A, Alm L, Lindström B, Wadell K. The accuracy of using elastic resistance bands to evaluate muscular strength. European Journal of Physiotherapy. 2014;16(2):104-12. doi: 10.3109/21679169.2014.889746.

109. Porto JM, Cangussu-Oliveira LM, Freire Junior RC, Vieira FT, Martins PP, Bandeira ACL, et al. Diagnostic Accuracy of Clinical Tests for the Indirect Assessment of Hip Abductor Muscle Strength in Community-Dwelling Older Women. Physical therapy. 2020;100(11):1967-76. doi: <https://dx.doi.org/10.1093/ptj/pzaa139>.

110. Rydwik E, Karlsson C, Frandin K, Akner G. Muscle strength testing with one repetition maximum in the arm/shoulder for people aged 75 + - test-retest reliability. Clinical rehabilitation. 2007;21(3):258-65.

111. Schaubert K, Bohannon RW. Reliability of the sit-to-stand test over dispersed test sessions. Isokinetics and Exercise Science. 2005;13(2):119-22. doi: 10.3233/ies-2005-0188.

112. Schmid S, Hilfiker R, Radlinger L. Reliability and validity of trunk accelerometry-derived performance measurements in a standardized heel-rise test in elderly subjects. Journal of rehabilitation research and development. 2011;48(9):1137-44.

113. Schroeder ET, Wang Y, Castaneda-Sceppa C, Cloutier G, Vallejo AF, Kawakubo M, et al. Reliability of maximal voluntary muscle strength and power testing in older men. The journals of gerontology Series A, Biological sciences and medical sciences. 2007;62(5):543-9.

114. Suzuki Y, Kamide N, Kitai Y, Ando M, Sato H, Yoshitaka S, et al. Absolute reliability of measurements of muscle strength and physical performance measures in older people with high functional capacities. European Geriatric Medicine. 2019;10(5):733-40. doi: 10.1007/s41999-019-00218-9.

115. Jones CJ, Rikli RE, Beam WC. A 30-s chair-stand test as a measure of lower body strength in community-residing older adults. Res Q Exerc Sport. 1999;70(2):113-9. Epub 1999/06/25. doi: 10.1080/02701367.1999.10608028. PubMed PMID: 10380242.

116. Alcazar J, Kamper RS, Aagaard P, Haddock B, Prescott E, Ara I, et al. Relation between leg extension power and 30-s sit-to-stand muscle power in older adults: validation and translation to functional performance. Scientific reports. 2020;10(1):16337. doi: <https://dx.doi.org/10.1038/s41598-020-73395-4>.

117. Alcazar J, Losa-Reyna J, Rodriguez-Lopez C, Alfaro-Acha A, Rodriguez-Manas L, Ara I, et al. The sit-to-stand muscle power test: An easy, inexpensive and portable procedure to assess muscle power in older people. Experimental gerontology. 2018;112(epq, 0047061):38-43. doi: <https://dx.doi.org/10.1016/j.exger.2018.08.006>.

118. Balachandran AT, Vigotsky AD, Quiles N, Mokkink LB, Belio MA, Glenn JM. Validity, reliability, and measurement error of a sit-to-stand power test in older adults: A pre-registered study. Experimental gerontology. 2021;145(epq, 0047061):111202. doi: <https://dx.doi.org/10.1016/j.exger.2020.111202>.

119. Farias DL, Teixeira TG, Madrid B, Pinho D, Boullosa DA, Prestes J. Reliability of vertical jump performance evaluated with contact mat in elderly women. Clinical physiology and functional imaging. 2013;33(4):288-92. doi: <https://dx.doi.org/10.1111/cpf.12026>.

120. Gray M, Paulson S. Developing a measure of muscular power during a functional task for older adults. BMC geriatrics. 2014;14(100968548):145. doi: <https://dx.doi.org/10.1186/1471-2318-14-145>.

121. Kato Y, Islam MM, Young KC, Rogers ME, Takeshima N. Threshold of chair stand power necessary to perform activities of daily living independently in community-dwelling older women. Journal of geriatric physical therapy (2001). 2015;38(3):122-6. doi: <https://dx.doi.org/10.1519/JPT.0000000000000036>.

122. Lindemann U, Farahmand P, Klenk J, Blatzonis K, Becker C. Validity of linear encoder measurement of sit-to-stand performance power in older people. Physiotherapy (United Kingdom). 2015;101(3):298-302. doi: 10.1016/j.physio.2014.12.005.

123. Rittweger J, Schiessl H, Felsenberg D, Runge M. Reproducibility of the Jumping Mechanography As a Test of Mechanical Power Output in Physically Competent Adult and Elderly Subjects. Journal of the American Geriatrics Society. 2004;52(1):128-31. doi: 10.1111/j.1532-5415.2004.52022.x.

124. Signorile JF, Sandler D, Kempner L, Stanziano D, Ma F, Roos BA. The ramp power test: A power assessment during a functional task for older individuals. Journals of Gerontology - Series A Biological Sciences and Medical Sciences. 2007;62(11):1266-73. doi: 10.1093/gerona/62.11.1266.

125. Rikli RE, Jones CJ. The Reliability and Validity of a 6-Minute Walk Test as a Measure of Physical Endurance in Older Adults. Journal of Aging and Physical Activity. 1998;6(4):363-75. doi: 10.1123/japa.6.4.363.
