# Supplementary material for: Measures of attributes of locomotor capacity in older people: a systematic literature review following the COSMIN methodology
Source: Age Ageing. 2023 Oct 30;52(Suppl 4):iv44–66. doi: 10.1093/ageing/afad139 (PMC10615073; doi:10.1093/ageing/afad139)
Supplement: aa-23-0360-File007_afad139 [file aa-23-0360-file007_afad139.docx]

World Health Organisation: *Measurements of Healthy Ageing*

**Measures of attributes of locomotor capacity in older people: A systematic literature review following the COSMIN methodology**

**SUPPLEMENTARY DATA**

**Appendix 6: GRADE assessment of findings for Balance tools**

**Question 1**: Should the **Berg Balance Scale (BBS)** be used to screen for balance in older people?

**Question 2**: Should the **Balance Evaluation Systems Test (BESTest)** be used to screen for balance in older people?

**Question 3**: Should the **Mini-Balance Evaluation Systems Test (Mini-BESTest)** be used to screen for balance in older people?

**Question 4**: Should the **Timed up and go (TUG) test** be used to screen for balance in older people?

**Question 5**: Should the **Functional reach (FR) test** be used to screen for balance in older people?

**Question 6**: Should the **Four Square Step Test (FSST)** be used to screen for balance in older people?

**Question 7**: Should the **Brief‐Balance Evaluation Systems Test (Brief‐BESTest)** be used to screen for balance in older people?

**Question 8**: Should the **Functional Gait Assessment (FGA)** be used to screen for balance in older people?

**Question 9**: Should the **Functional Gait Assessment-Brazil (FGA- Brazil)** be used to screen for balance in older people?

**Question 10**: Should the **Fullerton Advanced Balance (FAB) Scale** be used to screen for balance in older people?

**Question 11**: Should the **TURN 180 test** be used to screen for balance in older people?

**Question 12**: Should the **One leg standing (OLS) test** be used to screen for balance in older people?

**Question 13**: Should the **Tinetti's POMA balance subscale** be used to screen for balance in older people?

**Question 14**: Should the **Multi-Directional Reach Test (MDRT)** be used to screen for balance in older people?

**Question 15**: Should the **Wii Balance Board™ (WBB)** be used to screen for balance in older people?

**Question 1**: Should the **Berg Balance Scale (BBS)** be used to screen for balance in older people?

| Outcome (s) | № of studies (№ of patients) | Factors that may decrease certainty of evidence | | | |  | Certainty |
| --- | --- | --- | --- | --- | --- | --- | --- |
|  |  | Risk of bias | Inconsistency | Imprecision | Indirectness | Summary results |  |
| **Reliability** | 7 studies (234) | not serious | not serious | not serious | not serious | ICC = 0.77−0.99  (+) | ⨁⨁⨁⨁ High |
| **Measurement error** | 2 studies (77) | not serious | -------- | serious | not serious | MIC not defined  (?) | -------- |
| **Internal consistency** | 2 studies (294) | not serious | -------- | not serious | not serious | Criteria not met  (?) | -------- |
| **Criterion validity** | 6 studies (407) | not serious | -------- | not serious | not serious | Indeterminate  (?) | -------- |
| **Convergent validity** | 8 studies (689) | not serious | not serious | not serious | not serious | Hypotheses confirmed  (+) | ⨁⨁⨁⨁ High |
| **Discriminative validity** | 7 studies (849) | not serious | not serious | not serious | not serious | Hypotheses confirmed  (+) | ⨁⨁⨁⨁ High |
| **Responsiveness** | 0 studies (0 patients) | -------- | -------- | -------- | -------- | -------- | -------- |

**Question 2**: Should the **Balance Evaluation Systems Test (BESTest)** be used to screen for balance in older people?

| Outcome (s) | № of studies (№ of patients) | Factors that may decrease certainty of evidence | | | |  | Certainty |
| --- | --- | --- | --- | --- | --- | --- | --- |
|  |  | Risk of bias | Inconsistency | Imprecision | Indirectness | Summary results |  |
| **Reliability** | 4 studies (203) | not serious | not serious | not serious | not serious | ICC = 0.77−0.99  (+) | ⨁⨁⨁⨁ High |
| **Measurement error** | 4 studies (203) | not serious | -------- | not serious | not serious | MIC not defined  (?) | -------- |
| **Internal consistency** | 0 studies (0 patients) | -------- | -------- | -------- | -------- | -------- | -------- |
| **Criterion validity** | 2 studies (171) | not serious | not serious | not serious | not serious | r ≥ 0.70  (+) | ⨁⨁⨁⨁ High |
| **Convergent validity** | 3 studies (250) | not serious | not serious | not serious | not serious | Hypotheses confirmed  (+) | ⨁⨁⨁⨁ High |
| **Discriminative validity** | 5 studies (507) | not serious | not serious | not serious | not serious | Hypotheses confirmed  (+) | ⨁⨁⨁⨁ High |
| **Responsiveness** | 0 studies (0 patients) | -------- | -------- | -------- | -------- | -------- | -------- |

**Question 3**: Should the **Mini-Balance Evaluation Systems Test (Mini-BESTest)** be used to screen for balance in older people?

| Outcome (s) | № of studies (№ of patients) | Factors that may decrease certainty of evidence | | | |  | Certainty |
| --- | --- | --- | --- | --- | --- | --- | --- |
|  |  | Risk of bias | Inconsistency | Imprecision | Indirectness | Summary results |  |
| **Reliability** | 3 studies (133) | not serious | not serious | not serious | not serious | ICC = 0.71−0.99  (+) | ⨁⨁⨁⨁ High |
| **Measurement error** | 3 studies (133) | not serious | -------- | not serious | -------- | MIC not defined  (?) | -------- |
| **Internal consistency** | 0 studies (0 patients) | -------- | -------- | -------- | -------- | -------- | -------- |
| **Criterion validity** | 2 studies (171) | not serious | not serious | not serious | not serious | r ≥ 0.83  (+) | ⨁⨁⨁⨁ High |
| **Convergent validity** | 3 studies (250) | not serious | not serious | not serious | not serious | Hypothesis confirmed  (+) | ⨁⨁⨁⨁ High |
| **Discriminative validity** | 5 studies (507) | not serious | serious | not serious | not serious | Inconsistent  (±) | -------- |
| **Responsiveness** | 0 studies (0 patients) | -------- | -------- | -------- | -------- | -------- | -------- |

**Question 4**: Should the **Timed up and go (TUG) test** be used to screen for balance in older people?

| Outcome (s) | № of studies (№ of patients) | Factors that may decrease certainty of evidence | | | |  | Certainty |
| --- | --- | --- | --- | --- | --- | --- | --- |
|  |  | Risk of bias | Inconsistency | Imprecision | Indirectness | Summary results |  |
| **Reliability** | 3 studies (122) | not serious | not serious | not serious | not serious | ICC ≥ 0.83  (+) | ⨁⨁⨁⨁ High |
| **Measurement error** | 1 study (47) | serious | -------- | very serious | -------- | MIC not defined  (?) | -------- |
| **Internal consistency** | 0 studies (0 patients) | -------- | -------- | -------- | -------- | -------- | -------- |
| **Criterion validity** | 1 study (1200) | not serious | -------- | not serious | not serious | r not reported  (?) | -------- |
| **Convergent validity** | 3 studies (1265) | not serious | not serious | not serious | not serious | Hypothesis confirmed  (+) | ⨁⨁⨁⨁ High |
| **Discriminative validity** | 2 studies (1400) | not serious | not serious | not serious | not serious | Hypothesis confirmed  (+) | ⨁⨁⨁⨁ High |
| **Responsiveness** | 1 study (1200) | serious | -------- | not serious | not serious | AUC not reported  (?) | -------- |

**Question 5**: Should the **Functional reach (FR) test** be used to screen for balance in older people?

| Outcome (s) | № of studies (№ of patients) | Factors that may decrease certainty of evidence | | | |  | Certainty |
| --- | --- | --- | --- | --- | --- | --- | --- |
|  |  | Risk of bias | Inconsistency | Imprecision | Indirectness | Summary results |  |
| **Reliability** | 3 studies (128) | not serious | not serious | not serious | not serious | ICC ≥ 0.73  (+) | ⨁⨁⨁⨁ High |
| **Measurement error** | 1 study (47) | serious | -------- | very serious | -------- | MIC not defined  (?) | -------- |
| **Internal consistency** | 0 studies (0 patients) | -------- | -------- | -------- | -------- | -------- | -------- |
| **Criterion validity** | 1 study (1200) | not serious | -------- | not serious | not serious | Indeterminate  (?) | -------- |
| **Convergent validity** | 1 study (1200) | not serious | not serious | not serious | not serious | r = 0.48  (−) | ⨁⨁⨁⨁ High |
| **Discriminative validity** | 1 study (1252) | not serious | not serious | not serious | not serious | Hypothesis confirmed  (+) | ⨁⨁⨁⨁ High |
| **Responsiveness** | 2 studies (1252) | serious | -------- | not serious | not serious | AUC not reported  (?) | -------- |

**Question 6**: Should the **Four Square Step Test (FSST)** be used to screen for balance in older people?

| Outcome (s) | № of studies (№ of patients) | Factors that may decrease certainty of evidence | | | |  | Certainty |
| --- | --- | --- | --- | --- | --- | --- | --- |
|  |  | Risk of bias | Inconsistency | Imprecision | Indirectness | Summary results |  |
| **Reliability** | 2 studies (50) | serious | not serious | serious | not serious | ICC ≥ 0.98  (+) | ⨁⨁◯◯ Low |
| **Measurement error** | 0 studies (0 patients) | -------- | -------- | -------- | -------- | -------- | -------- |
| **Internal consistency** | 0 studies (0 patients) | -------- | -------- | -------- | -------- | -------- | -------- |
| **Criterion validity** | 3 studies (194) | not serious | serious | not serious | not serious | Inconsistent  (±) | -------- |
| **Convergent validity** | 3 studies (194) | not serious | serious | not serious | not serious | Inconsistent  (±) | -------- |
| **Discriminative validity** | 2 studies (114) | not serious | not serious | not serious | not serious | Hypothesis confirmed  (+) | ⨁⨁⨁⨁ High |
| **Responsiveness** | 0 studies (0 patients) | -------- | -------- | -------- | -------- | -------- | -------- |

**Question 7**: Should the **Brief‐Balance Evaluation Systems Test (Brief‐BESTest)** be used to screen for balance in older people?

| Outcome (s) | № of studies (№ of patients) | Factors that may decrease certainty of evidence | | | |  | Certainty |
| --- | --- | --- | --- | --- | --- | --- | --- |
|  |  | Risk of bias | Inconsistency | Imprecision | Indirectness | Summary results |  |
| **Reliability** | 2 studies (77) | not serious | not serious | serious | not serious | ICC = 0.82−0.99  (+) | ⨁⨁⨁◯ Moderate |
| **Measurement error** | 2 studies (77) | not serious | -------- | serious | -------- | MIC not defined  (?) | -------- |
| **Internal consistency** | 0 studies (0 patients) | -------- | -------- | -------- | -------- | -------- | -------- |
| **Criterion validity** | 2 studies (171) | not serious | not serious | not serious | not serious | r_S_ ≥ 0.83  (+) | ⨁⨁⨁⨁ High |
| **Convergent validity** | 3 studies (250) | not serious | not serious | not serious | not serious | Hypothesis confirmed  (+) | ⨁⨁⨁⨁ High |
| **Discriminative validity** | 3 studies (249) | not serious | serious | not serious | not serious | Inconsistent  (±) | -------- |
| **Responsiveness** | 0 studies (0 patients) | -------- | -------- | -------- | -------- | -------- | -------- |

**Question 8**: Should the **Functional Gait Assessment (FGA)** be used to screen for balance in older people?

| Outcome (s) | № of studies (№ of patients) | Factors that may decrease certainty of evidence | | | |  | Certainty |
| --- | --- | --- | --- | --- | --- | --- | --- |
|  |  | Risk of bias | Inconsistency | Imprecision | Indirectness | Summary results |  |
| **Reliability** | 1 study (15) | very serious | not serious | very serious | not serious | ICC ≥ 0.80  (+) | ⨁◯◯◯ Very low |
| **Measurement error** | 0 studies (0 patients) | -------- | -------- | -------- | -------- | -------- | -------- |
| **Internal consistency** | 0 studies (0 patients) | -------- | -------- | -------- | -------- | -------- |  |
| **Criterion validity** | 1 study (35) | not serious | not serious | very serious | not serious | r=0.84  (+) | ⨁⨁◯◯ Low |
| **Convergent validity** | 2 studies (49) | not serious | not serious | very serious | not serious | Hypothesis confirmed  (+) | ⨁⨁◯◯ Low |
| **Discriminative validity** | 1 study (35) | not serious | not serious | very serious | not serious | Hypothesis confirmed  (+) | ⨁⨁◯◯ Low |
| **Responsiveness** | 0 studies (0 patients) | -------- | -------- | -------- | -------- | -------- |  |

**Question 9**: Should the **Functional Gait Assessment-Brazil (FGA- Brazil)** be used to screen for balance in older people?

| Outcome (s) | № of studies (№ of patients) | Factors that may decrease certainty of evidence | | | |  | Certainty |
| --- | --- | --- | --- | --- | --- | --- | --- |
|  |  | Risk of bias | Inconsistency | Imprecision | Indirectness | Summary results |  |
| **Reliability** | 1 study (70) | serious | not serious | serious | not serious | ICC > 0.90  (+) | ⨁⨁◯◯ Low |
| **Measurement error** | 1 study (70) | serious | -------- | serious | -------- | MIC not defined  (?) | -------- |
| **Internal consistency** | 1 study (70) | not serious | -------- | serious | not serious | Criteria not met  (?) | -------- |
| **Criterion validity** | 1 study (121) | not serious | not serious | not serious | not serious | r_S_ = 0.80  (+) | ⨁⨁⨁⨁ High |
| **Convergent validity** | 0 studies () | -------- | -------- | -------- | -------- | -------- | -------- |
| **Discriminative validity** | 1 study (121) | not serious | not serious | not serious | not serious | Hypothesis confirmed  (+) | ⨁⨁⨁⨁ High |
| **Responsiveness** | 0 studies (0 patients) | -------- | -------- | -------- | -------- | -------- | -------- |

**Question 10**: Should the **Fullerton Advanced Balance (FAB) Scale** be used to screen for balance in older people?

| Outcome (s) | № of studies (№ of patients) | Factors that may decrease certainty of evidence | | | |  | Certainty |
| --- | --- | --- | --- | --- | --- | --- | --- |
|  |  | Risk of bias | Inconsistency | Imprecision | Indirectness | Summary results |  |
| **Reliability** | 1 study (31) | very serious | -------- | very serious | not serious | ICC not reported  (?) | -------- |
| **Measurement error** | 0 studies (0 patients) | -------- | -------- | -------- | -------- | -------- | -------- |
| **Internal consistency** | 0 studies (0 patients) | -------- | -------- | -------- | -------- | -------- | -------- |
| **Criterion validity** | 1 study (31) | not serious | not serious | very serious | not serious | r ≥ 0.70  (+) | ⨁⨁◯◯ Low |
| **Convergent validity** | 0 studies (0 patients) | -------- | -------- | -------- | -------- | -------- | -------- |
| **Discriminative validity** | 0 studies (0 patients) | -------- | -------- | -------- | -------- | -------- | -------- |
| **Responsiveness** | 0 studies (0 patients) | -------- | -------- | -------- | -------- | -------- | -------- |

**Question 11**: Should the **TURN 180 test** be used to screen for balance in older people?

| Outcome (s) | № of studies (№ of patients) | Factors that may decrease certainty of evidence | | | |  | Certainty |
| --- | --- | --- | --- | --- | --- | --- | --- |
|  |  | Risk of bias | Inconsistency | Imprecision | Indirectness | Summary results |  |
| **Reliability** | 1 study (66) | very serious | not serious | serious | not serious | ICC = 0.828  (+) | ⨁◯◯◯ Very low |
| **Measurement error** | 1 study (66) | very serious | -------- | serious | not serious | MIC not defined  (?) | -------- |
| **Internal consistency** | 0 studies (0 patients) | -------- | -------- | -------- | -------- | -------- | -------- |
| **Criterion validity** | 1 study (30) | not serious | -------- | very serious | not serious | r not reported  (?) | -------- |
| **Convergent validity** | 0 studies (0 patients) | -------- | -------- | -------- | -------- | -------- | -------- |
| **Discriminative validity** | 1 study (66) | not serious | not serious | serious | not serious | Hypothesis confirmed  (+) | ⨁⨁⨁◯ Moderate |
| **Responsiveness** | 0 studies (0 patients) | -------- | -------- | -------- | -------- | -------- | -------- |

**Question 12**: Should the **One leg standing (OLS) test** be used to screen for balance in older people?

| Outcome (s) | № of studies (№ of patients) | Factors that may decrease certainty of evidence | | | |  | Certainty |
| --- | --- | --- | --- | --- | --- | --- | --- |
|  |  | Risk of bias | Inconsistency | Imprecision | Indirectness | Summary results |  |
| **Reliability** | 2 studies (81) | serious | not serious | serious | not serious | ICC ≥ 0.75  (+) | ⨁⨁◯◯ Low |
| **Measurement error** | 0 studies (0 patients) | -------- | -------- | -------- | -------- | -------- | -------- |
| **Internal consistency** | 0 studies (0 patients) | -------- | -------- | -------- | -------- | -------- | -------- |
| **Criterion validity** | 1 study (1200) | not serious | -------- | not serious | not serious | Indeterminate  (?) | -------- |
| **Convergent validity** | 1 study (1200) | not serious | not serious | not serious | not serious | Hypothesis not confirmed  (−) | ⨁⨁⨁⨁ High |
| **Discriminative validity** | 1 study (1200) | not serious | not serious | not serious | not serious | Hypothesis confirmed  (+) | ⨁⨁⨁⨁ High |
| **Responsiveness** | 1 study (1200) | serious | -------- | not serious | not serious | AUC not reported  (?) | -------- |

**Question 13**: Should the **Tinetti's POMA balance subscale** be used to screen for balance in older people?

| Outcome (s) | № of studies (№ of patients) | Factors that may decrease certainty of evidence | | | |  | Certainty |
| --- | --- | --- | --- | --- | --- | --- | --- |
|  |  | Risk of bias | Inconsistency | Imprecision | Indirectness | Summary results |  |
| **Reliability** | 1 study (60) | very serious | not serious | serious | not serious | ICC ≥ 0.93  (+) | ⨁◯◯◯ Very low |
| **Measurement error** | 0 studies (0 patients) | -------- | -------- | -------- | -------- | -------- | -------- |
| **Internal consistency** | 0 studies (0 patients) | -------- | -------- | -------- | -------- | -------- | -------- |
| **Criterion validity** | 2 studies (1243) | not serious | -------- | not serious | not serious | Indeterminate  (?) | -------- |
| **Convergent validity** | 1 study (1200) | not serious | not serious | not serious | not serious | Hypothesis not confirmed  (−) | ⨁⨁⨁⨁ High |
| **Discriminative validity** | 1 study (1200) | not serious | not serious | not serious | not serious | Hypothesis confirmed  (+) | ⨁⨁⨁⨁ High |
| **Responsiveness** | 1 study (1200) | serious | -------- | not serious | not serious | AUC not reported  (?) | -------- |

**Question 14**: Should the **Multi-Directional Reach Test (MDRT)** be used to screen for balance in older people?

| Outcome (s) | № of studies (№ of patients) | Factors that may decrease certainty of evidence | | | |  | Certainty |
| --- | --- | --- | --- | --- | --- | --- | --- |
|  |  | Risk of bias | Inconsistency | Imprecision | Indirectness | Summary results |  |
| **Reliability** | 2 studies (280) | serious | not serious | not serious | not serious | ICC = 0.83−0.98  (+) | ⨁⨁⨁◯ Moderate |
| **Measurement error** | 0 studies (0 patients) | -------- | -------- | -------- | -------- | -------- | -------- |
| **Internal consistency** | 2 studies (280) | serious | -------- | not serious | not serious | Criteria not met  (?) | -------- |
| **Criterion validity** | 2 studies (280) | not serious | serious | not serious | not serious | Inconsistent  (±) | -------- |
| **Convergent validity** | 2 studies (280) | not serious | serious | not serious | not serious | Inconsistent  (±) | -------- |
| **Discriminative validity** | 1 study (254) | not serious | not serious | not serious | not serious | Hypothesis not confirmed  (−) | ⨁⨁⨁⨁ High |
| **Responsiveness** | 0 studies (0 patients) | -------- | -------- | -------- | -------- | -------- | -------- |

**Question 15**: Should the **Wii Balance Board™ (WBB)** be used to screen for balance in older people?

| Outcome (s) | № of studies (№ of patients) | Factors that may decrease certainty of evidence | | | |  | Certainty |
| --- | --- | --- | --- | --- | --- | --- | --- |
|  |  | Risk of bias | Inconsistency | Imprecision | Indirectness | Summary results |  |
| **Reliability** | 1 study (20) | serious | not serious | very serious | not serious | ICC = 0.64− 0.85  (+) | ⨁◯◯◯ Very low |
| **Measurement error** | 1 study (20) | serious | -------- | very serious | -------- | MIC not defined  (?) | -------- |
| **Internal consistency** | 0 studies (0 patients) | -------- | -------- | -------- | -------- | -------- | -------- |
| **Criterion validity** | 1 study (37) | extremely serious | -------- | very serious | not serious | r not reported  (?) | -------- |
| **Convergent validity** | 1 study (20) | not serious | not serious | very serious | not serious | Hypothesis confirmed  (+) | ⨁⨁◯◯ Low |
| **Discriminative validity** | 0 studies (0 patients) | -------- | -------- | -------- | -------- | -------- | -------- |
| **Responsiveness** | 0 studies (0 patients) | -------- | -------- | -------- | -------- | -------- | -------- |

**GRADE assessment of findings for Muscle strength tools**

**Question 1**: Should the **Lafayette Manual Muscle Tester, Model # 01163 (HHD)** be used to screen for Muscle strength in older people?

**Question 2**: Should the **JAMAR hand-held hydraulic dynamometer** be used to screen for Muscle strength in older people?

**Question 3**: Should the **Nintendo Wii Balance Board (WBB)** be used to screen for Muscle strength in older people?

**Question 4**: Should the **Biodex System 3 isokinetic dynamometer** be used to screen for Muscle strength in older people?

**Question 1**: Should the **Lafayette Manual Muscle Tester, Model # 01163 (HHD)** be used to screen for Muscle strength in older people?

| Outcome (s) | № of studies (№ of patients) | Factors that may decrease certainty of evidence | | | |  | Certainty |
| --- | --- | --- | --- | --- | --- | --- | --- |
|  |  | Risk of bias | Inconsistency | Imprecision | Indirectness | Summary results |  |
| **Reliability** | 2 studies (39) | very serious | not serious | very serious | not serious | ICC = 0.76−0.98  (+) | ⨁◯◯◯ Very low |
| **Measurement error** | 2 studies (39) | very serious | -------- | very serious | not serious | MIC not defined  (?) | -------- |
| **Internal consistency** | 1 study (12) | not serious | -------- | very serious | not serious | Criteria not met  (?) | -------- |
| **Criterion validity** | 2 studies (38) | not serious | not serious | very serious | not serious | r ≥ 0.70  (+) | ⨁⨁◯◯ Low |
| **Convergent validity** | 0 studies (0 patients) | -------- | -------- | -------- | -------- | -------- | -------- |
| **Discriminative validity** | 0 studies (0 patients) | -------- | -------- | -------- | -------- | -------- | -------- |
| **Responsiveness** | 0 studies (0 patients) | -------- | -------- | -------- | -------- | -------- | -------- |

**Question 2**: Should the **JAMAR hand-held hydraulic dynamometer** be used to screen for Muscle strength in older people?

| Outcome (s) | № of studies (№ of patients) | Factors that may decrease certainty of evidence | | | |  | Certainty |
| --- | --- | --- | --- | --- | --- | --- | --- |
|  |  | Risk of bias | Inconsistency | Imprecision | Indirectness | Summary results |  |
| **Reliability** | 2 studies (381) | not serious | not serious | not serious | not serious | ICC = 0.90−0.97  (+) | ⨁⨁⨁⨁ High |
| **Measurement error** | 2 studies (381) | serious | -------- | not serious | not serious | MIC not defined  (?) | -------- |
| **Internal consistency** | 0 studies (0 patients) | -------- | -------- | -------- | -------- | -------- | -------- |
| **Criterion validity** | 0 studies (0 patients) | -------- | -------- | -------- | -------- | -------- | -------- |
| **Convergent validity** | 1 study (281) | not serious | -------- | not serious | not serious | r not reported  (?) | -------- |
| **Discriminative validity** | 0 studies (0 patients) | -------- | -------- | -------- | -------- | -------- | -------- |
| **Responsiveness** | 0 studies (0 patients) | -------- | -------- | -------- | -------- | -------- | -------- |

**Question 3**: Should the **Nintendo Wii Balance Board (WBB)** be used to screen for Muscle strength in older people?

| Outcome (s) | № of studies (№ of patients) | Factors that may decrease certainty of evidence | | | |  | Certainty |
| --- | --- | --- | --- | --- | --- | --- | --- |
|  |  | Risk of bias | Inconsistency | Imprecision | Indirectness | Summary results |  |
| **Reliability** | 2 studies (58) | not serious | not serious | serious | not serious | ICC = 0.96−0.97  (+) | ⨁⨁⨁◯ Moderate |
| **Measurement error** | 2 studies (58) | not serious | -------- | serious | not serious | MIC not defined  (?) | -------- |
| **Internal consistency** | 0 studies (0 patients) | -------- | -------- | -------- | -------- | -------- | -------- |
| **Criterion validity** | 0 studies (0 patients) | -------- | -------- | -------- | -------- | -------- | -------- |
| **Convergent validity** | 2 studies (58) | not serious | not serious | serious | not serious | Hypothesis confirmed  (+) | ⨁⨁⨁◯ Moderate |
| **Discriminative validity** | 0 studies (0 patients) | -------- | -------- | -------- | -------- | -------- | -------- |
| **Responsiveness** | 0 studies (0 patients) | -------- | -------- | -------- | -------- | -------- | -------- |

**Question 4**: Should the **Biodex System 3 isokinetic dynamometer** be used to screen for Muscle strength in older people?

| Outcome (s) | № of studies (№ of patients) | Factors that may decrease certainty of evidence | | | |  | Certainty |
| --- | --- | --- | --- | --- | --- | --- | --- |
|  |  | Risk of bias | Inconsistency | Imprecision | Indirectness | Summary results |  |
| **Reliability** | 2 studies (49) | not serious | not serious | very serious | not serious | ICC ≥ 70  (+) | ⨁⨁◯◯ Low |
| **Measurement error** | 2 studies (49) | serious | -------- | very serious | not serious | MIC not defined  (?) | -------- |
| **Internal consistency** | 0 studies (0 patients) | -------- | -------- | -------- | -------- | -------- | -------- |
| **Criterion validity** | 0 studies (0 patients) | -------- | -------- | -------- | -------- | -------- | -------- |
| **Convergent validity** | 0 studies (0 patients) | -------- | -------- | -------- | -------- | -------- | -------- |
| **Discriminative validity** | 0 studies (0 patients) | -------- | -------- | -------- | -------- | -------- | -------- |
| **Responsiveness** | 0 studies (0 patients) | -------- | -------- | -------- | -------- | -------- | -------- |
